# Hepatocellular Carcinomas Originate Predominantly from Hepatocytes and Benign Lesions from Hepatic Progenitor Cells

**DOI:** 10.1016/j.celrep.2017.03.059

**Published:** 2017-04-19

**Authors:** Krishna S. Tummala, Marta Brandt, Ana Teijeiro, Osvaldo Graña, Robert F. Schwabe, Cristian Perna, Nabil Djouder

**Affiliations:** 1 Cancer Cell Biology Programme, Growth Factors, Nutrients and Cancer Group, Centro Nacional de Investigaciones Oncológicas (CNIO), Madrid 28029, Spain; 2 Structural Biology and Biocomputing Programme, Bioinformatics Unit, Centro Nacional de Investigaciones Oncológicas (CNIO), Madrid 28029, Spain; 3 Department of Medicine, Columbia University, New York, NY 10032, USA; 4 Department of Pathology, Hospital Universitario Ramón y Cajal, IRYCIS, Madrid 28034, Spain

**Keywords:** hepatic progenitor cells, hepatocytes, lineage tracking, HCC, adenomas, regenerative nodules, galectin-3, α-ketoglutarate, NAD^+^, DNA damage

## Abstract

Hepatocellular carcinoma (HCC) is an aggressive primary liver cancer. However, its origin remains a debated question. Using human data and various hepatocarcinogenesis mouse models, we show that, in early stages, transformed hepatocytes, independent of their proliferation status, activate hepatic progenitor cell (HPC) expansion. Genetic lineage tracing of HPCs and hepatocytes reveals that, in all models, HCC originates from hepatocytes. However, whereas in various models tumors do not emanate from HPCs, tracking of progenitors in a model mimicking human hepatocarcinogenesis indicates that HPCs can generate benign lesions (regenerative nodules and adenomas) and aggressive HCCs. Mechanistically, galectin-3 and α-ketoglutarate paracrine signals emanating from oncogene-expressing hepatocytes instruct HPCs toward HCCs. α-Ketoglutarate preserves an HPC undifferentiated state, and galectin-3 maintains HPC stemness, expansion, and aggressiveness. Pharmacological or genetic blockage of galectin-3 reduces HCC, and its expression in human HCC correlates with poor survival. Our findings may have clinical implications for liver regeneration and HCC therapy.

## Highlights

• HPCs expand in the early stages of hepatocarcinogenesis
• HCC predominantly originates from hepatocytes
• Oncogene-induced hepatocyte transformation activates HPCs via galectin-3 and αKG
• Activated HPCs contribute to liver tumor heterogeneity

## Graphical Abstract

**Figure:**
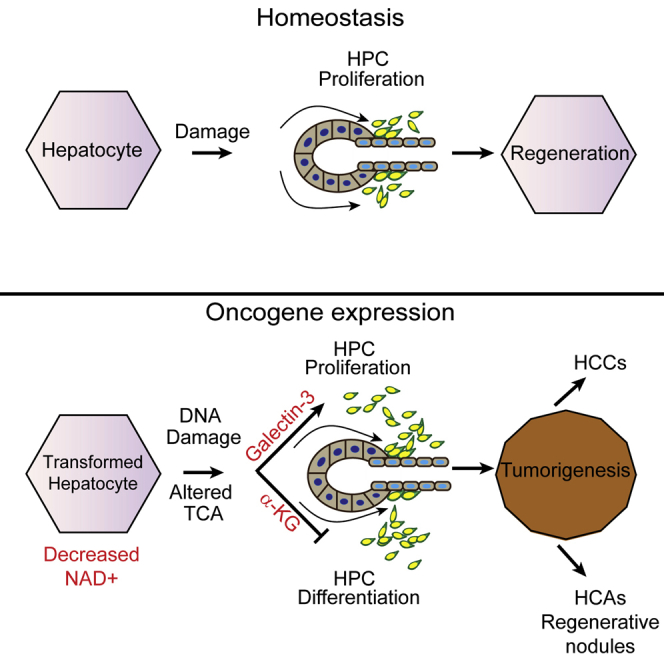

## Teaser

Tummala et al. demonstrate that hepatocytes and hepatic progenitor cells participate in liver tumor heterogeneity. Malignant hepatocytes crosstalk and activate the neighboring progenitor cells through releases of galectin-3 and α-ketoglutarate. Blocking galectin-3 reduces hepatocarcinogenesis and may have important clinical implications.

## Introduction

Hepatocellular carcinoma (HCC) and intrahepatic cholangiocarcinoma (ICC) are the most lethal primary forms of liver cancers. Rarer benign hepatic tumors include focal nodular hyperplasias and hepatocellular adenomas (HCAs), which may progress to HCC (Pilati et al., 2014). Regenerative nodules (RN), common in cirrhotic liver, are also likely HCC precursors (Lin et al., 2010).

Hepatocytes represent the most functionally active parenchymal liver cells (>80%). Others are the bile duct epithelial cells or ductular cells (DCs). DCs originate from the canal of Hering and are considered to be the bipotential hepatic progenitor cells (HPCs) also known as transit-amplifying cells or oval cells in rodent models or liver progenitor cells in humans. The canal of Hering is therefore considered the putative hepatic stem cell niche in the adult liver. HPCs have a transcriptomic profile similar to DCs (He et al., 2013) as well as the ability to replenish damaged hepatocytes and cholangiocytes (Carpentier et al., 2011, Espanol-Suner et al., 2012, Lu et al., 2015, Wang et al., 2003).

The liver has remarkable plasticity following injury and is the only adult organ with complete regenerative potential. Depending on the injury (acute or chronic), liver regeneration may involve activation of either fully differentiated hepatocytes or HPCs. Fate-tracing studies indicate that hepatocytes are mainly responsible for liver maintenance under normal conditions and responses to acute liver injury (Malato et al., 2011). Acute liver damage, partial hepatectomy or chemical damage by carbon tetrachloride (CCl_4_), may induce replication of mature hepatocytes, which replenishes the liver and maintains its homeostasis.

Impairment of hepatocyte replicative potential can induce the emergence of HPCs, which are required to continuously supply new hepatocytes and maintain the organ’s functional integrity during chronic liver damage. HPC expansion is mainly due to various pathologies, environmental or genetic etiologies of human HCC, primarily affecting hepatocytes and leading to chronic liver damage in the early stages of tumorigenesis (e.g., fibrosis, cirrhosis, steatosis, inflammation, viral infection, aflatoxin, α-1 antitrypsin deficiency, Wilson’s disease, and hemochromatosis). HCC progression is thus often accompanied by HPC activation, but mechanisms initiated by injured hepatocytes remain still elusive.

Cancer stem cell markers (EpCam, CD133, and CD44), specific cytokeratins (CKs; 7 and 19), and the sex-determining region Y-box (SRY-box) containing the transcription factor Sox9 (Furuyama et al., 2011, Rountree et al., 2012, Yamashita et al., 2009) aid recognition of DCs and HPCs. DCs and HPCs remain negative for hepatocyte nuclear factor 4 alpha (HNF4α), a marker of mature hepatocytes and hybrid periportal hepatocytes (HybHPs) (Font-Burgada et al., 2015). A recent report indicated that HybHPs expressing both Sox9 and HNF4α may participate in liver regeneration, but not HCC (Font-Burgada et al., 2015). Long-debated uncertainties are thus whether DCs and HPCs are hepatic cancer stem cells (CSCs), and whether they participate in hepatocarcinogenesis. According to recent proposals, cells with similar transcriptomic profiles to oval cells may be HCC progenitors (He et al., 2013, Holczbauer et al., 2013). Furthermore, liver carcinomas derived from genetically modified bipotential liver progenitors resemble features of the human disease (Zender et al., 2005). Recently, hepatocytes were suggested to be the sole cell of origin for HCC (Mu et al., 2015), and HPCs or DCs do not produce tumors in genetic and chemical HCC mouse models (Jörs et al., 2015, Mu et al., 2015). Certainly, due to scarcities of animal models recapitulating human liver tumorigenesis, the cells of origin of liver cancer as well as the origin of its heterogeneity (benign lesions, RN-induced cirrhosis, HCAs, and HCCs) are not clear yet and may be context dependent.

A mouse model (hURI-tetOFF^hep^) we recently generated expresses hepatocyte-specific human unconventional prefoldin RPB5 interactor (hURI). In this model, NAD^+^-deficit-induced DNA damage causes multistep liver tumor development somehow mimicking human hepatocarcinogenesis, with development of focal nodular hyperplasia, RN, non-alcoholic steatohepatitis (NASH), HCAs, and HCCs (Gomes et al., 2016, Tummala et al., 2014). Using this model and several other models, combined with genetic lineage tracing and in vitro co-culture cell experiments, we evaluate here the contribution of hepatocytes and HPCs in hepatocarcinogenesis. We also report mechanisms emerging from hepatocytes and regulating HPC expansion in the early stages of tumorigenesis, through metabolic paracrine signals, implicating galectin-3 and α-ketoglutarate.

## Results

### hURI-tetOFF^hep^ Mice Display HPC Signatures in the Early Stages of Hepatocarcinogenesis Correlating with Aggressive Human HCC

**Figure 1:**
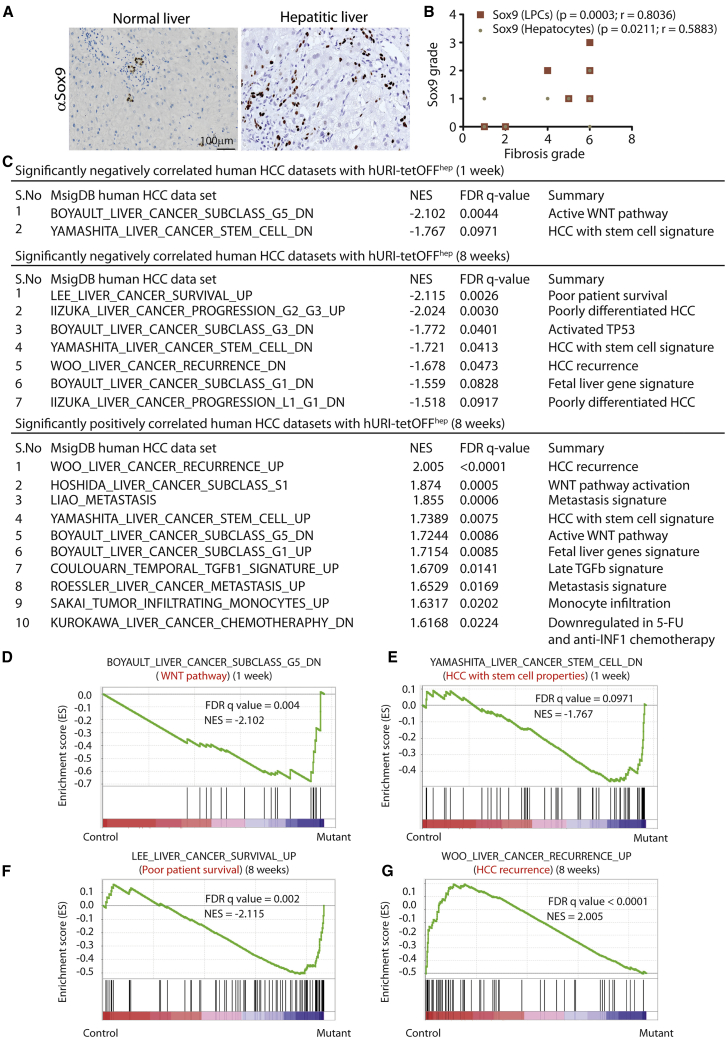
hURI-tetOFF^hep^ Mice Display HPC Signatures in Early Hepatocarcinogenesis Stages Correlating with Aggressive Human HCC (A) Representative images of Sox9 immunohistochemistry (IHC) in human hepatitis samples. Sox9 expression was evaluated in the liver progenitor cells (LPCs) and hepatocytes (n = 15). (B) Graph representing the correlation between fibrosis grade and Sox9 expression in human hepatitis samples (n = 15). (C) Table summarizing the most significantly enriched human HCC gene sets in 1- or 8-week-old hURI-tetOFF^hep^ mice. (D) GSEA of human HCC WNT pathway and 1-week-old hURI-tetOFF^hep^ mice protein signature. (E) GSEA of human HCC stem cell properties and 1-week-old hURI-tetOFF^hep^ mice protein signature. (F) GSEA of human HCC poor patient survival and 8-week-old hURI-tetOFF^hep^ mice protein signature. (G) GSEA of human HCC recurrence and 8-week-old hURI-tetOFF^hep^ mice protein signature. All protein signature datasets obtained for hURI-tetOFF^hep^ mice were previously described and achieved by iTRAQ (Tummala et al., 2014). Normalized enrichment score (NES) and false discovery rate (FDR) q-values are indicated in each graph. Scale bar represents 100 μm.

Human HCC develops via pre-neoplastic lesions that frequently arise within a background of chronic inflammatory liver damage or hepatitis progressing to fibrosis, cirrhosis, and eventually HCC. Premalignant lesions contain Sox9-positive progenitors (considered to be HPCs) (He et al., 2013). Sox9 expression correlates with degrees of liver-damage-associated fibrosis, according to immunohistochemistry (IHC) of human hepatitic samples (Figures 1A and 1B). Thus, Sox9-positive cells (SPCs) expand in early human hepatocarcinogenesis.

Using public expression data applied to classify human HCC subtypes (Figures 1 and S1), we identified gene sets that are up- or downregulated in hURI-tetOFF^hep^ livers in early tumorigenesis stages (Tummala et al., 2014) and correlate with therapy resistance and poor prognosis according to gene set enrichment analysis (GSEA). Normalized enrichment scores and false discovery rates indicated that 1-week-old hURI-tetOFF^hep^ mice have a signature very similar to G5 subtype HCC, which is strongly related to β-catenin mutations and thus WNT activation (Boyault et al., 2007), which is essential for HPC expansion (Figures 1C and 1D). Further evaluation showed that an HPC signature associated with WNT/β-catenin signaling is significantly enriched in 1- and 8-week-old hURI-tetOFF^hep^ mice (Figures 1C and 1E) (Yamashita et al., 2009).

GSEA also detected: significant overlaps between transcriptomic signatures of 8-week-old hURI-tetOFF^hep^ mouse and the S1 HCC subclass, confirming that in early stages the model exhibits an aberrant WNT signaling pathway signature (Hoshida et al., 2009) (Figures 1C and S1A); fetal liver and p53 pathway activation signatures, respectively present in G1 and G3 subtypes (Boyault et al., 2007) (Figures 1C and S1B); and a signature associated with transforming growth factor β (TGF-β) signaling pathways that reportedly activate HPCs (Coulouarn et al., 2008) (Figure 1C).

Significant enrichment of genes associated with poor patient survival, including stem cell genes, was detected in data from 8-week-old hURI-tetOFF^hep^ mice (Figures 1C and 1F) (Lee et al., 2004) and a metastasis gene signature predicting tumor relapse in early-stage HCC patients (Figures 1C and S1C) (Liao et al., 2008, Roessler et al., 2010). Further analysis revealed that these stem-like liver cancer subtypes represent a class of poorly differentiated HCC (Figures 1C and S1D) (Iizuka et al., 2005). Moreover, stem-like liver cancers from relapsing patients showed a signature enriched in 8-week-old hURI-tetOFF^hep^ mice (Figures 1C and 1G) (Woo et al., 2008). Thus, in early stages, hURI-tetOFF^hep^ livers display a stem cell-like signature found in aggressive human HCC.

### HPCs Expand in the Early Stages of Hepatocarcinogenesis

**Figure 2:**
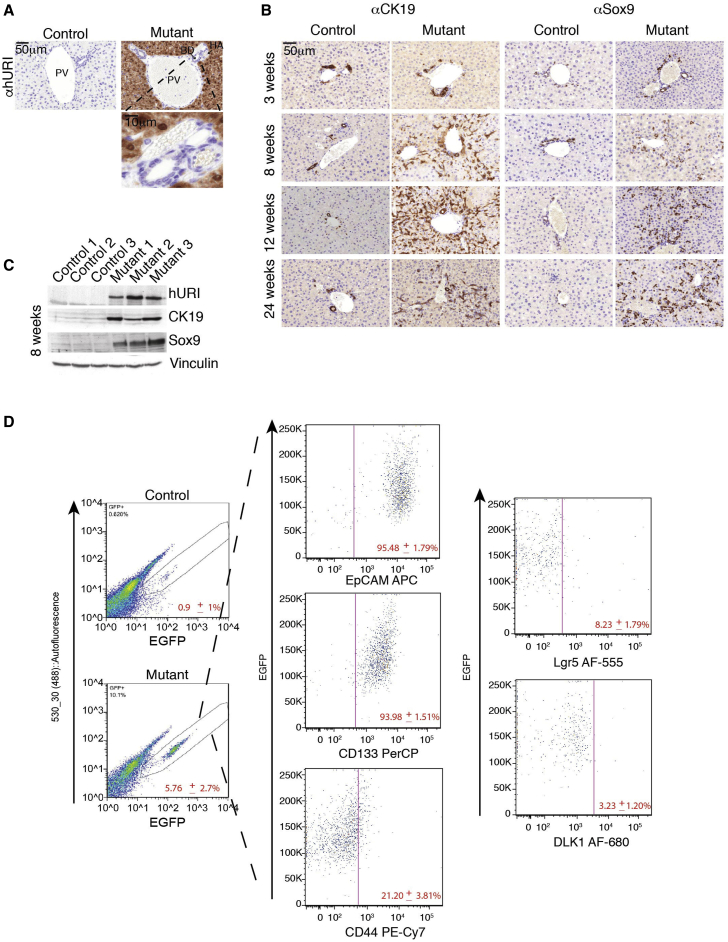
HPCs Expand in the Early Stages of Hepatocarcinogenesis (A) IHC of 1-week-old hURI-tetOFF^hep^ mouse livers using an antibody recognizing specifically hURI. HA, hepatic artery; BD, bile duct; PV, portal vein. (B) Sox9 and CK19 IHC in liver sections derived from 3-, 8-, 12-, and 24-week-old hURI-tetOFF^hep^ mice. (C) Western blot (WB) of liver lysates from 8-week-old hURI-tetOFF^hep^ mice. Membranes were blotted with the indicated antibodies. (D) FACS of EGFP-positive cells isolated from hURI-tetOFF^hep^ mouse crossed with Sox9^IRES-EGFP^ line. SPCs (EGFP positive) were then analyzed for expression of the indicated markers (EpCAM, CD133, CD44, Lgr5, and DLK1) (n = 6). Scale bars represent 50 μm and 10 μm.

Upregulated proteins identified in published proteomic (isobaric tags for relative and absolute quantification [iTRAQ]) profiles of 8-week-old hURI-tetOFF^hep^ mouse livers (Tummala et al., 2014) included several DC markers and proteins of the extracellular matrix (ECM) (e.g., laminin, vimentin, and transgelin) essential for oval cell expansion (Figure S2A). Notably, hURI was specifically expressed in hepatocytes, but not in DCs (Figure 2A). Thus, hURI-tetOFF^hep^ mice were crossed with Sox9^IRES-EGFP^ mice expressing EGFP from the *Sox9* promoter primarily in DCs (Supplemental Experimental Procedures). EGFP/SPCs were isolated by fluorescence-activated cell sorting (FACS). qRT-PCR of isolated EGFP-positive cells and whole mutant livers (hereafter, “mutants” refers to mice ectopically expressing hURI) confirmed that hURI is specifically expressed in hepatocytes (Figure S2B). Interestingly, IHC and western blot (WB) of Sox9 and CK19 markers confirmed the presence of a ductular reaction in mutant livers (Figures 2B, 2C, and S2C). We detected DC expansion in mutant livers when preneoplastic lesions were apparent, in 8- to 24-week-old mutant livers, but not in non-pathological 3-week-old livers expressing hURI (Figure 2B). Importantly, increased laminin was confirmed by IHC (Figures S2D and S2E). SPCs also expanded in 7-week-old C57BL/6 mice treated with the diethylnitrosamine (DEN) carcinogen known to induce HCC (Figures S2F and S2G) (Tummala et al., 2014). Thus, SPCs expand during liver tumorigenesis.

Co-immunofluorescence (co-IF) using Sox9 and CK19 antibodies in hURI-tetOFF^hep^ liver sections revealed that out of the total number of cells expressing either Sox9 or CK19, ∼15% were positive for only Sox9, 60% were CK19 positive, and 30% were positive for both (Figures S2H and S2I). Thus, SPCs comprise a small subset of the highly heterogeneous DC population.

We subsequently checked other DC/HPC markers by FACS-sorting EGFP^+^ SPCs from liver cells of 12-week-old mice generated from an hURI-tetOFF^hep^ and Sox9^IRES-EGFP^ cross (Supplemental Experimental Procedures). The expanded EGFP^+^ SPCs in mutant mice represented 5.76% ± 2.7% of the liver fraction excluding hepatocytes but only 0.9% ± 1% in their littermates (Figure 2D). EGFP cells were positive for the CSC markers EpCAM, CD133, and CD44 (95.5% ± 1.79%; 94.0% ± 1.51%, and 21.2% ± 3.81%, respectively). However, a small proportion of EGFP^+^ SPCs was positive for LGR5 (8.23% ± 1.79%) (Huch et al., 2013b) and DLK1 (3.23% ± 1.20%) (Xu et al., 2012) markers (Figure 2D). SPCs thus represent a heterogeneous DC population with stem cell characteristics and may be considered as hepatic CSCs or HPCs.

### HPCs Contribute to Liver Tumorigenesis

**Figure 3:**
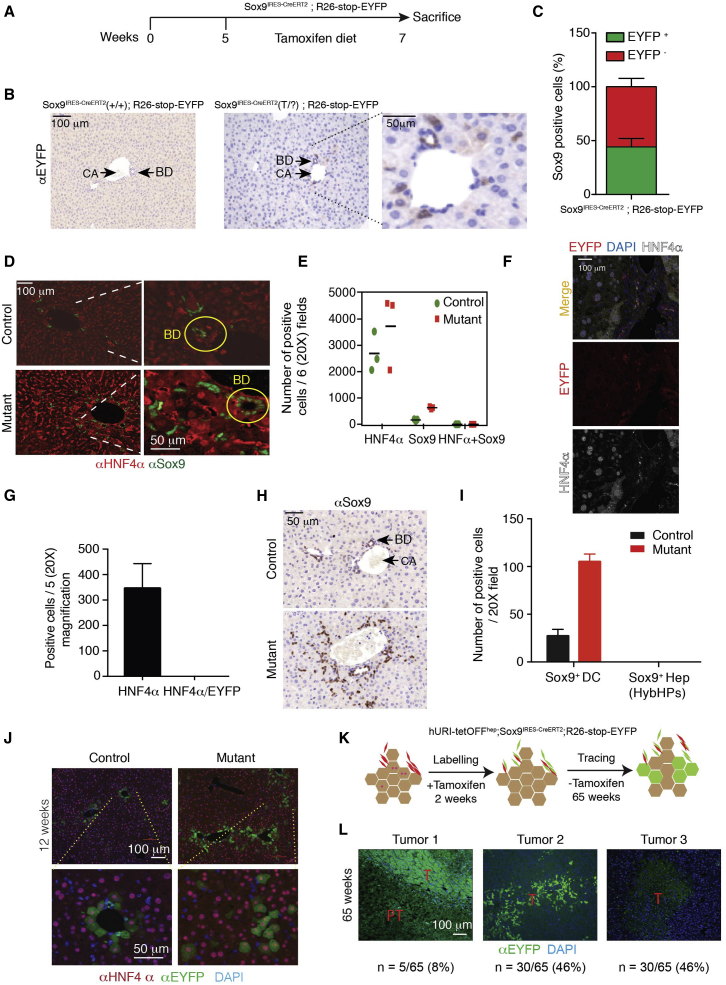
HPCs Contribute to Liver Tumorigenesis (A) Schematic representation of tamoxifen-treated Sox9^IRES-creERT2^; R26-stop-EYFP. 5-week-old mice were fed with tamoxifen diet for 2 weeks and then sacrificed at 7 weeks of age. (B) EYFP IHC in liver sections derived from 7-week-old Sox9^IRES-creERT2^; R26-stop-EYFP mice treated as described in (A) (n = 3). (C) Quantification of co-IF of Sox9 and EYFP in mice treated as described in (A) (n = 3). (D) Co-IF of Sox9 and HNF4α in Sox9^IRES-creERT2^; R26-stop-EYFP mice. n = 3. BD, bile duct. (E) Quantification of (D) (n = 3). (F) Representative images of confocal microscopy of EYFP and HNF4α co-IF in 4-week old Sox9^IRES-creERT2^; R26-stop-EYFP mice. (G) Quantification of (F) (n = 5 mice). (H) Sox9 IHC in liver sections derived from 12-week-old hURI-tetOFF^hep^ mice (n = 5). (I) Quantification of (G) (n = 5). (J) Co-IF of HNF4α and EYFP in liver sections derived from 12-week-old hURI-tetOFF^hep^; Sox9^IRES-creERT2^; R26-stop-EYFP mice (n = 5). (K) Schematic representation of tamoxifen-treated hURI-tetOFF^hep^; Sox9^IRES-creERT2^; R26-stop-EYFP mice. 5-week-old mice were fed with tamoxifen diet for 2 weeks and sacrificed at 65 weeks of age. (L) Representative immunofluorescence images of three different EYFP staining patterns observed in tumors from hURI-tetOFF^hep^; Sox9^IRES-creERT2^; R26-stop-EYFP mice treated as described in (K). T, tumor; PT, peritumor; n = 65 tumors. Data are presented as mean ± SEM. Scale bars represent 100 μm and 50 μm.

Next, we tracked SPCs during liver tumorigenesis by crossing Sox9^IRES-CreERT2^ and reporter R26-stop-EYFP. In this context, SPCs express an inducible Cre recombinase, which specifically deletes the *loxP*-flanked stop cassette in the Rosa26R reporter locus. This occurs in genomic DNA, so all descendants of labeled cells inherit enhanced yellow fluorescent protein (EYFP) expression. For confirmation, 5-week-old mice were fed a tamoxifen diet for 2 weeks and then transferred to chow (Figure 3A). Tamoxifen-activated Cre recombinase permanently EYFP-labeled the emerging SPCs (Figure 3B). Importantly, the tamoxifen dose (400 mg/kg supplied in food) and exposure time (2 weeks) did not induce the hepatocyte toxicity known to increase Sox9 levels in hepatocytes as previously reported (Carpentier et al., 2011) (Figure 3B). Co-IF of Sox9 and EYFP revealed a 44.2% ± 7.7% recombination efficiency (Figure 3C). Therefore, only about 40% of the SPCs are genetically tracked in this model. Importantly, after 2 weeks of tamoxifen treatment, we did not detect any cells positive for Sox9 and HNF4α (a marker of mature hepatocytes) in Sox9^IRES-creERT2^; R26-stop-EYFP mice (Figures 3D and 3E). Confocal microscopy and quantification confirmed that EYFP and HNF4α did not co-localize (Figures 3F and 3G). Additionally, Sox9 expression was solely restricted to SPCs in the hURI-tetOFF^hep^ model after 2 weeks of tamoxifen treatment (Figures 3H and 3I). Importantly, we did not detect the presence of hybrid periportal hepatocytes (HybHPs) positive for both Sox9 and HNF4α, which have been reported to participate in liver regeneration, but not HCC (Font-Burgada et al., 2015). Therefore, only cells positive for Sox9 but negative for HNF4α were tracked in this study. Moreover, Sox9 induction due to hepatocyte toxicity was definitely excluded.

Crosses of hURI-tetOFF^hep^ and Sox9^IRES-CreERT2^; R26-stop-EYFP lines showed that at 12 weeks of age, SPCs gave rise to periportal hepatocytes expressing HNF4α and EYFP in control mice (Figures 3J and S3A), suggesting that SPCs supply new periportal hepatocytes (Carpentier et al., 2011). In 12-week-old mutants, newly formed hepatocytes (HNF4α and EYFP positive) invaded the parenchyma (Figure 3J). At 35 weeks of age, we observed EYFP-positive anisokaryotic hepatocyte clusters in the liver periportal region (Figure S3B). Notably, in mutant mice, EYFP-positive hepatocytes derived from SPCs expanded to the parenchyma, and those reaching the central vein expressed cytochrome P450 2E1, indicating that SPCs produce fully differentiated and metabolically functional hepatocytes (Figure S3C). In 65-week-old mice, some tumors were completely EYFP positive (8%), suggesting SPC origin, but in some, EYFP signals were heterogeneous (46%) or non-detectable (46%) (Figures 3K and 3L, tumors 1–3). Thus, SPCs are not the only cells at the origin of liver cancer.

### Hepatocytes Contribute to Liver Tumorigenesis

**Figure 4:**
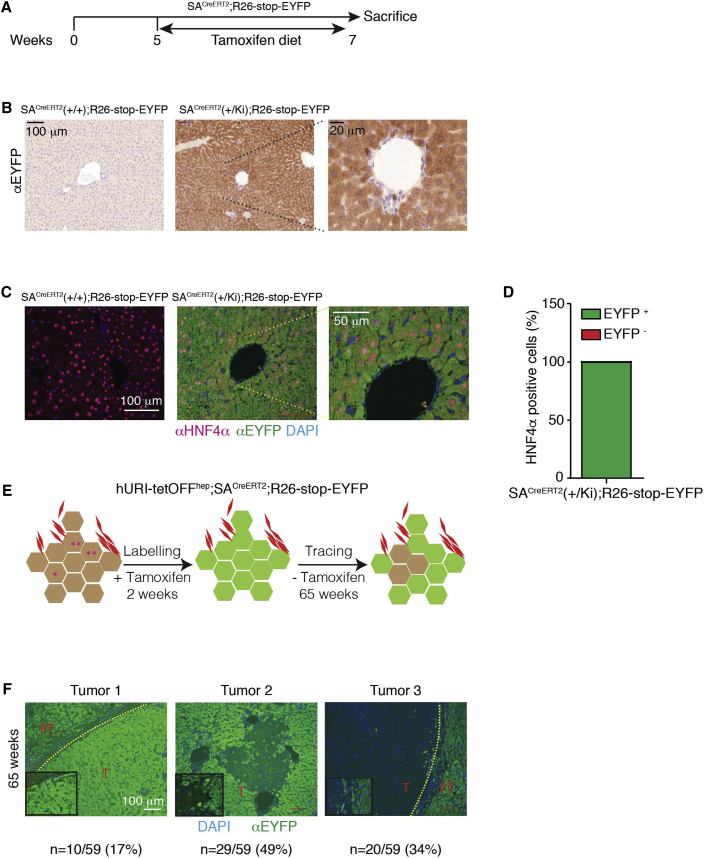
Hepatocytes Contribute to Liver Tumorigenesis (A) Schematic representation of tamoxifen-treated SA^creERT2^; R26-stop-EYFP mice. 5-week-old mice were fed with tamoxifen diet for 2 weeks and sacrificed at 7 weeks of age. (B) Representative images of EYFP IHC performed in liver sections derived from SA^creERT2^; R26-stop-EYFP mice treated as described in (A) (n = 3). (C) Co-IF images of HNF4α and EYFP in liver sections derived from mice treated as described in (A) (n = 3). (D) Quantification of percentage of hepatocytes positive for both HNF4α and EYFP, as described in (C) (n = 3). (E) Schematic representation of tamoxifen-treated hURI-tetOFF^hep^; SA^creERT2^; R26-stop-EYFP mice. 5-week-old mice were treated for 2 weeks with tamoxifen and sacrificed at 65 weeks of age. (F) Representative IF images of three different EYFP staining pattern observed in tumors from hURI-tetOFF^hep^; SA^creERT2^; R26-stop-EYFP mice treated as described in (E); n = 16 mice and 59 tumors. Scale bars represent 100 μm, 50 μm, and 20 μm.

The above observations, together with previous findings showing that genotoxic stress in hepatocytes initiates hepatocarcinogenesis in hURI-tetOFF^hep^ and DEN-treated mice (Tummala et al., 2014), suggest that some tumors in these models may derive from hepatocytes. Thus, we followed hepatocyte fate during hepatocarcinogenesis using mice obtained by crossing serum albumin (SA)^CreERT2^ and R26-stop-EYFP reporter lines (Tummala et al., 2014). In this context, Cre recombinase activation enables expression of EYFP in all hepatocyte progeny. Recombination efficiency was verified after tamoxifen treatment (Figure 4A). IHC revealed that the EYFP signal was restricted to all hepatcoytes (Figure 4B). No signal was observed in DCs or HPCs (Figure 4B). Co-IF of EYFP and the hepatocytic marker HNF4α revealed a recombination efficiency of 99.95% ± 0.05% (Figures 4C and 4D), suggesting almost complete hepatocyte labeling.

Next, we crossed hURI-tetOFF^hep^ and SA^CreERT2^; R26-stop-EYFP lines (hereafter “crosses”) (Figure 4E). At 65 weeks of age, immunofluorescence (IF) of liver sections from crosses revealed that the whole tissue (and tumors) was EYFP-positive (Figure 4F, tumor 1; 17% of all cases), suggesting that hepatocytes contribute to hepatocarcinogenesis. However, in some cases, while peritumoral counterparts were EYFP positive, tumors were highly heterogeneous mixtures of EYFP-positive and EYFP-negative hepatocytes (Figure 4F, tumor 2; 49% of all cases), some were even completely EYFP-negative (Figure 4F, tumor 3; 34% of all cases). We detected similar trends in DEN-treated 35-week-old SA^CreERT2^; R26-stop-EYFP mice (Figures S4A and S4B). Notably, recombination efficiency in DEN-treated SA^CreERT2^; R26-stop-EYFP mice was about 100% (98.1% ± 1.6%) (Figures S4C and S4D). Therefore, hepatocytes participate in liver tumorigenesis but are not the exclusive origin of liver tumors.

We also checked whether EYFP and Sox9 costained in 12-week-old hURI-tetOFF^hep^ and SA^CreERT2^; R26-stop-EYFP mice. No co-IF was detected in liver sections from crosses, indicating that hepatocyte dedifferentiation may not give rise to SPCs (Figures S4E and S4F).

### HCC Predominantly Originates from Hepatocytes, and HPCs Contribute to Liver Tumor Heterogeneity

**Figure 5:**
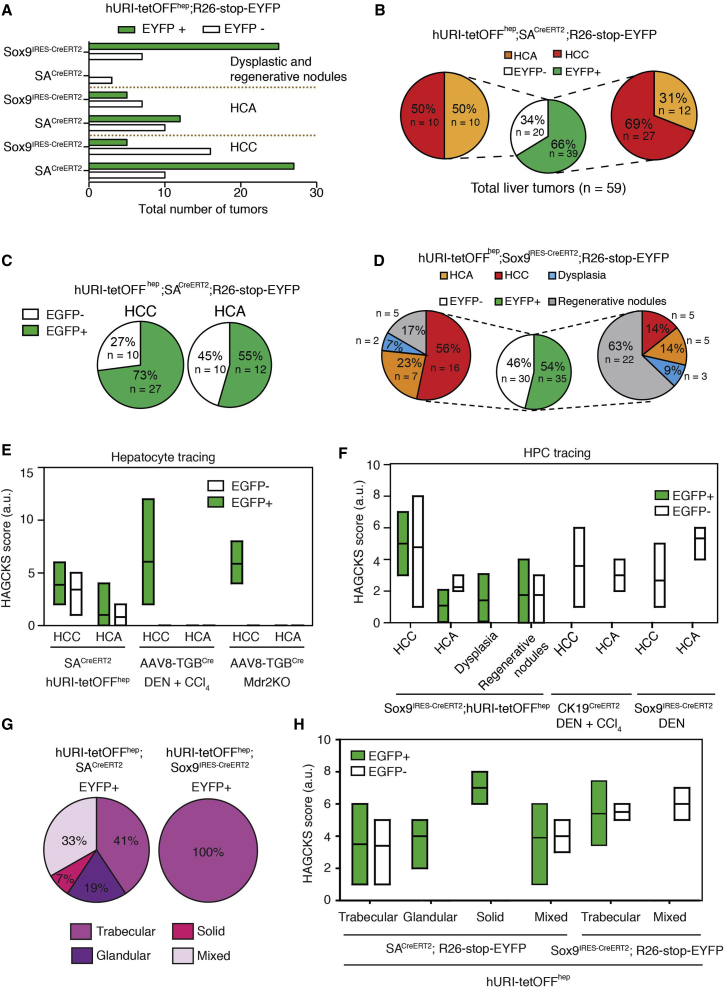
HCC Predominantly Originates from Hepatocytes, and HPCs Contribute to Liver Tumor Heterogeneity (A) Graph representing normalized number of EGFP-positive and EGFP-negative dysplastic and RNs, HCAs, and HCCs derived from hURI-tetOFF^hep^ crossed with SA^CreERT2^; R26-stop-EYFP and hURI-tetOFF^hep^ crossed with Sox9^CreERT2^; R26-stop-EYFP. (B) Central pie chart representing total liver tumors derived from hURI-tetOFF^hep^ crossed with SA^CreERT2^; R26-stop-EYFP. Right pie chart represents the percentage of HCCs and HCAs derived from hepatocytes. Left pie chart represents the percentage of HCCs and HCAs derived from the non-hepatocytic population. (C) Left pie chart represents the total number of HCCs positive or negative for EYFP and derived from hURI-tetOFF^hep^ crossed with SA^CreERT2^; R26-stop-EYFP mice. Right pie chart represents the total number of liver HCAs positive or negative for EYFP and derived from hURI-tetOFF^hep^ crossed with SA^CreERT2^; R26-stop-EYFP mice. (D) Central pie chart representing normalized number of liver tumors derived from hURI-tetOFF^hep^ crossed with Sox9^IRES-CreERT2^; R26-stop-EYFP mice. Right pie chart represents the normalized percentage of liver lesions derived from HPCs. Left pie chart represents the percentage of liver lesions derived from a distinct population than HPCs. (E) HAGCKS score for EGFP-positive and EGFP-negative HCCs or HCAs obtained after hepatocyte tracing in hURI-tetOFF^hep^ crossed with SA^CreERT2^; R26-stop-EYFP mice (n = 59), R26-mTOM-stop-mGFP mice infected with AAV8-Tgb^Cre^ and treated with DEN and CCl_4_ (n = 42), and Mdr2^KO^ mice crossed with R26-stop-ZsGreen mice and infected with AAV8-Tgb^Cre^ (n = 7). (F) HAGCKS score for EGFP-positive and EGFP-negative HCC, HCA, dysplasia or RNs obtained after HPC tracing in hURI-tetOFF^hep^ crossed with Sox9^CreERT2^; R26-stop-EYFP mice (n = 65), CK19^CreERT2^; R26-mTOM-stop-mGFP mice treated with DEN and CCl_4_ (n = 8), and Sox9^CreERT2^; R26-stop-EYFP mice treated with DEN (n = 6). (G) Pie charts representing type of HCC derived from hepatocytes in SA^CreERT2^; R26-stop-EYFP mice (left chart), or HPCs in Sox9^IRES-CreERT2^; R26-stop-EYFP mice (right chart). (H) HAGCKS score for EGFP-positive and EGFP-negative HCCs derived from hURI-tetOFF^hep^ crossed with SA^CreERT2^; R26-stop-EYFP mice (n = 59), and hURI-tetOFF^hep^ crossed with Sox9^CreERT2^; R26-stop-EYFP mice (n = 65) and plotted according to tumor type. Data are presented as mean ± SEM.

To determine the contributions of hepatocytes and HPCs to HCC and/or HCA, we evaluated the proportions and types of tumors they predominantly formed. Tumors derived from SA^CreERT2^; R26-stop-EYFP or Sox9^IRES-CreERT2^; R26-stop-EYFP crosses were first histopathologically classified as either HCC or HCA according to World Health Organization criteria (WHO, 2008). Interestingly, hepatocytes and HPCs participate in liver tumor heterogeneity, giving rise to RNs, HCAs, and HCCs (Figures 5A and S5A–S5C). Normalization of number of tumors to the percentage of recombination indicated that 66% of EYFP-positive tumors originated from labeled hepatocytes. Interestingly, 69% and 31% of EYFP-positive tumors (66% of total) derived from hepatocytes were HCC and HCA, respectively (Figure 5B). Half of the EYFP-negative tumors were HCC and half were HCA, suggesting that they have distinct cell origins, possibly some from SPCs. Notably, 73% and 27% of all HCCs analyzed from SA^CreERT2^; R26-stop-EYFP crosses originated from hepatocytes and non-hepatocytic cells, respectively (Figure 5C). Almost similar percentages of the HCAs originated from hepatocytic and non-hepatocytic cells (Figure 5C). Thus, hepatocytes principally contribute to HCCs.

Most EYFP-positive lesions (54% of total) derived from HPCs were benign (Figure 5D), including dysplasia (9%), RNs (63%), and HCA (14%). Surprisingly, only 14% HCCs were detected, possibly originating directly from HPCs or transiting from the RNs or adenomas known to be HCC precursors (Pilati et al., 2014). Notably, 56% of examined EYFP-negative tumors halved into HCC and benign lesions, including dysplasia (7%), RNs (17%) and HCA (23%) (Figure 5D), suggesting that these EYFP-negative tumors may derive from hepatocytes and/or Sox9-negative cells. Thus, HPCs contribute to liver tumorigeneity (Figure S5C).

In order to evaluate the aggressiveness of the lesions across different tumor types and to further classify HCC variants obtained from hepatocyte and HPC tracing, resected tumors were further analyzed by IHC, a “HAGCKS” (HSP70, AFP, GS, CK19, Ki67, and Sox9) score with arbitrary units was created to classify tumors aggressiveness, as previously reported (Gomes et al., 2016) (Supplemental Experimental Procedures; Figures S5D–S5V). Fundamentally, aggressive tumors irrespective of their origin have high HAGCKS scores. Expectedly, HCCs had the highest score, whereas HCAs, dysplasia, and RNs scored lower (Figures 5E and 5F). Notably, dysplasia and RNs had apparently a higher HAGCKS score than HCAs, suggesting that these lesions in URI mice are HCC precursors (Figure 5F). Histopathology indicated that HCCs emerging from hepatocytes were heterogeneous and resembled the spectrum of primary human HCC subtypes. Trabecular (41%), mixed (33%), glandular (19%), and solid HCCs (7%) emanated from hepatocyte tracing (Figures 5G). Only trabecular HCCs originated from HPCs (Figure 5G). Interestingly, solid HCCs had a higher HAGCKS score than other HCC variants (Figure 5H), suggesting that HCCs originating from hepatocytes may be slightly more aggressive than HCCs derived from HPCs. Therefore, in the URI model, which recapitulates several stages of human hepatocarcinogenesis, hepatocytes and HPCs contribute to liver tumor heterogeneity.

Furthermore, hepatocytes and HPCs were tracked in other hepatocarcinogenesis models described previously (Figure S5C) (Mu et al., 2015). Hepatocytes tracking in R26-mTOM-stop-mGFP mice infected with AAV8-Tgb^Cre^ and treated with DEN and CCl_4_ showed that hepatocytes were the HCC cell of origin (Figures 5E and S5C). This was confirmed in Mdr2 knockout (Mdr2^KO^) mice crossed with R26-stop-ZsGreen line and infected with AAV8-Tgb^Cre^ (Figures 5E and S5C). All tumors obtained in these mice were HCCs GFP positive from trabecular and mixed types (Figure S5W). However, HCAs or other lesions were not detected (Figures 5E and S5C).

Surprisingly, tracking of HPCs in Sox9^IRES-CreERT2^; R26-stop-EYFP mice treated with DEN or in CK19^IRES-CreERT2^; R26-mTOM-stop-mGFP mice treated with DEN and CCl_4_ revealed that all HCCs and HCAs obtained were EYFP or GFP negative (Figures 5F and S5C), suggesting that tumors do not originate from HPCs in these models and confirming that tamoxifen treatment of Sox9^IRES-CreERT2^; R26-stop-EYFP mice does not lead to Sox9 expression in hepatocytes (Carpentier et al., 2011). Thus, different liver cancer mouse models, possibly due to the type of damage (oncogene expression, location, and duration) and their capacity to mimic the human disease may have different impact on liver tumorigenesis.

### Hepatocytes Expressing Oncogenic URI Produce Galectin-3 to Instruct and Activate HPCs

The above findings suggest that transformed hepatocytes (e.g., oncogenic URI expression) may non-cell-autonomously instruct and determine the fate of HPCs toward oncogenesis. HPCs expand during tumorigenesis and when liver damage impairs hepatocyte turnover. hURI expression leads to NAD^+^-depletion-induced DNA damage, triggering hepatocyte damage and apoptosis via the p53-DNA damage response pathway (Tummala et al., 2014). Thus, relieving the p53-dependent proliferative break in hepatocytes would stop HPC expansion. In order to verify this, we inactivated p53 in hURI-tetOFF^hep^ mice by crossing them with p53ER^TAM^ mice, in which p53 activation requires ectopic 4-hydroxytamoxifen (Tummala et al., 2014). Surprisingly, while p53 was inactivated, IHC and WB showed that HPC expansion and increased Ki67 was independent of p53 (Figures S6A–S6E). p19ARF was also markedly expressed in hURI-tetOFF^hep^ liver sections (Tummala et al., 2014). However, p19ARF (*Cdkn2a*) deletion did not affect HPC expansion and increased Ki67 in hURI-tetOFF^hep^ mice (Figures S6A–S6D and S6F), suggesting that HPCs expand independently of hepatocytes’ proliferative status.

We next checked several inflammation-regulated factors described to induce oval cell expansion. mRNA levels of cytokines were not altered (Figure S6G). Additionally, levels of TWEAK (tumor necrosis factor-like weak inducer of apoptosis) did not change in early tumorigenesis stages (Figure S6H) (Jakubowski et al., 2005), and WNT/β catenin target genes were not upregulated in HPCs from hURI-tetOFF^hep^ and Sox9^IRES-EYFP^ crosses (Figure S6I). Finally, treating 3-week-old hURI-tetOFF^hep^ mice with anti-inflammatory sulindac did not abolish oval cell expansion (Figures S6J–S6L). Thus, inhibition of HPC expansion is independent of inflammatory cues in our model.

**Figure 6:**
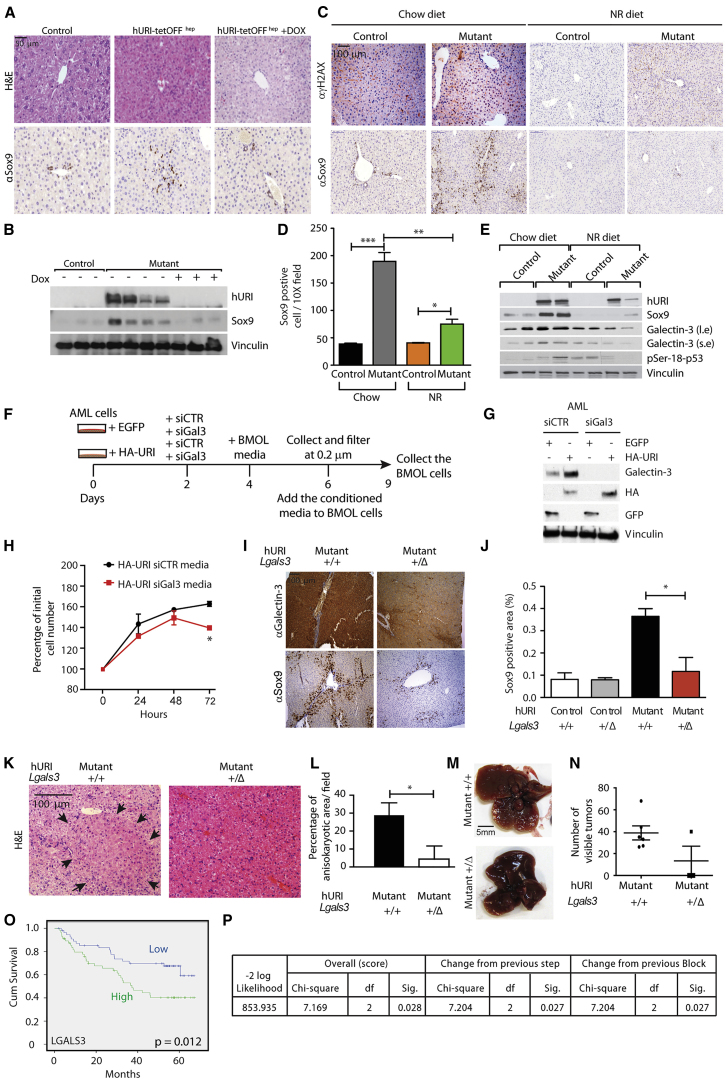
Hepatocytes Expressing Oncogenic URI Produce Galectin-3 to Instruct and Activate HPCs (A) Representative images of H&E and Sox9 staining in liver sections derived from 8-week-old hURI-tetOFF^hep^ mice fed with either chow or doxycycline (Dox) diet as previously described (Tummala et al., 2014). (B) WB of liver extracts from 8-week-old hURI-tetOFF^hep^ mice fed with either a chow or Dox diet as previously described (Tummala et al., 2014). Membranes were blotted with the indicated antibodies. (C) Representative images of γH2AX and Sox9 IHC in liver sections derived from 8-week-old hURI-tetOFF^hep^ mice fed with either chow or nicotinamide riboside (NR) diet (Tummala et al., 2014). (D) Quantification of Sox9 staining from (C) (n = 4). (E) WB of liver extracts from 8-week-old hURI-tetOFF^hep^ mice fed with either chow or NR diet. Membranes were blotted with the indicated antibodies. l.e., long exposure; s.e., short exposure. (F) Schematic representation of AML-12 and BMOL cell treatment. Media from EGFP- or HA-URI-overexpressing AML-12 cells transfected either with siCTR or siGal3 was used to culture BMOL cells for 3 days. (G) WB of AML-12 cells from (F). Membranes were blotted with the indicated antibodies. (H) Number of BMOL cells after 0, 24, 48, and 72 hr of incubation with media from AML-12 cells from (F). (I) Representative images of Sox9 and galectin-3 IHC in liver sections derived from 8-week-old mutant; Lgals3^+/+^ and mutant; Lgals3^+/Δ^ mice. (J) Quantification of Sox9 IHC from (I). n = 4 for control; Lgals3^+/+^ and mutant; Lgals3^+/+^ and n = 3 for control; Lgals3^+/Δ^ and for control; Lgals3^+/Δ^. (K) Representative images of H&E in liver sections derived from 10-week-old mutant(KI/KI); Lgals3^+/+^ and mutant(KI/KI); Lgals3^+/Δ^ mice. Black arrows denote anisokaryotic area. (L) Quantification of anisokaryotic area from (K) (n = 3). (M) Representative images of livers derived from 10-week-old mutant(KI/KI); Lgals3^+/+^ and mutant(KI/KI); Lgals3^+/Δ^ mice. (N) Quantification of number of visible tumors in livers from (M). n = 6 for mutant(KI/KI); Lgals3^+/+^ and n = 3 for mutant(KI/KI); Lgals3^+/Δ^. (O) Kaplan-Meier curve of human HCC patient cumulative survival based on the expression of *LGALS3.* Degrees of freedom = 1; chi-square = 6.243; p = 0.012. (P) Multivariate Cox regression survival for *LGALS3* and *URI1* in 221 patient human HCC gene expression analyses. (p = 0.027). “df” and “Sig.” represents degrees of freedom and significance, respectively. Data are presented as mean ± SEM. ^∗^p ≤ 0.05; ^∗∗^p ≤ 0.01; ^∗∗∗^p ≤ 0.001. Scale bars represent 5 mm, 100 μm, and 50 μm.

Switching off hURI expression with doxycycline significantly reduced HPC proliferation (Figures 6A and 6B), suggesting that continuous hURI expression is essential for oval cell expansion. Abrogation of DNA damage by supplying the NAD^+^ booster nicotinamide riboside (NR) (Tummala et al., 2014) reduced HPC proliferation in 8-week-old mice (Figures 6C–6E), suggesting that the NAD^+^-deficit-induced DNA damage axis is essential for oval cell expansion.

Previous iTRAQ analysis (Tummala et al., 2014) revealed that galectin-1 and galectin-3 were highly upregulated in 8-week-old hURI-expressing livers (Figure S6M). Galectins are extracellular β-galactoside-binding lectin, which bind to glycoproteins such as laminin and integrins (also expressed in mutant livers; Figures S2A, S2D, and S2E), to regulate and remodel the ECM and promote integrin signaling and fibrillogenesis, allowing HPC expansion during chronic liver injury (Hsieh et al., 2015). WB confirmed that galectin-3 was strikingly enhanced in 12-week-old mutant livers, but galectin-1 was only modestly increased (Figure S6N). WB and IF of 8-week-old hURI-tetOFF^hep^ livers confirmed that galectin-3 was upregulated in hepatocytes (Figures 6E and S6O). Abrogation of DNA damage by NR reduced galectin-3 levels (Figure 6E), suggesting that hepatocytic NAD^+^-deficit-induced DNA damage may be involved in galectin-3 secretion. Suppression of glycosylation-dependent surface glycoprotein by 4-fluoro-*N*-acetylgalactosamine (4-F-GalNAc), which inhibits the actions of galectin, significantly reduced HPC expansion in hURI-tetOFF^hep^ livers (Figures S6P–S6R). Thus, transformed hepatocytes are the source of galectin-3, which is essential for HPC expansion and tumorigenesis, highlighting a crosstalk between hepatocytes and HPCs.

To further test galectin-3-mediated activation of HPCs by transformed hepatocytes, mouse bipotential murine oval liver (BMOL) cells were cultured with conditioned media from AML-12 (alpha mouse liver 12) cells (nontumorigenic murine hepatocytes) stably expressing either EGFP or hemagglutinin (HA) URI. A concomitant knockdown of galectin-3 in AML-12 cells overexpressing HA-URI significantly reduced BMOL cell numbers (Figures 6F–6H). Similar results were obtained when hepatocytes were transformed by oncogenic c-MYC (data not shown), which is known to be implicated in hepatocarcinogenesis (Burén et al., 2016), suggesting that transformed hepatocytes, through DNA damage, talk to HPCs via galectin-3 secretion.

Next, hURI-tetOFF^hep^ mice crossed with heterozygous galectin-3 knockout mice had significantly reduced HPCs and dysplastic lesions and number of hepatic tumors (Figures 6I–6N). Notably, levels of galectin-3 were significantly much higher in the URI model than in DEN-treated mice (Figures S6S and S6T). Galectin-3 is thus determinant for HPC expansion and their tumorigenic fate in the hURI-tetOFF^hep^, but not in DEN-treated mice.

To estimate the clinical relevance of our findings, we interrogated the galectin family gene signature using gene expression dataset from a cohort of 221 HCC patients (GEO: GSE14520) (Roessler et al., 2010). Multivariate Cox regression indicated that the overall survival of HCC patients was significantly associated with *LGALS3*, *LGALS4*, and *LGALS7* gene signatures (p = 0.025) (Figure S6U), but not with all combined *LGALS* genes (data not shown). Importantly, *LGALS3* gene expression positively correlated with patient poor prognosis (Figure 6O), whereas *LGALS4* and *LGALS7* gene expression did not show significant differences (Figures S6V and S6W). Furthermore, the multivariate Cox regression suggested a significant association between *URI* (*C19orf2*) or *c-MYC* and *LGALS3* expression and predicted poor patient survival (p = 0.027 for *URI* and p = 0.023 for *c-MYC*) (Figures 6P and S6X). Thus, galectin-3 activates HPC, negatively affecting overall patient survival.

### Hepatocytes Expressing Oncogenic URI Produce α-Ketoglutarate and Galectin-3 to Preserve an HPC Undifferentiated State

**Figure 7:**
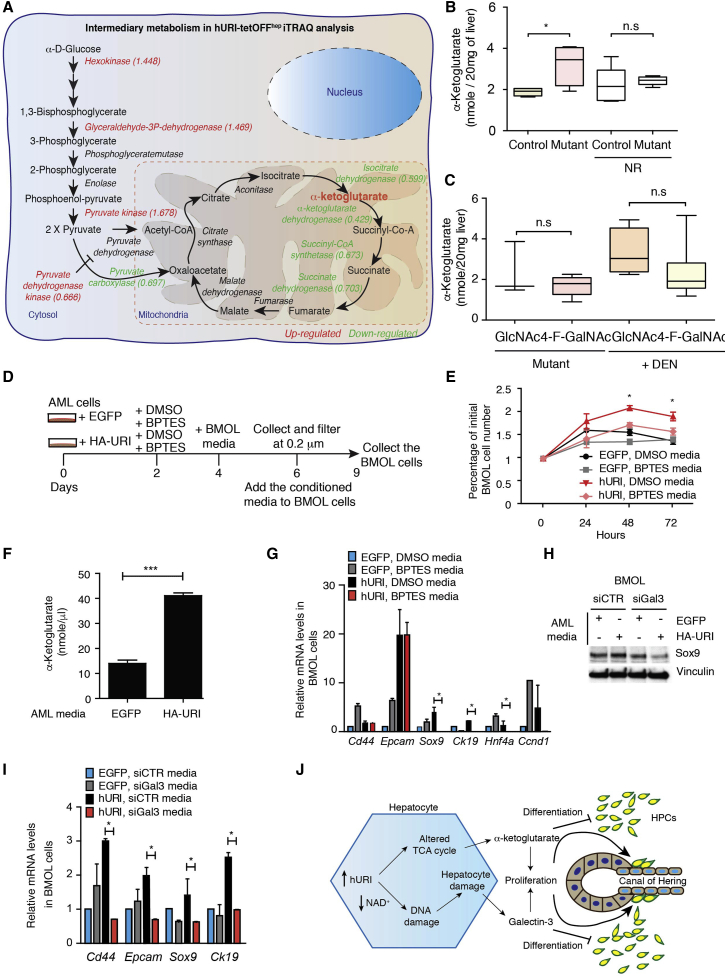
Hepatocytes Expressing Oncogenic URI Produce α-Ketoglutarate and Galectin-3 to Preserve an HPC Undifferentiated State (A) Schematic representation of intermediary metabolism induced in hURI-tetOFF^hep^ mice. Data were obtained and analyzed from previous iTRAQ data obtained from livers from 1- or 8-week-old hURI-tetOFF^hep^ mice (Tummala et al., 2014). Fold changes are represented within the brackets. Upregulated enzymes are represented in red, and downregulated enzymes are represented in green. (B) Histogram representing α-ketoglutarate levels from livers from either NR-treated or untreated 12-week-old hURI-tetOFF^hep^ mice as indicated. n = 6 for controls and mutants; n = 5 for controls and mutants treated with NR. (C) Histogram representing α-ketoglutarate levels from livers from 4-week-old hURI-tetOFF^hep^ mice (mutant) and DEN-treated C57BL/6 mice. Mice were treated with either GlcNAc or 4-F-GalNAc for 3 weeks. n = 3 for mutants treated with GlcNAc. (D) Schematic representation of AML-12 and BMOL cell treatment. Media from HA-URI-overexpressing AML-12 cells treated either with DMSO or BPTES (10 μM) was used to culture BMOL cells for 3 days. (E) Cell number of BMOL cells after 0, 24, 48, and 72 hr of incubation with media from AML-12 cells from (D). (F) Histogram representing α-ketoglutarate levels in media from AML-12 cells stably overexpressing EGFP or HA-URI. (G) qRT-PCR of BMOL cells cultured with media from AML-12 cells as described in (D). (H) WB of BMOL cells after 72 hr incubation with media from AML-12 cells stably overexpressing EGFP or HA-URI and transfected with either siCTR or siGal3. Membrane was blotted with the indicated antibodies. (I) qRT-PCR of BMOL cells cultured with media from AML-12 cells as described in (H). (J) Scheme representing hepatocytes expressing oncogenic URI-secreted galectin-3 to induce HPC proliferation, whereas α-ketoglutarate preserves an HPC undifferentiated state during expansion. Data are presented as mean ± SEM. ns, non significant; ^∗^p ≤ 0.05; ^∗∗∗^p ≤ 0.001.

To probe signaling changes that may explain why HPCs do not immediately differentiate into hepatocytes while proliferating, we further analyzed our previously reported iTRAQ data (Tummala et al., 2014). Several glycolysis enzymes, including hexokinase (HK1), glyceraldehyde-3-phosphate dehydrogenase, and pyruvate kinase, were upregulated in early tumorigenesis in hURI-tetOFF^hep^ hepatocytes (Figures 7A and S7A), suggesting a pseudo-Warburg-like effect. Accordingly, pyruvate dehydrogenase kinase, which inactivates pyruvate dehydrogenase (catalyzing pyruvate to acetyl-coenzyme A conversion), was downregulated (Figures 7A and S7A). Many other enzymes involved in the tricarboxylic acid (TCA) cycle acting downstream of α-ketoglutarate were also downregulated (Figures 7A and S7A). Thus, glycolytic flux may result in α-ketoglutarate accumulation, maintaining the undifferentiated state of HPCs in the liver (Carey et al., 2015, Saha et al., 2014).

In accordance with our iTRAQ data, α-ketoglutarate was significantly elevated in mutant livers (Figure 7B) but decreased following NR treatment, suggesting that hepatocytic NAD^+^-depletion-induced DNA damage is essential for α-ketoglutarate elevation (Figure 7B). Further, 4-F-GalNAc treatment did not affect the α-ketoglutarate levels (Figure 7C), suggesting that α-ketoglutarate accumulation is independent of changes in glycan structures. Treatment of BMOL cells with dimethyl 2-oxoglutarate (or dimethyl-α-ketoglutarate), a precursor for the glutamine synthesis, significantly increased expression of stem cell markers such as *Cd44, Sox9*, or *Epcam*, but expression of *Hnf4a* was reduced, as shown by qRT-PCR (Figure S7B). This suggests that increased α-ketoglutarate maintains HPC undifferentiated state. Addition of dimethyl succinate and dimethyl 2-oxoglutarate further enhanced the expression of stem cells markers (Figure S7B), while dimethyl succinate favored an intermediate state, confirming previous work and showing that the α-ketoglutarate/succinate ratio suppresses stem cell differentiation (Carey et al., 2015). Additionally, media from AML-12 cells stably expressing HA-URI and treated with bis-2-(5-phenylacetamido-1,3,4-thiadiazol-2-yl)ethyl sulfide (BPTES), a selective inhibitor of glutaminase GLS1 reducing α-ketoglutarate levels, decreased BMOL cell numbers (Figures 7D and 7E) (Xiang et al., 2015). Notably, media from HA-URI-expressing AML-12 cells had higher levels of α-ketoglutarate (Figure 7F). However, in vitro, only few stem cell markers were reduced (Figure 7G), suggesting that other paracrine signals (e.g., galectin-3) emerging from transformed hepatocytes contribute to preserve the HPC stemness.

Multivariate Cox regression of a gene set of intermediate metabolism downregulated in URI model revealed a nonsignificant association between expression of *IDH1*, *IDH2*, *IDH3A*, *IDH3B*, *IDH3G*, *OGDH*, *SCS*, *SDH*, or *PC3* and overall patient survival (Figure S7C). Expression analysis of a single gene from intermediate metabolism also failed to classify the patients with poor prognosis (data not shown). We therefore checked whether galectin-3 was sufficient to sustain the undifferentiated state of HPCs. BMOL cells cultured with media from AML-12 cells expressing HA-URI or His-c-MYC and depleted for galectin-3 showed a significant reduction of all stem cell markers (Figures 7H, 7I, and S7D–S7G). Galectin-3 secreted by transformed hepatocytes may thus play a prominent role in maintaining HPC stemness, expansion, and aggressiveness (Figure 7J).

## Discussion

Different murine models, possibly due to their capacity of mimicking the human disease or the type of damage (oncogene expression, location, and duration) impact differently on tumorigenesis. In the hURI-tetOFF^hep^ mouse model, which recapitulates some features and stages of the human hepatocarcinogenesis (Gomes et al., 2016, Tummala et al., 2014), hepatocytes and HPCs contribute to liver tumor heterogeneity. Supporting this, transplanted HPCs from normal mice regenerate hepatocytes and restore liver parenchyma in mice with hepatocytic deletion of the E3 ubiquitin ligase Mdm2-induced apoptosis, necrosis, and foremost senescence (Lu et al., 2015). Consistently, in the hURI-tetOFF^hep^ model, HPCs clearly supply new mature hepatocytes, even though we do not completely exclude that hepatocytes can also derive from pre-existing hepatocytes (Schaub et al., 2014, Yanger et al., 2014). Interestingly, hepatocytes expressing URI show high levels of replicative stress and undergo into senescence (Tummala et al., 2014), arguing that the type of hepatocyte damage may determine HPC fate during tumorigenesis. HPCs are therefore a potential future alternative to hepatocyte or liver transplantation for liver disease, depending on the damage, and can participate in hepatocarcinogenesis.

Proliferation block or apoptosis of hepatocytes triggers growth of HPCs (Jung et al., 2010). In our study, HPC proliferation seems to depend on the hepatocyte’s oncogenic status rather than its proliferative capacity. Transformed hepatocytes instruct the neighboring HPCs through paracrine signal mediated by NAD^+^-deficit-induced DNA damage, leading to galectin-3 and α-ketoglutarate secretion, to maintain an HPC undifferentiated state and activation. In this regard, galectins are implicated in fibrosis of multiple organs. Specifically, galectin-3 is reportedly activated in cirrhotic liver and HCC (Hsu et al., 1999), and high circulating levels are associated with poor prognosis in patients with primary HCC, corroborating our results (Jiang et al., 2014).

Activated HPCs contribute to the formation of RNs, HCAs, and HCCs. Notably, shared mutations by HPCs and RNs putatively indicate common origins (Lin et al., 2010). Moreover, RNs mainly occur in cirrhotic livers and define histological development of cirrhosis, a potent risk factor for HCC (Lin et al., 2010, Schuppan and Afdhal, 2008). RNs are thus likely HCC precursors. Thus, HPC activation may engender cirrhosis and subsequently, through selective pressure, HCC formation.

HCA is a rare benign liver tumor primarily found in women using hormonal contraceptives. The etiology is uncertain, but direct linkage between estrogen exposure and HCA growth is suspected, and estrogens reportedly contribute to the proliferative responses of biliary epithelial cells (Fouassier et al., 2009). Interestingly, HCC may result from malignant transformation of HCA (Pilati et al., 2014), even though this transformation does not occur in non-fibrotic livers (Schulze et al., 2015). Finally, patients with trabecular HCCs often have NASH (Salomao et al., 2010). Accordingly, hURI-tetOFF^hep^ mice develop NASH and trabecular HCCs (Gomes et al., 2016, Tummala et al., 2014).

Transplantation of DC-induced organoids has been recently proposed to promote liver regeneration, especially for patients with resected HCC (Huch et al., 2013a). Since HPCs give rise to RN-induced cirrhosis, the re-implantation strategy of HPCs from HCC patients should be approached cautiously. Liver transplantation remains the only curative option for some patients, but pharmacological blockage of galectin-3 may offer elegant approaches for inhibiting cirrhosis progression and HCC, thereby reducing needs for transplantation. Finally, HCC is an intractable primary liver cancer, certainly due to the recurrence of multiple secondary liver tumors after surgical interventions or first therapies. HPCs predict recurrence in HCC patients after curative resection (Ye et al., 2014). Our findings may thus provide a disease management strategy after primary surgical resection to circumvent tumor recurrence, facilitating the design of efficient therapies against liver cancer and recurrences.

## Experimental Procedures

### Antibodies

Materials and antibodies were purchased from different manufacturers as detailed in Supplemental Experimental Procedures.

### Generation, Handling, and Treatment of Mice

Mice were generated, handled, and treated as previously described (Gomes et al., 2016, Tummala et al., 2014) and detailed in Supplemental Experimental Procedures. All mice have been housed in pathogen-free conditions. All experiments were approved by the CNIO-ISCIII Ethics Committee and performed in accordance with the guidelines for ethical conduct in the care and use of animals as stated in the international guiding principles for biomedical research involving animals, developed by the Council for International Organizations of Medical Sciences. Littermates were always used as controls. No gender differences were observed and age/developmental stage of mice was included appropriately in the text and the figure legends. Food (Harlan Laboratories and Research Diets Inc.) and water were provided ad libitum.

### Cell Culture, Transfection, and Cell Number Counts

Cell culture, transfection, and cell number counts were performed as detailed in Supplemental Experimental Procedures.

### Image Analysis

Six images per slide were taken at 10× and 20× magnifications and quantified using color de-convolution, a colocalization finder, and image analysis tools available in Fiji/ImageJ software (http://fiji.sc/).

### HAGCKS Score

Consecutive liver sections were stained by immunohistochemistry for HSP70, AFP, GS, CK19, Ki67, and Sox9 (HAGCKS). Differential expression levels (0–3) of GS and HSP70 as well as Sox9 were evaluated using previously reported classifications (Gomes et al., 2016). CK19 and AFP were only classified in positive (1) or negative (0) cases. Individual cases were considered immunoreactive (IR) for individual antigens when more than 5% of cells were IR. IR cases were further sub-classified as follows: 5%–10% IR cells (1), 11%–50% IR cells (2), and more than 50% IR cells (3). HAGCKS score was determined by adding the grading of immunohistochemistry signal intensity for each individual marker and scored in Figure 5.

### α-Ketoglutarate Measurement

Total α-ketoglutarate levels were measured according to the manufacturer’s instruction provided by a kit purchased from Sigma-Aldrich (MAK054A). For liver tissue, the protocol was scaled up to two times.

### Serum TWEAK Measurement

Serum TWEAK levels were measured according to the manufacturer’s instructions using a Mouse TWEAK/TNFSF12 ELISA kit purchased from R&D Systems (DY1237).

### Flow Cytometry of HPCs

Flow cytometry of HPCs was performed as detailed in Supplemental Experimental Procedures.

### Immunoblotting

Immunoblotting was performed as previously described elsewhere (Tummala et al., 2014).

### Immunohistochemistry and Immunofluorescence

Immunohistochemistry and immunofluorescence were performed as previously described (Tummala et al., 2014).

### Genotyping and qRT-PCR

Genotyping and qRT-PCR (including primer sequences) were performed as detailed in Supplemental Experimental Procedures.

### Statistical Analysis

Statistical analyses were performed using GraphPad Prism V5.0 software. Statistical significance (^∗^p ≤ 0.05, ^∗∗^p ≤ 0.01, and ^∗∗∗^p ≤ 0.001) between the means of a minimum of three groups was determined using unpaired two-tailed Student’s t test or linear regression analysis. Results are expressed as the mean value ± SEM or SD, as indicated. All results, including WB, are representative of at least three independent experiments. Statistical parameter false discovery rate estimates the probability of a gene set with false-positive finding. Normalized enrichment score allows to compare enrichment analysis results across gene sets. Tumor numbers normalization in the Sox9^IRES-CreERT2^ model was done according to Cre recombination efficiency, and contingency analysis was performed using two-sided Fischer’s exact tests (results are displayed in Figures S5A and S5B). Survival and Kaplan-Meier analyses for individual genes were performed using publically available human HCC patient gene expression datasets (GEO: GSE14520) (Roessler et al., 2010). Multivariate Cox regression analyses were performed using SPSS software (version 19).

## Author Contributions

K.S.T. designed performed and analyzed most of the in vivo experiments. M.B. designed performed and analyzed in vitro experiments and experiments with the Gal3KO mice. K.S.T., M.B., A.T., and C.P. performed and analyzed HAGCKS scores. C.P. analyzed all tissues histology. O.G. performed the bioinformatics analyses. R.F.S. provided some paraffin-embedded blocks used in HAGCKS scoring and previously reported (Mu et al., 2015). K.S.T., M.B., and N.D. analyzed all the data. N.D. designed the experiments and conceived, developed, and wrote the project and the manuscript. Funding was secured by N.D.

## Accession Numbers

The accession number for the FACS data reported in this paper is Flow Repository: FR-FCM-ZY3T.

## Supplemental Information

Document S1. Supplemental Experimental Procedures and Figures S1–S7

Document S2. Article plus Supplemental Information
