# Supplementary material for: Hepatocellular Carcinomas Originate Predominantly from Hepatocytes and Benign Lesions from Hepatic Progenitor Cells
Source: Cell Rep. 2017 Apr 19;19(3):584–600. doi: 10.1016/j.celrep.2017.03.059 (PMC5409928; doi:10.1016/j.celrep.2017.03.059)
Supplement: Document S1. Supplemental Experimental Procedures and Figures S1–S7 [file mmc1.pdf]

**Cell Reports, Volume *19***

## Supplemental Information

# Hepatocellular Carcinomas Originate Predominantly from Hepatocytes and Benign Lesions from Hepatic Progenitor Cells

**Krishna S. Tummala, Marta Brandt, Ana Teijeiro, Osvaldo Graña, Robert F. Schwabe, Cristian Perna, and Nabil Djouder**

## SUPPLEMENTAL INFORMATION

### Supplemental Data

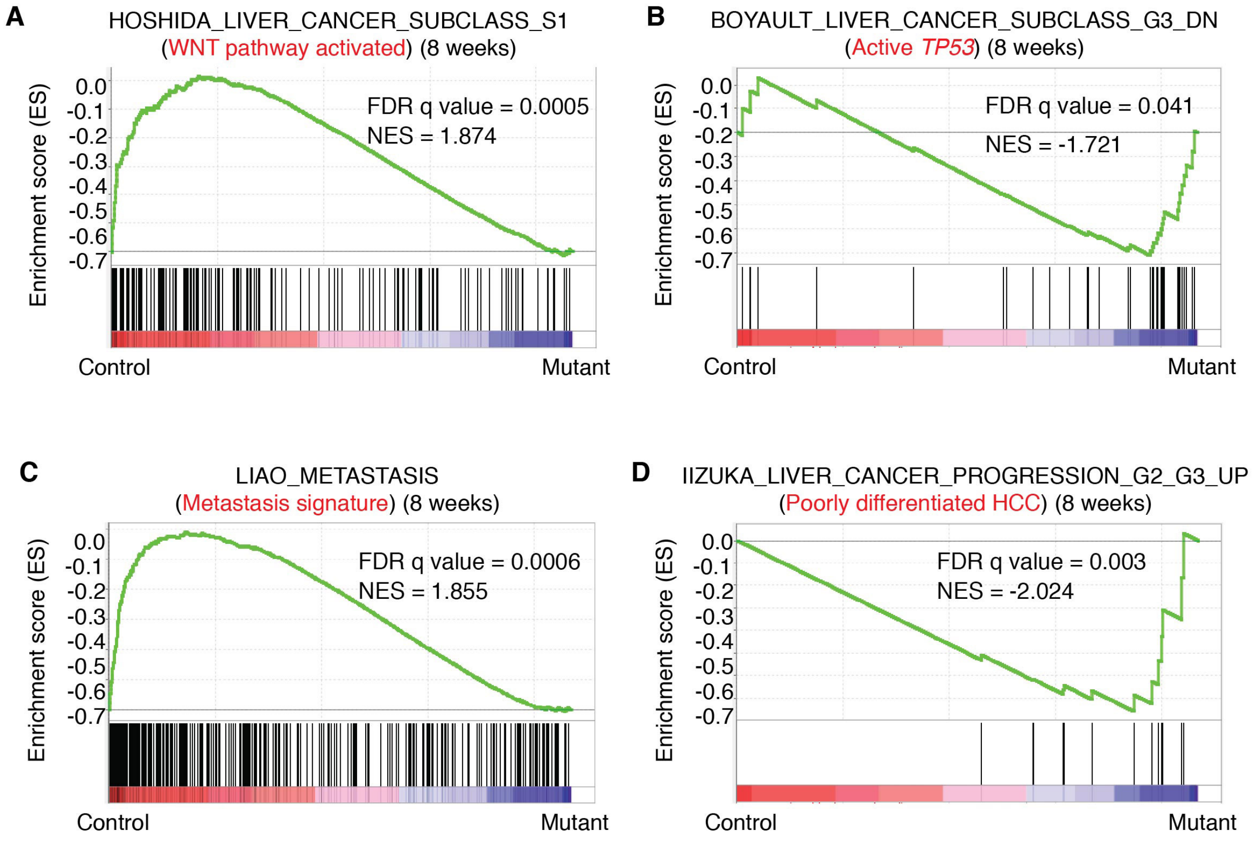


**Figure S1. hURI-tetOFF$^{hep}$ Mice Display HPC Signatures in Early Hepatocarcinogenesis Stages Correlating with Aggressive Human HCC, Related to Figure 1**

(A-D) GSEA of human HCC gene sets and 8-week-old hURI-tetOFF$^{hep}$ mice data sets previously analyzed (Tummala et al., 2014).

Normalized enrichment scores (NES) and false discovery rates (FDR) q-value are indicated in each graph.

**A**

| S.No | Protein name | Symbol | Fold change | | Rank | |
|---|---|---|---|---|---|---|
| | | | 1 week | 8 weeks | 1 week | 8 weeks |
| 1 | Keratin, type1 cytoskeletal 19 | Krt19 | 1.058 | 6.567 | 647 | 1 |
| 2 | Transgelin-2 | Tagln2 | 0.810 | 2.599 | 2262 | 9 |
| 3 | Creatine kinase B-type | Ckb | 1.007 | 2.377 | 1179 | 14 |
| 4 | Transgelin | Tagln | 0.818 | 2.107 | 2262 | 29 |
| 5 | Vimentin | Vim | 1.016 | 2.022 | 1078 | 38 |
| 6 | Translationally-controlled tumor protein | Tpt | 1.306 | 1.873 | 38 | 54 |
| 7 | Laminin subunit gamma-1 | Lamc1 | 1.133 | 1.767 | 249 | 67 |
| 8 | Annexin A3 | Anxa3 | 1.060 | 1.650 | 629 | 87 |
| 9 | Keratin, type II cytoskeletal 7 OS | Krt7 | 1.218 | 1.608 | 87 | 96 |
| 10 | Heterogeneous nuclear ribonucleoprotein A1 | Hnmpa1 | 0.985 | 1.474 | 1048 | 137 |
| 11 | Heterogeneous nuclear ribonucleoprotein A/B | Hnmpab | 1.029 | 1.353 | 932 | 208 |

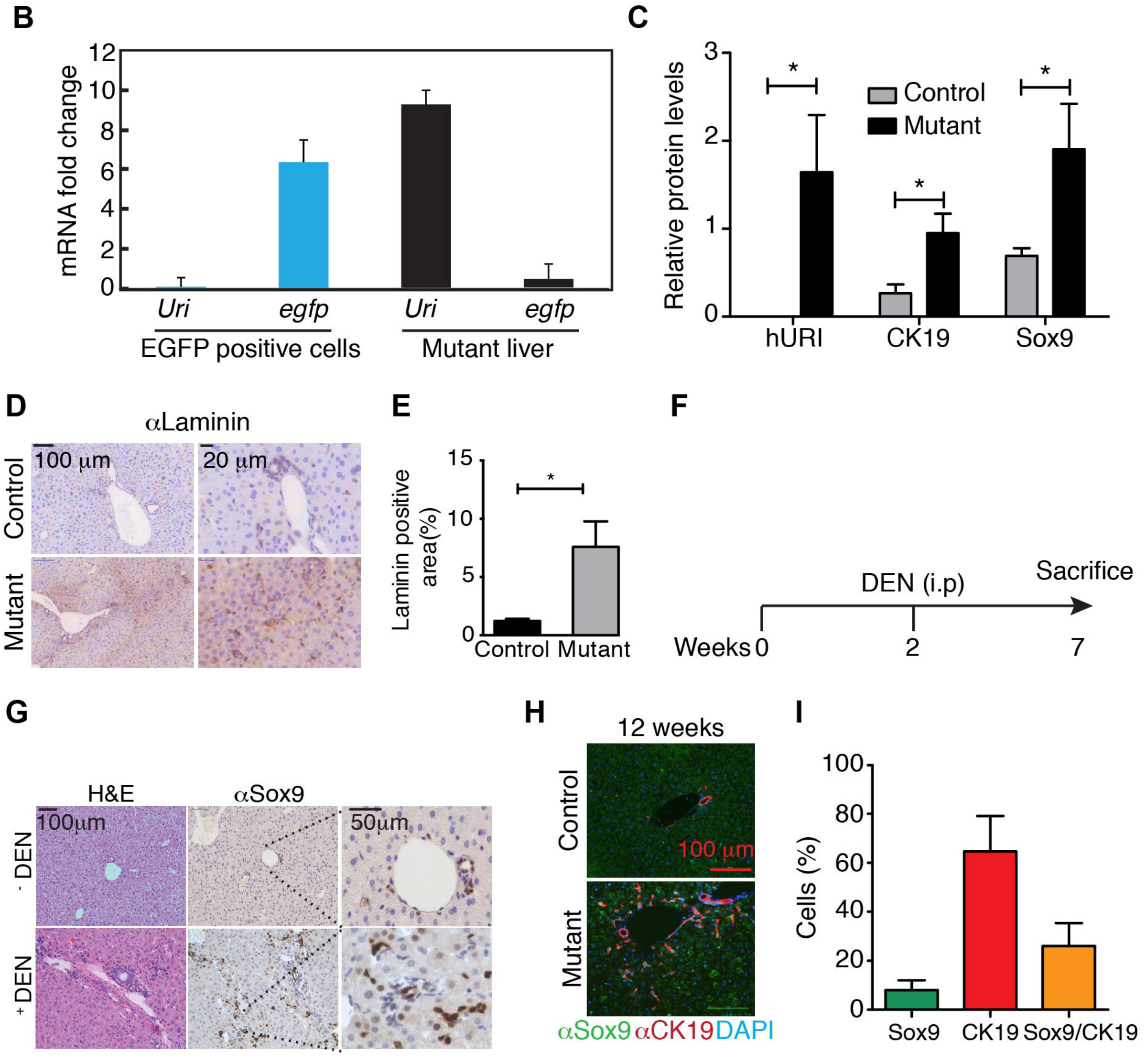

**Figure S2. HPCs Expand in the Early Stages of Hepatocarcinogenesis, Related to Figure 2**

(A) iTRAQ data from 1- and 8-week old hURI-tetOFF$^{hep}$ previously described (Tummala et al., 2014). Fold change represents the ratio of protein levels between control and mutant livers. Rank represents the ranking position of differentially expressed proteins.

(B) qRT-PCR analysis of FACS-sorted Sox9/EGFP-positive cells from 12-week-old hURI-tetOFF$^{hep}$ mice crossed with Sox9$^{IRES\text{-}EGFP}$ line. Error bars represent ± SD.

(C) Quantification of WB represented in Figure 2C.

(D) IHC analysis of laminin in liver sections from 12-week-old hURI-tetOFF$^{hep}$ mice.

(E) Quantification of (D).

(F) Schematic representation of diethylnitrosamine (DEN)-treated C57BL/6 mice. Mice were injected intraperitoneally with one single dose of 25 mg/kg of DEN at 2 weeks of age and were sacrificed at 7 weeks of age.

(G) Representative pictures of H&E staining and Sox9 IHC of liver sections derived from 7-week-old C57BL/6 mice treated as described in (F).

(H) Co-immunofluorescence of Sox9 and CK19 in liver sections from 12-week-old hURI-tetOFF$^{hep}$ mice.

(I) Quantification of (H).

Unless otherwise indicated data represented as mean ± SEM.

Scale bar represents 20 μm, 50 μm and 100 μm. $p \leq 0.05$ = *

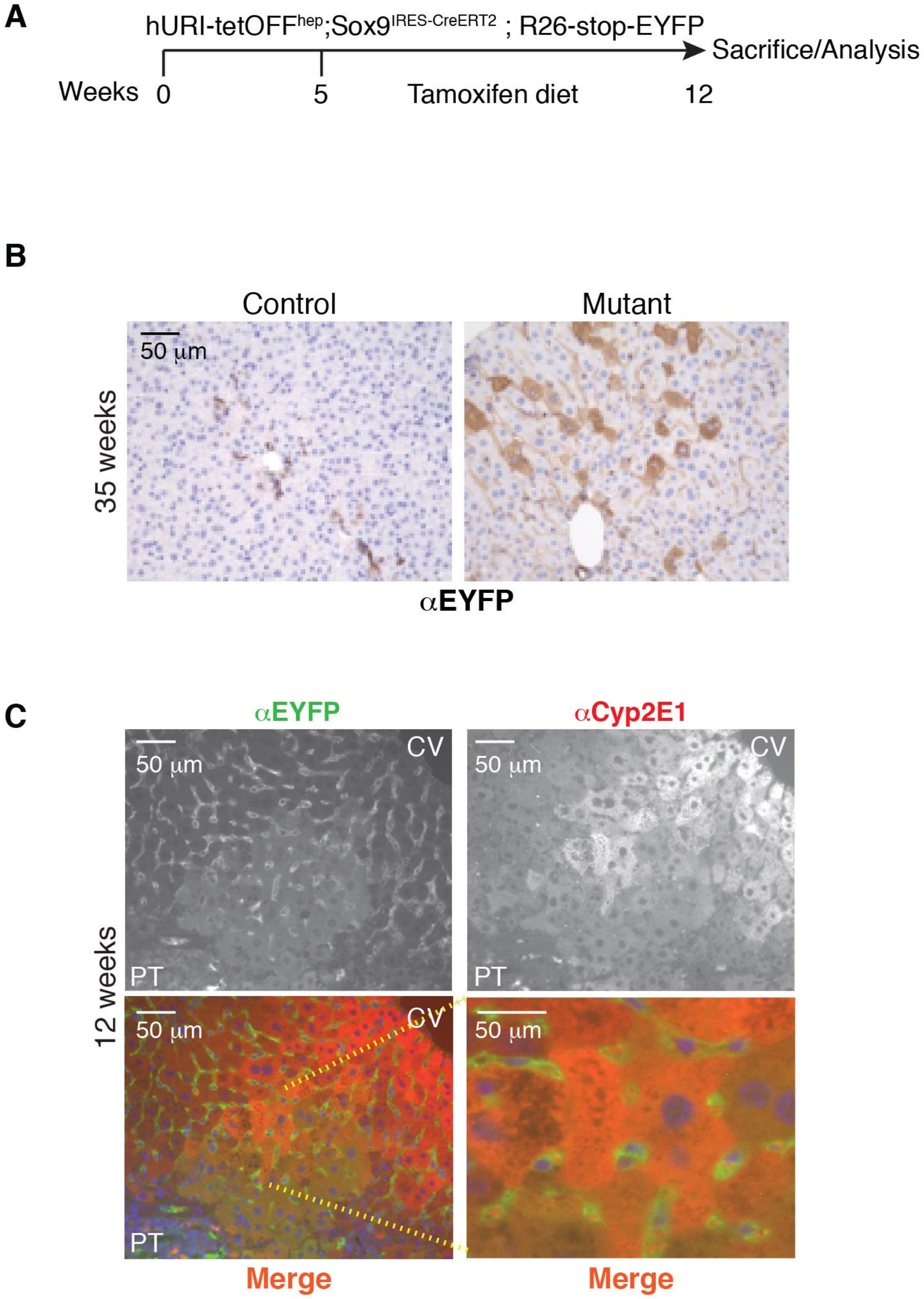


**Figure S3. HPCs Contribute to Liver tumorigenesis, Related to Figure 3**

(A) Scheme representing tamoxifen-treated hURI-tetOFF$^{hep}$ ; Sox9$^{IRES\text{-}creERT2}$ ; R26-stop-EYFP mice.

(B) Representative images of EYFP stained by IHC in liver sections from 35-week-old hURI-tetOFF$^{hep}$; Sox9$^{IRES\text{-}creERT2}$; R26-stop-EYFP mice.

(C) Co-immunofluorescence of EYFP and Cyp2E1 from mice described in (B).

Scale bars represent 50 μm.

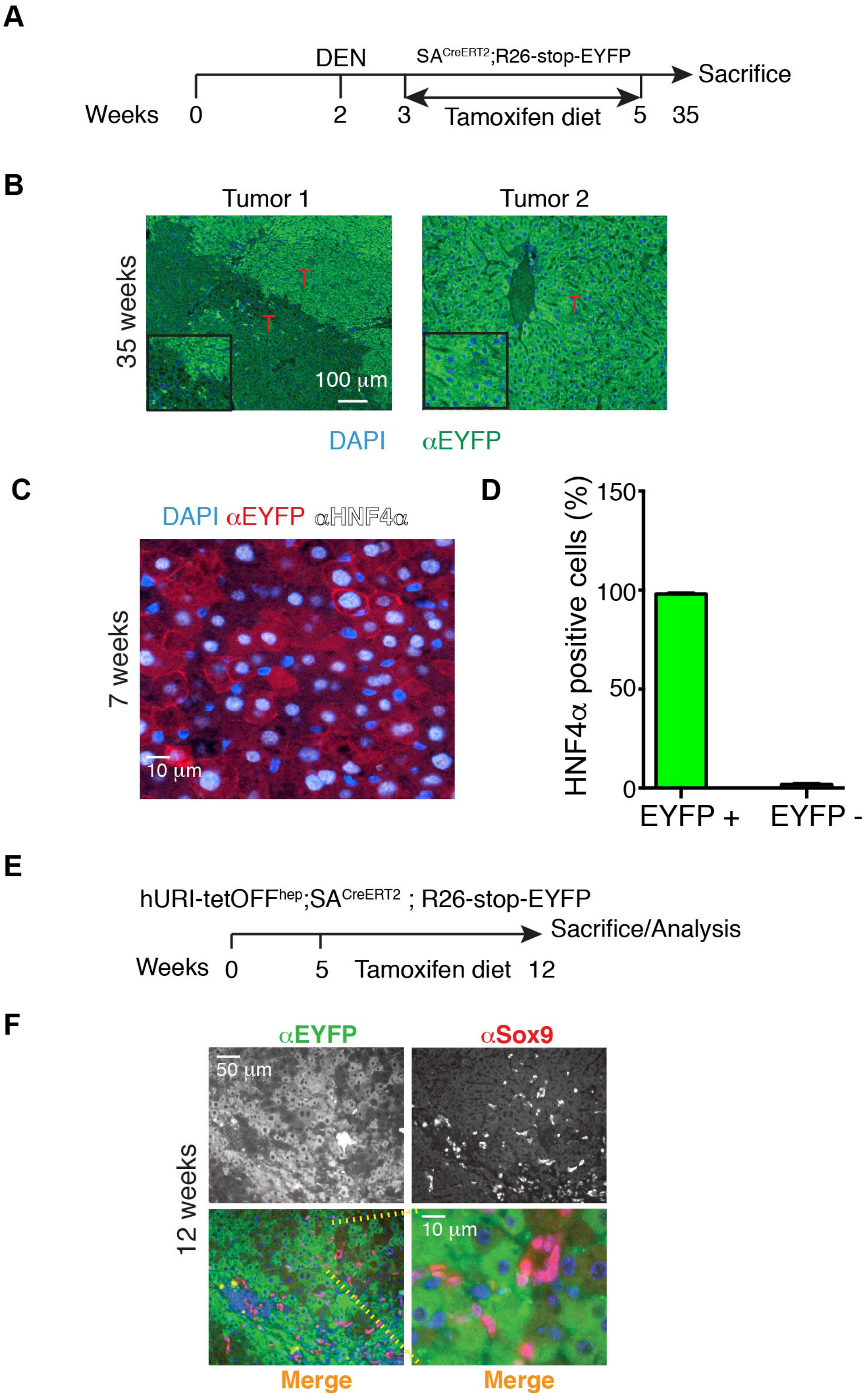
A
DEN
SA^CreERT2;R26-stop-EYFP
Sacrifice
Weeks 0 2 3 Tamoxifen diet 5 35
B
Tumor 1
Tumor 2
35 weeks
T
100 μm
DAPI αEYFP
C
DAPI αEYFP αHNF4α
7 weeks
10 μm
D
HNF4α positive cells (%)
150
100
50
0
EYFP +
EYFP -
E
hURI-tetOFF^hep;SA^CreERT2 ; R26-stop-EYFP
Sacrifice/Analysis
Weeks 0 5 Tamoxifen diet 12
F
αEYFP
αSox9
50 μm
12 weeks
10 μm
Merge
Merge

**Figure S4. Hepatocytes Contribute to Liver Tumorigenesis, Related to Figure 4**

(A) Schematic representation of DEN- and tamoxifen-treated 35-week-old $SA^{CreERT2}$ ; R26-stop-EYFP mice.

(B) Representative immunofluoresence images of EYFP staining in tumors obtained from DEN-treated $SA^{CreERT2}$ ; R26-stop-EYFP mice as described in (A). n = 10 tumors.

(C) Co-immunofluoresence analysis of HNF4α and EYFP in liver sections from DEN- and tamoxifen-treated 7-week old $SA^{CreERT2}$ ; R26-stop-EYFP mice. n = 3.

(D) Quantification of HNF4α and EYFP co-localisation from (C). n = 3.

(E) Schematic representation of tamoxifen treatment in hURI-$tetOFF^{hep}$ ; $SA^{CreERT2}$ ; R26-stop-EYFP mice.

(F) Co-immunofluoresence analysis of Sox9 and EYFP in liver sections from 12-week-old hURI-$tetOFF^{hep}$ mice. n = 5.

Scale bars represent 10 μm, 50 μm and 100 μm

A

| Liver pathology | | HCC | | HCA | | Dysplasia + Regeneration | |
|---|---|---|---|---|---|---|---|
| Normalized | EYFP | Negative | Positive | Negative | Positive | Negative | Positive |
| | SA $^{CreERT2}$ (to 99.95%) | 10 | 27 | 10 | 12 | 3 | 0 |
| | Sox9 $^{IRESCreERT2}$ (to 44.2%) | 16 | 5 | 7 | 5 | 7 | 25 |

B

| Normalized | P value (Fisher's test) | P value (Summary) | Relative Risk | Reciprocal Relative Risk | Odds ratio | Reciprocal Odds ratio |
|---|---|---|---|---|---|---|
| HCC | 0.0004 | *** | 0.3547 | 2.819 | 0.1157 | 8.64 |
| HCA | 0.7207 | ns | 0.7792 | 1.283 | 0.5952 | 1.68 |
| Dysplasia + Regeneration | 0.0183 | * | 4.571 | 0.2188 | ~ | 0 |

C

| Cell type tracked | Model of tumorigenesis | Tracking system | Number of tumors |
|---|---|---|---|
| HPC | hURI-tetOFF$^{hep}$ | Sox9$^{IRES-creERT2}$ ; R26-stop-EYFP | 65 |
| | DEN | Sox9$^{IRES-creERT2}$ ; R26-stop-EYFP | 6 |
| | DEN and $CCl_4$ | CK19$^{IRES-CreERT2}$; R26-mTOM-stop-mGFP | 8 |
| Hepatocyte | hURI-tetOFF$^{hep}$ | SA$^{CreERT2}$; R26-stop-EYFP | 59 |
| | DEN | SA$^{CreERT2}$; R26-stop-EYFP | 10 |
| | DEN and CCl4 | AAV8-Tgb$^{Cre}$ ; R26-mTOM-stop-mGFP | 42 |
| | Mdr2$^{KO}$ | AAV8-Tgb$^{Cre}$ ; R26-stop-ZsGreen | 7 |

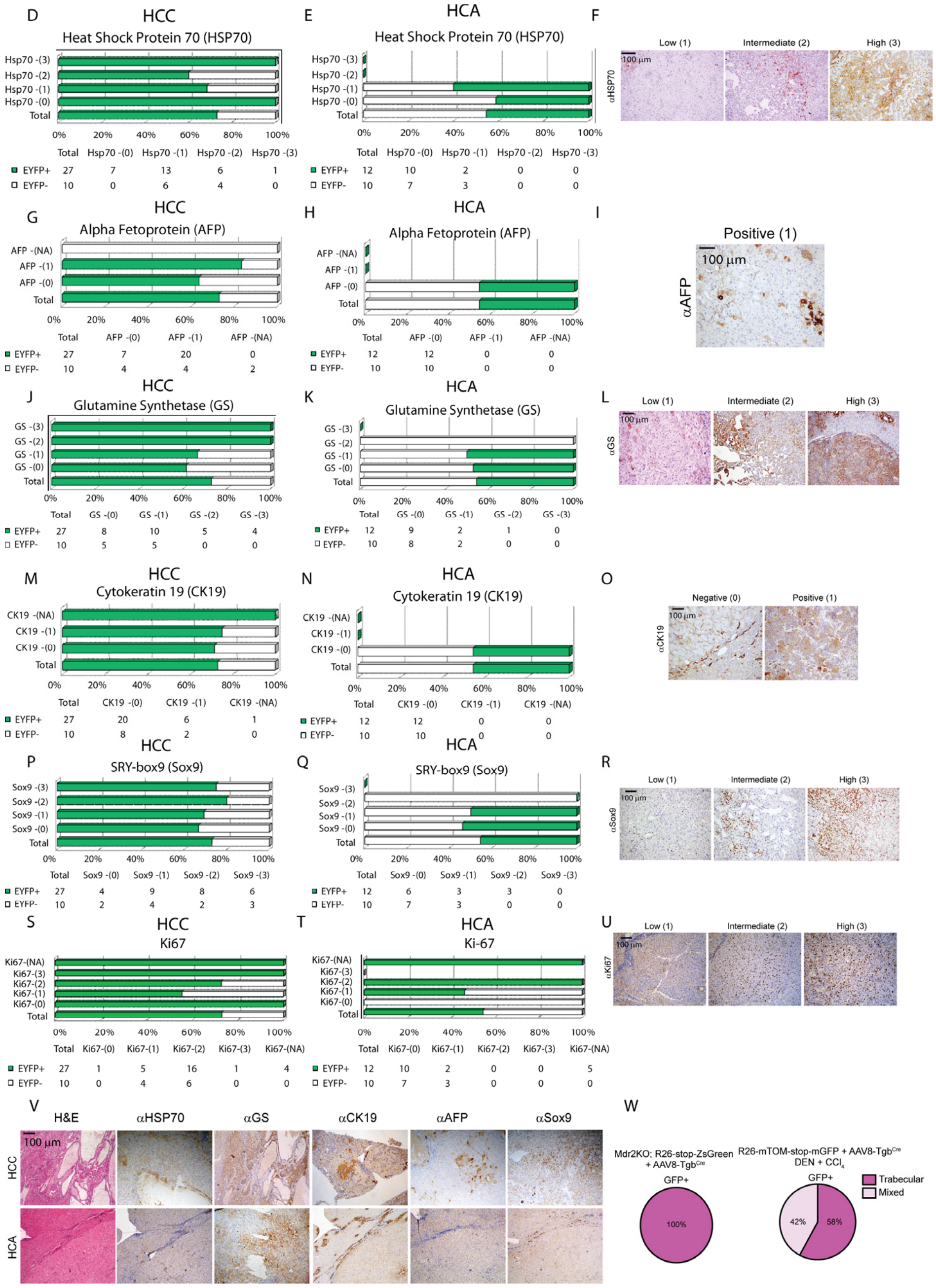

**Figure S5. HCC Predominantly Originates From Hepatocytes and HPCs Contribute to Liver Tumor Heterogeneity, Related to Figure 5**

(A) Normalized number of EGFP positive and negative dysplastic and regenerative nodules, HCA and HCC derived from hURI-tetOFF$^{hep}$ crossed with SA$^{CreERT2}$ ; R26-stop-EYFP and hURI-tetOFF$^{hep}$ crossed with Sox9$^{CreERT2}$ ; R26-stop-EYFP.

(B) Statistical analysis of normalized number of tumors from (A).

(C) Table summarizing all tumors obtained from different carcinogenesis models.

(D) Histogram representing HSP70 classification used to determine HAGCKS score in HCC derived from hURI-tetOFF$^{hep}$ ; SA$^{CreERT2}$ ; R26-stop-EYFP mice.

(E) Histogram representing HSP70 classification used to determine HAGCKS score in HCA derived from hURI-tetOFF$^{hep}$ ; SA$^{CreERT2}$ ; R26-stop-EYFP mice.

(F) Representative images of HSP70 IHC showing staining classified as low (1), intermediate (2) and high (3).

(G) Histogram representing AFP classification used to determine HAGCKS score in HCC derived from hURI-tetOFF$^{hep}$ ; SA$^{CreERT2}$ ; R26-stop-EYFP mice.

(H) Histogram representing AFP classification used to determine HAGCKS score in HCA derived from hURI-tetOFF$^{hep}$ ; SA$^{CreERT2}$ ; R26-stop-EYFP mice.

(I) Representative images of AFP IHC showing staining classified as positive (1).

(J) Histogram representing GS classification used to determine HAGCKS score in HCC derived from hURI-tetOFF$^{hep}$ ; SA$^{CreERT2}$ ; R26-stop-EYFP mice.

(K) Histogram representing GS classification used to determine HAGCKS score in HCA derived from hURI-tetOFF$^{hep}$ ; SA$^{CreERT2}$ ; R26-stop-EYFP mice.

(L) Representative images of GS IHC showing staining classified as low (1), intermediate (2) and high (3).

(M) Histogram representing CK19 classification used to determine HAGCKS score in HCC derived from hURI-tetOFF$^{hep}$; SA$^{CreERT2}$; R26-stop-EYFP mice.

(N) Histogram representing CK19 classification used to determine HAGCKS score in HCA derived from hURI-tetOFF$^{hep}$; SA$^{CreERT2}$; R26-stop-EYFP mice.

(O) Representative images of CK19 IHC showing negative (0) and positive (1) staining.

(P) Histogram representing Sox9 classification used to determine HAGCKS score in HCC derived from hURI-tetOFF$^{hep}$; SA$^{CreERT2}$; R26-stop-EYFP mice.

(Q) Histogram representing Sox9 classification used to determine HAGCKS score in HCA derived from hURI-tetOFF$^{hep}$; SA$^{CreERT2}$; R26-stop-EYFP mice.

(R) Representative images of Sox9 IHC showing staining classified as low (1), intermediate (2) and high (3).

(S) Histogram representing Ki67 classification used to determine HAGCKS score in HCC derived from hURI-tetOFF$^{hep}$; SA$^{CreERT2}$; R26-stop-EYFP mice.

(T) Histogram representing Ki67 classification used to determine HAGCKS score in HCA derived from hURI-tetOFF$^{hep}$; SA$^{CreERT2}$; R26-stop-EYFP mice.

(U) Representative images of Ki67 IHC showing staining classified as low (1), intermediate (2) and high (3).

(V) Representative images of H&E and IHC with different markers used in HAGCKS score in HCC or HCA derived from hURI-tetOFF$^{hep}$; SA$^{CreERT2}$; R26-stop-EYFP mice.

(W) Pie charts representing type of HCC derived from hepatocytes in Mdr2$^{KO}$ mice crossed with R26-stop-ZsGreen mice and infected with AAV8-Tbg$^{Cre}$ (n = 7) (left chart) or in R26-mTOM-

stop-mGFP mice infected with AAV8-Tgb$^{Cre}$ and treated with DEN and $CCl_4$ (n = 42) mice (right chart).

Scale bars represent 100 μm.

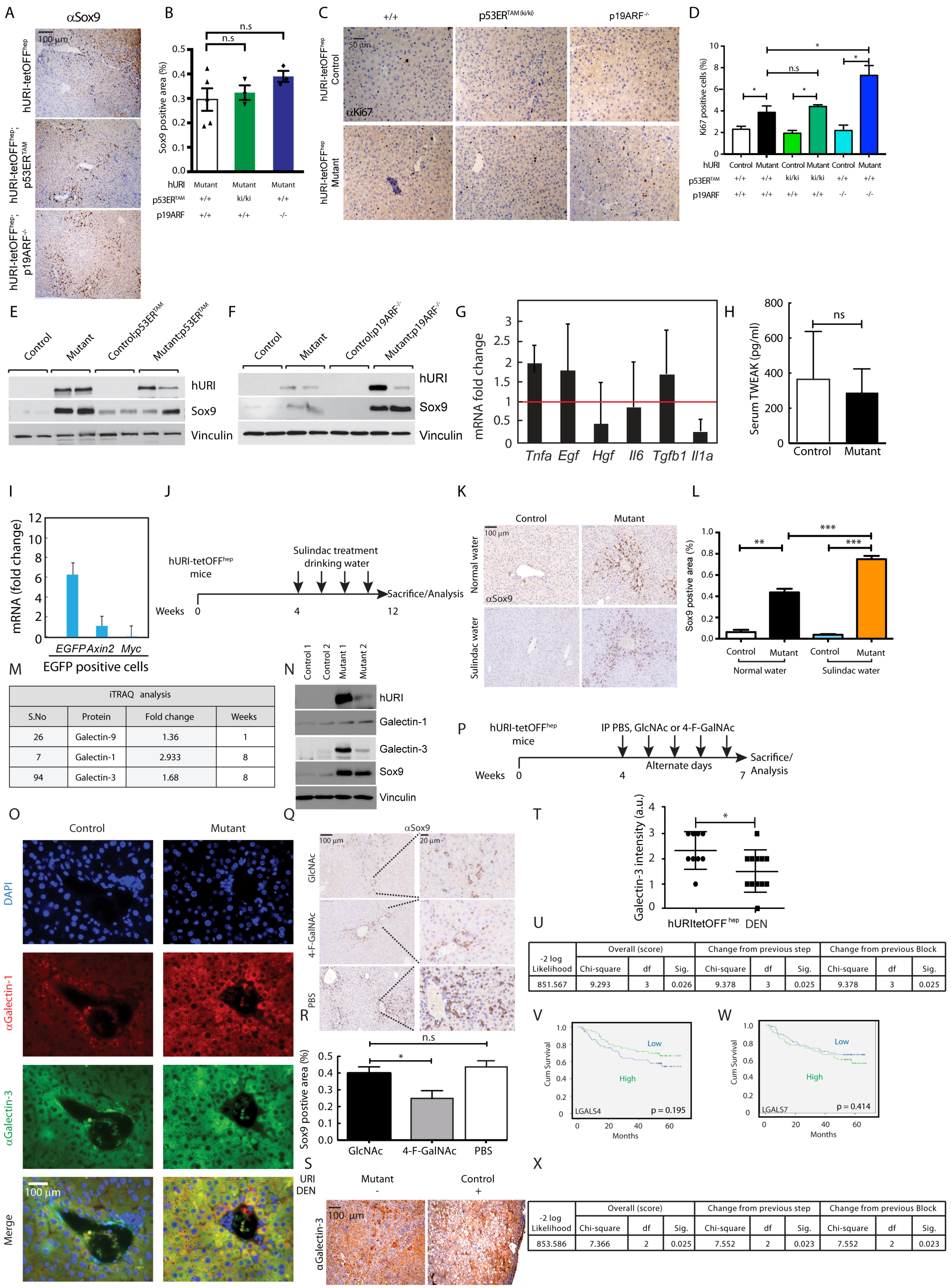


M

| iTRAQ analysis | | | |
|---|---|---|---|
| S.No | Protein | Fold change | Weeks |
| 26 | Galectin-9 | 1.36 | 1 |
| 7 | Galectin-1 | 2.933 | 8 |
| 94 | Galectin-3 | 1.68 | 8 |

U

| -2 log Likelihood | Overall (score) | | | Change from previous step | | | Change from previous Block | | |
|---|---|---|---|---|---|---|---|---|---|
| | Chi-square | df | Sig. | Chi-square | df | Sig. | Chi-square | df | Sig. |
| 851.567 | 9.293 | 3 | 0.026 | 9.378 | 3 | 0.025 | 9.378 | 3 | 0.025 |

X

| -2 log Likelihood | Overall (score) | | | Change from previous step | | | Change from previous Block | | |
|---|---|---|---|---|---|---|---|---|---|
| | Chi-square | df | Sig. | Chi-square | df | Sig. | Chi-square | df | Sig. |
| 853.586 | 7.366 | 2 | 0.025 | 7.552 | 2 | 0.023 | 7.552 | 2 | 0.023 |

**Figure S6. Hepatocytes Expressing Oncogenic URI Produce Galectin-3 to Instruct and Activate HPCs, Related to Figure 6**

(A) Representative images of Sox9 staining in liver sections derived from 12-week-old hURI-tetOFF$^{hep}$ , hURI-tetOFF$^{hep}$ ; p53ER$^{TAM}$ and hURI-tetOFF$^{hep}$ ; p19ARF$^{-/-}$ mice.

(B) Quantification of Sox9 IHC from (A). n ≥ 4.

(C) Representative images of Ki67 staining in liver sections derived from 12-week-old hURI-tetOFF$^{hep}$ , hURI-tetOFF$^{hep}$ ; p53ER$^{TAM}$ and hURI-tetOFF$^{hep}$ ; p19ARF$^{-/-}$ mice.

(D) Quantification of Ki67 IHC from (C). n ≥ 4.

(E) WB analysis of Sox9 expression in 12-week-old hURI-tetOFF$^{hep}$ and hURI-tetOFF$^{hep}$ ; p53ER$^{TAM}$ mice.

(F) WB analysis of Sox9 expression in 12-week-old hURI-tetOFF$^{hep}$ and hURI-tetOFF$^{hep}$ ; p19ARF$^{-/-}$ mice.

(G) qRT-PCR analysis of *Tnfa*, *Egf*, *Hgf*, *Il6*, *Tgfb1* and *Il1a* mRNA in livers derived from 8-week-old hURI-tetOFF$^{hep}$ mice. n = 4. Error bars represent ± SD.

(H) Serum levels of TWEAK cytokine in 8-week-old hURI-tetOFF$^{hep}$ mice. n = 3.

(I) qRT-PCR analysis of *Egfp*, *Axin2* and *Myc* mRNA from FACS-sorted SPCs from livers derived from 12-week-old hURI-tetOFF$^{hep}$ ; Sox9$^{IRES\text{-}EGFP}$ mice. n = 3. Error bars represent ± SD.

(J) Scheme representing sulindac-treated hURI-tetOFF$^{hep}$ mice. Mice were treated for 8 weeks since 4 weeks of age with 0.18 g/L sulindac in drinking water.

(K) Representative images of Sox9 staining in liver sections derived from 12-week-old hURI-tetOFF$^{hep}$ mice treated either with normal drinking water or with drinking water containing 0.18 g/L of sulindac.

(L) Percentage of Sox9-positive area from liver sections obtained from mice described in (J). n = 3 for controls and n = 4 for mutants under water, respectively. n = 4 and n = 6 for controls and mutants treated with sulindac, respectively.

(M) Table showing the fold change and rank (S. No) of galectin-9, galectin-1 and galectin-3 detected among differentially expressed proteins analyzed by iTRAQ performed in livers from 1- and 8-week old hURI-tetOFF$^{hep}$ mice as previously described (Tummala et al., 2014).

(N) Western blot analysis in 12-week-old hURI-tetOFF$^{hep}$. Immunoblots are performed using the indicated antibodies.

(O) Co-immunofluorescence of galectin-1 and galectin-3 in liver sections derived from 8-week-old hURI-tetOFF$^{hep}$ mice. n = 5.

(P) Scheme representing GlcNAc- and 4-F-GalNAc-treated hURI-tetOFF$^{hep}$ mice. At 4 weeks of age mice received I.P injection of either GlcNAc or 4-F-GalNAc every second day for 2 weeks.

(Q) Representative images of Sox9 staining in livers sections obtained from mice treated as described in (P). n = 7.

(R) Quantification of Sox9-positive area in livers described in (P). n = 7.

(S) Representative images of galectin-3 IHC in the tumors derived from hURI-tetOFF$^{hep}$ or DEN treated mice.

(T) Quantification of IHC from (S). n = 9 for hURI-tetOFF$^{hep}$-derived tumors. n = 12 for DEN-derived tumors.

(U) Multivariate Cox regression survival analysis for all the human galectin isoforms (*LGALS3*, *LGALS4* and *LGALS7*) in 221 patient human HCC gene expression analyses. (p=0.025). “df” and “Sig.” represents degrees of freedom and significance respectively.

(V) Kaplan Meier curve analysis of human HCC patient cumulative survival based on the expression of *LGALS4.* Degrees of freedom: 1, Chi-square: 1.680, $p = 0.195$.

(W) Kaplan Meier curve analysis of human HCC patient cumulative survival based on the expression of *LGALS7.* Degrees of freedom: 1, Chi-square: 0.667 $p = 0.414$.

(X) Multivariate Cox regression survival analysis for *LGALS3* and *MYC* in 221 patient human HCC gene expression analyses. ($p = 0.023$). "df" and "Sig." represents degrees of freedom and significance respectively.

Data are represented as mean ± SEM.

Scale bars represents 20 μm, 50 μm and 100 μm.

ns = non significant, $p \leq 0.05$ = *, $p \leq 0.01$ = ** and $p \leq 0.001$ = ***.

A

| S.No | Protein name | Symbol | Fold change 1 week | Fold change 8 weeks |
|---|---|---|---|---|
| 1 | Glycerol-3-phosphate dehydrogenase | Gpd1 | 1.469 | 0.855 |
| 2 | Pyruvate kinase isozymes R/L | Pk | 1.463 | 0.657 |
| 3 | Ketohexokinase | Khk | 1.448 | 0.692 |
| 4 | Glucose-6-phosphate 1-dehydrogenase | G6pd | 0.708 | 1.416 |
| 5 | Glutaminase liver isoform, mitochondrial | Gls2 | 0.664 | 0.330 |
| 6 | L-lactate dehydrogenase B chain | Ldh | 0.502 | 1.653 |
| 7 | Phosphoacetylglucosamine mutase | Pgm3 | 1.266 | 1.609 |
| 8 | L-Lactic dehydrogenase/lactate dehydrogenase | Ldh | 0.502 | 1.653 |
| 9 | Pyruvate kinase isozymes M1/M2 | Pkm | 0.843 | 1.677 |
| 10 | Pyruvate dehydrogenase [lipoamide]] kinase isozyme 2, mitochondrial | Pdhb | 0.989 | 0.797 |
| 11 | 2-oxoglutarate dehydrogenase E1 component, mitochondrial | Dhktd1 | 0.983 | 0.429 |
| 12 | Succinyl-CoA ligase [GDP-forming] subunit beta, mitochondria | Suclg | 1.053 | 0.673 |
| 13 | Succinate dehydrogenase cytochrome b560 subunit, mitochondrial | Sdhc | 1.020 | 0.703 |
| 14 | Pyruvate carboxylase, mitochondrial | Pcx | 1.102 | 0.697 |

B

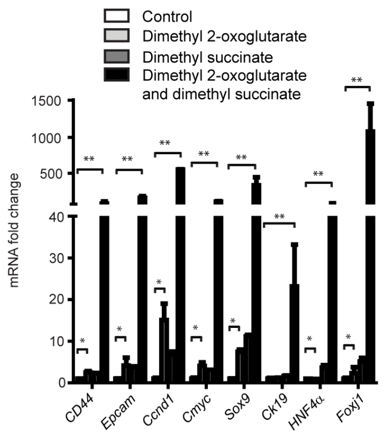


C

| -2 log Likelihood | Overall (score) Chi-square | df | Sig. | Change from previous step Chi-square | df | Sig. | Change from previous Block Chi-square | df | Sig. |
|---|---|---|---|---|---|---|---|---|---|
| 846.388 | 14.175 | 9 | 0.116 | 14.750 | 9 | 0.098 | 14.750 | 9 | 0.098 |

D

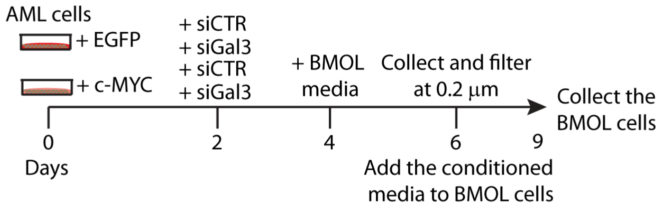


E

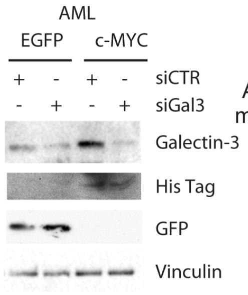


F

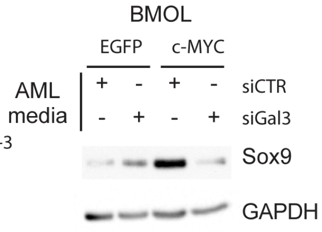


G

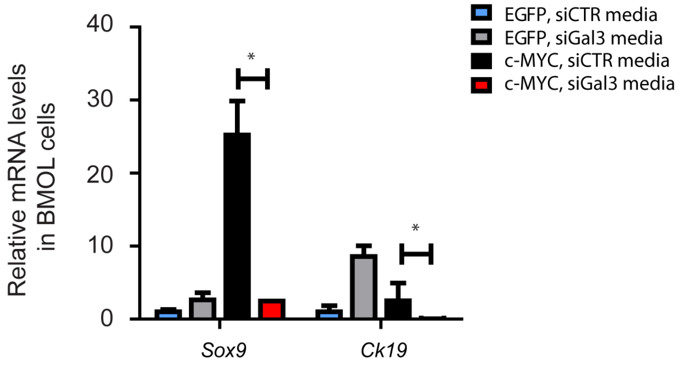

**Figure S7. Hepatocytes Expressing Oncogenic URI Produce α-ketoglutarate and Galectin-3 to Preserve HPC Undifferentiated State, Related to Figure 7**

(A) Table representing the differentially regulated proteins of intermediary metabolism obtained by iTRAQ analysis 1- and 8-week old mice previously described (Tummala et al., 2014).

(B) qRT-PCR analysis of BMOL cells treated with either 1 mM dimethyl 2-oxoglutarate (dimethyl-α-ketoglutarate), 4 mM dimethyl succinate or both for 24 h.

(C) Multivariate Cox regression analysis for the enzymes of TCA cycle (*IDH1*, *IDH2*, *IDH3A*, *IDH3B*, *IDH3G*, *OGDH*, *SUCLA2*, *SDHB* and *PC*) down regulated in hURItetOFF$^{hep}$ mouse model. (p=0.098). "df" and "Sig." represents degrees of freedom and significance respectively.

(D) Schematic representation of AML-12 and BMOL cell treatment. Media from EGFP or His-c-MYC overexpressing AML-12 cells transfected either with siCTR or siGal3 was used to culture BMOL cells for 3 days.

(E) WB analysis of AML-12 cells from (D). Membranes were blotted with the indicated antibodies.

(F) WB of BMOL cells after 72 hr incubation with media from AML-12 cells stably overexpressing EGFP or His-c-MYC and transfected with either siCTR or siGal3. Membrane was blotted with the indicated antibodies.

(G) qRT-PCR analysis of BMOL cells cultured with media from AML-12 cells as described in (D).

Data are represented as mean ± SEM. $p \leq 0.05$ = *; $p \leq 0.01$ = **.

## Supplemental Experimental Procedures

### Antibodies

Antibodies used for immunoblotting, immunofluorescence and immunohistochemistry were as follows: phospho-H2AX (Ser-139) (1:1000) (05-636), Cyp2E1 (1:200) (AB1252) and Sox9 (1:1000) (AB5535) were purchased from Millipore. CK19 (1:50) Troma-III was from CNIO Monoclonal Antibodies Core Unit. Glutamine synthetase (1:200) (G2781), His (1:100) (11922416001) and vinculin (1:1000) (V9131) were from Sigma Aldrich. Heat shock protein 70 (1:200) (sc-24) was from Santa Cruz Biotechnology. CD44 PE-Cy7 (1:100) (552849) was purchased from BD Pharmingen. CD326 (EpCAM)-APC (1:100) (17-5791) and CD133 (Prominin I) PerCP-eFluor 710 (1:100) (46-1331) were from eBioscience. GPR49 (Lgr5) (1:50) (ab75732), GFP (1:1000) (ab290), Laminin (1:200) (ab11575) and HNF4α (ab199431) were Abcam. DLK1 (1:100) (AF8277) and AFP (1:200) (AF5369) were from R&D systems. Galectin-1 (1:1000) (11858-1-AP) and Galectin-3 (1:1000) (60207-1-Ig) were purchased from Proteintech. Ki67 (1:1) (# MAD-000310QD) was purchased from Master Diagnostica. URI antibodies have been previously described (Buren et al., 2016; Tummala et al., 2014).

### Generation and Handling of Mice

hURI conditional knock-in (hURI-tetOFF$^{hep}$) was generated as recently reported (Tummala et al., 2014). Serum albumin (SA)-CreERT2 (Schuler et al., 2004), Sox9$^{IRES\text{-}CreERT2}$ (Furuyama et al., 2010) and Sox9$^{IRES\text{-}EGFP}$ (Nel-Themaat et al., 2009; Soeda et al., 2010) mice were kindly donated by Pierre Chambon, Yoshiya Kawaguchi and Haruhiko Akiyama, respectively. R26-stop-EYFP reporter line was previously reported and present in house (Srinivas et al., 2001). p19ARF$^{-/-}$

mouse was a gift from Manual Serrano. p53 inactivation in the hURI-tetOFF$^{hep}$ mouse was performed by crossing hURI-tetOFF$^{hep}$ mice with p53ER$^{TAM}$ mice, in which p53 activation requires ectopic 4-hydroxytamoxifen provision (Christophorou et al., 2005). Lgals3$^{\Delta/\Delta}$ mice were kindly obtained from Alison Mackinnon. R26-mTOM-stop-mGFP mice were previously reported (Muzumdar et al., 2007) and infected with AAV8-Tgb$^{Cre}$ and treated with DEN and $CCl_4$ as reported (Mu et al., 2015). Mdr2$^{KO}$ mice crossed with R26-stop-ZsGreen mice and infected with AAV8-Tgb$^{Cre}$ and CK19$^{CreERT2}$ ; R26-mTOM-stop-mGFP mice treated with DEN and $CCl_4$ were previously described (Mu et al., 2015). All mice have been housed in pathogen-free conditions. All experiments were approved by the CNIO-ISCIII Ethics Committee and performed in accordance with the guidelines for ethical conduct in the care and use of animals as stated in the international guiding principles for biomedical research involving animals, developed by the Council for International Organizations of Medical Sciences. Littermates were always used as controls. No gender differences were observed and age/developmental stage of mice was included appropriately in text and Figure legends. Food (Harlan Laboratories and Research Diets Inc.) and water were provided *ad libitum.*

**Mouse Treatment**

For diethylnitrosamine (DEN)-induced carcinogenesis, 14-day-old mice were injected intraperitoneally with 25 mg/kg of DEN (Sigma) according to the previously described protocol (Vesselinovitch and Mihailovich, 1983). After DEN injection, for tracking EYFP positive cells, Cre-mediated recombinase was activated by feeding mice with tamoxifen diet at the concentration of 400 mg/kg (supplemented in food) since weaning and for the next 2 weeks. Then after, mice were transferred to chow diet. Mice were sacrificed at the indicated time points

and the relative contribution of different cell types was assessed by histological methods and quantified.

N-Acetyl-D-glucosamine (GlcNAc) (Sigma-Aldrich (A8625)) and 4-Fluoro-N-acetylgalactosamine (4-F-GalNAc) (Sussex research laboratories (C8H14FNO6)) were dissolved in PBS. 3- or 4-week-old hURI-tetOFF$^{hep}$ mice were injected with 100 mg/kg of GlcNAc or 4-F-GalNAc intraperitoneally every second day for 3 weeks.

Nicotinamide riboside (NR) (97% purity, Waterstonetech Pharma) was mixed with ice-cold powdered food as previously reported (Tummala et al., 2014)

Sulindac (99% purity, Shanghai Moda Chemicals Co., Ltd) was dissolved in drinking water at the concentration of 0.18 g/L, and given to 4-week-old hURI-tetOFF$^{hep}$ mice for 8 weeks. Sulindac was changed every second day and freshly prepared.

Doxycyline was supplied in the diet ad libitum as previously described (Gomes et al., 2016; Tummala et al., 2014).

**Cell Culture, Transfection, Cell Number Counts**

Non tumorigenic alpha mouse liver (AML)-12 cells were grown in DMEM/F12 media supplemented with 10% fetal calf serum, 100 units/ml penicillin and 0.1 mg/ml streptomycin, 0.005 mg/ml insulin, 0.005 mg/ml transferrin, 5 ng/ml selenium, and 40 ng/ml dexamethasone purchased from Gibco (Wu etal., 1994). BMOL cells were cultured in Williams E media (Sigma-Aldrich) supplemented with 10% fetal calf serum, 100 units/ml of penicillin and 0.1 units/ml of streptomycin (Gibco), 30 ng/ml insulin-like growth factor (Novo enzymes), 20 ng/ml epidermal growth factor (BD Biosciences), 2 mM L-Glutamine (Sigma-Aldrich) and 0.25 U/ml Humulin R (Eli Lilly). Stable AML-12 cell lines overexpressing EGFP, HA-URI and His-c-Myc were

obtained by plasmid transfections using FUGENE 6 following the manufacturer instructions followed by 2 μg/ml puromycin selection for 3 days.

ON-TARGET plus SMART pool siRNA targeting murine Galectin-3 (siGal3) and control siRNA (siCTR) were purchased from Dharmacon. pcDNA3.1-HA-URI pcDNA-EGFP and pD40-His/V5-c-Myc and pcDNA-EGFP were previously described (Buren et al., 2016; Djouder et al., 2007). siRNA transfections were performed using Lipofectamine 3000 RNAiMAX following the manufacturer's instructions. For co-culture experiments, AML-12 cells were transfected with either siCTR or siGal3 or treated with either 10 μM BPTES or DMSO for 48 hr. Afterwards media was replaced by Williams E media and incubated for 24 hr. Pre-incubated media was then transferred to BMOL cells. Cells were lysed 72 hr later and processed for either protein or RNA isolation. BMOL cells were treated with 1 mM dimethyl 2-oxyglutarate (Sigma-Aldrich) or 4 mM dimethyl succinate (Sigma-Aldrich) or both for 24 hours. Cell number was measured in triplicates at the indicated times using Neubauer chamber.

**Flow Cytometry of HPCs**

**Isolation**

Liver isolated from mice was placed in ice cold DMEM/F12 and cut into small pieces with a scalpel. The obtained liver pulp was incubated in 10 ml of the enzyme solution containing 5 mg collagenase, 5 mg pronase and 1 ml DNAse in PBS in 37 °C water bath for 30 min to digest the tissue. Digested fraction was passed through 70 micron mesh filter and suspended in 20 ml of DMEM/F12 media supplemented with 10% FBS. Isolated cells were centrifuged 1 min at 50 g at 4 °C and the supernatant was collected. Step was repeated until no hepatocyte pellet was visible. Final supernatant fraction was centrifuged 8 min at 180 g in 4 °C to obtain fraction excluding

hepatocytes. Pellet was incubated for 5 min in 4 °C with 1 ml of red blood cell lysis solution to remove erythrocytes. Cells were centrifuged for 5 min at 200 g, pellet was used for fluorescence activated cell sorting (FACS).

**Fluorescence Activated Cell Sorting (FACS) Procedure**

Isolated cells were resuspended in 1ml of MACS buffer (0.1% BSA, 2 mM EDTA in $Ca^{2+}$/$Mg^{2+}$ free PBS) and incubated for 20 min on ice with Fc block (rat anti-mouse cd16/cd32) at the concentration of 1:200. Afterwards cells were washed with 300 µl of MACS buffer for 5 min and centrifuged for 2 min at 200 g at 4°C. Pellet was resuspended in 200 µl of MACS buffer and incubated for 20 min at 4 °C with primary antibodies (CD44 1:50, EpCAM 1:50, CD133-Percp 1:50, LGR5 1:10, DLK1 1:50). Then after cells were washed 2 times with MACS buffer, resuspended in 300 µl of MACS buffer and incubated for 15 min at 4 °C with secondary antibodies (Alexa-Fluor-680 1:1000, Alexa-Fluor-555 1:1000). Then, cells were washed 2 times with MACS buffer and resuspended in 300 µl of MACS buffer. DAPI (1:100) was added and, cells were passed through a filter and processed for FACS analysis using BD LSRFortessa machine.

Compensation beads were prepared for CD133-PE-Cy7, EpCAM-APC and CD44-Percp antibodies raised in mouse and rat using OneComp ebeads (ebiosciences). Compensation for DLK1 and LGR5 was prepared by using the cells from the control (not containing fluorophore) and primary antibodies (DLK1 1:50, LGR5 1:10)

FMO (Fluorescence minus one) was prepared separately for each primary antibody used. 100 µl of cells isolated from each mutant was mixed together and 100 µl of the mixture was used for each FMO. Cells were resuspended in 100 µl of MACS buffer and incubated for 20 min in 4 °C

with primary antibodies according to the FMO prepared (CD44 1:50, EpCAM 1:50, CD133-Percp 1:50, LGR5 1:10, DLK1 1:50). Cells were washed with 300 µl of MACS buffer, resuspended in 300 µl of MACS buffer and if necessary for the FMO incubated for 15 min in 4°C with secondary antibody (Alexa-Fluor-680 1:1000, Alexa-Fluor-555 1:1000). Cells were washed 2 times with MACS buffer and resuspended in 300 µl of MACS buffer. DAPI (1:100) was added, cells were passed through a filter and processed for FACS analysis.

**FACS Analysis**

FACS data were analyzed using FlowJo (www.flowjo.com) software using the single color FMO's and sample data acquired without any fluorophore as control.

**Genotyping and qRT-PCR**

For genotyping toe DNA was extracted by overnight incubation of toes with 500 µl of the cell lysis buffer (1% SDS, 0.1 M NaCl, 0.1 M EDTA, 0.05 M Tris (pH 8) and 400 µg/ml of proteinase K). Extracted DNA after saturated salt precipitation was precipitated using ice-cold isopropanol which was further washed with 80% ethanol. The DNA pellet was further dried and resuspended in 400 µl of distilled water. 1 µl of DNA was used for genotyping. For the *ColhURI* locus the following primers were used: P1-F: GCACAGCATTGCGGACATGC; P2-R: CCCTCCATGTGTGACCAAGG; P3-R: GCAGAAGCGCGGCCGTCTGG. For the *LaptTA* transactivator locus the following primers were used: P1-F: TCTGAGCATGGCCTCTAA; P2-R: GCTGGAGTAAATTTCACAGTG; P3-R: TCTCACTCGGAAGGACAT. For $SA^{CreERT2}$ the following primers were used: P1: ATCATTTCTTTGTTTTCAGG, P2: GGAACCCAAACTGATGACCA and P3: TTAAACAAGCAAAACCAAAT. For $Sox9^{IRES-}$

CreERT2 the following primers were used: P1: TCCAATTTACTGACCGTACAC CAA and P2: CCTGATCCTGGCAATTTCGGCTA. For R26: P1: TGACCCTGAAGTTCATCTGCA and P2: TCACGAACTCCAGCAGGACCA. For EYFP: P1: TGACCCTGAAGTTCATCTGCA and P2: TCACGAACTCCAGCAGGACCA. For *Lgals3Δ/Δ*: P1: GAGGAGGGTCAAAGGGAAAG, P2: GACTGGAATTGCCCATGAAC and P3: TCGCCTTCTTGACGAGTTCT.

For qRT-PCR, total RNA was extracted from 20-50 mg of liver or cells as described in (Tummala et al., 2014) and qRT-PCR was performed with the following primers. Human-*Uri*-F: GAAAGACTCAGCACCTTGCC. Human-*Uri*-R: TTCCGGTGCTCAACTAAACC. Mouse-*Myc*-F: TGACCTAACTCGAGGAGGAGCTGGAATC, Mouse-*Myc*-R: AAGTTTGAGGCAGTTAAAATTATGGCTGAAGC. Mouse-*Axin*-F: GAATCTGCATGGGCAACC . Mouse-*Axin*-R: GAATCCTGGTACATCTGGGAAC. *Gfp*-F: TGACCCTGAAGTTCATCTGCA. *Gfp*-R: TCACGAACTCCAGCAGGACCA. Mouse-*TNFA*–F: AGTTCTATGGCCCAGACCCT. Mouse-*TNFA*-R: CGGACTCCGCAAAGTCTAAG. Mouse-*Egf*–F: ACATAGATGGAATGGGCACAGG. Mouse-*Egf* –R: CCTCTAGAAATGTGGTATGGCTG. Mouse-*Hgf*–F: TTCCCAGCTGGTCTATGGTC Mouse-*Hgf*–R: GGTGCTGACTGCATTTCTCA. Mouse-*Il6*–F: GCTACCAAACTGGATATAATCAGGA Mouse-*Il6*–R: CCAGGTAGCTATGGTACTCCAGAA. Mouse-*Tgfb*–F: CGCCCGGGTTGTGTTGGTTGTAGA Mouse-*Tgfb*–R: CTGACCCCCACTGATCCTGAG. Mouse-*Il1a*–F: AAGATGTCCAACTTCACCTTCAAGGAGAGCCG. Mouse-*Il1a*–R: AGGTCGGTCTCACTAACTGTGATGAGTTTTGG Mouse-*Actb*-F: CACAGCTGAGAGGGAAATCG. Mouse-*Actb*-R: AGTTTCATGGATGCCACAGG. Mouse-

*Cd44*-F: TGCATTTGGTGAACAAGGAA. Mouse-*Cd44*-R: GGAATGACGTCTCCAATCGT. Mouse-*Epcam*-F: GCGGCTCAGAGAGACTGTG. Mouse-Epcam-R: CCAAGCATTTAGACGCCAGTTT. Mouse-*Ccnd1*-F: TCAAGTGTGACCCGGACTG. Mouse-*Ccnd1*-R: ATGTCCCACATCTCGCACGTC. Mouse-*Sox9*-F: TGCCCATGCCCGTGCGCGTCAA. Mouse-*Sox9*-R: CGCTCCGCCTCCTCCACGAAGGGTCT. Mouse-*Ck19*-F: GGACCCTCCCGAGATTACAACCA. Mouse-*Ck19*-R: GCCAGCTCCTCCTTCAGGCTCT. Mouse-*Hnf4a*-F: CGGGCTGGCATGAAGAAGGAAG. Mouse-*Hnf4a*-R: TGGGAGAGGTGATCTGCTGGGA. Mouse-*Foxj1*-F: CCGCCATGCAGACCCCACCTGGCA. Mouse-*Foxj1*-R: AGGCCCCACTGAGCAGGCGCTCT

**Supplemental References**

Buren, S., Gomes, A.L., Teijeiro, A., Fawal, M.A., Yilmaz, M., Tummala, K.S., Perez, M., Rodriguez-Justo, M., Campos-Olivas, R., Megias, D*., et al.* (2016). Regulation of OGT by URI in Response to Glucose Confers c-MYC-Dependent Survival Mechanisms. Cancer Cell *30*, 290-307.

Christophorou, M.A., Martin-Zanca, D., Soucek, L., Lawlor, E.R., Brown-Swigart, L., Verschuren, E.W., and Evan, G.I. (2005). Temporal dissection of p53 function in vitro and in vivo. Nat Genet *37*, 718-726.

Djouder, N., Metzler, S.C., Schmidt, A., Wirbelauer, C., Gstaiger, M., Aebersold, R., Hess, D., and Krek, W. (2007). S6K1-mediated disassembly of mitochondrial URI/PP1gamma complexes

activates a negative feedback program that counters S6K1 survival signaling. Mol Cell *28*, 28-40.

Muzumdar, M.D., Tasic, B., Miyamichi, K., Li, L., and Luo, L. (2007). A global double-fluorescent Cre reporter mouse. Genesis *45*, 593-605.

Nel-Themaat, L., Vadakkan, T.J., Wang, Y., Dickinson, M.E., Akiyama, H., and Behringer, R.R. (2009). Morphometric analysis of testis cord formation in Sox9-EGFP mice. Dev Dyn *238*, 1100-1110.

Schuler, M., Dierich, A., Chambon, P., and Metzger, D. (2004). Efficient temporally controlled targeted somatic mutagenesis in hepatocytes of the mouse. Genesis *39*, 167-172.

Soeda, T., Deng, J.M., de Crombrugghe, B., Behringer, R.R., Nakamura, T., and Akiyama, H. (2010). Sox9-expressing precursors are the cellular origin of the cruciate ligament of the knee joint and the limb tendons. Genesis *48*, 635-644.

Srinivas, S., Watanabe, T., Lin, C.S., William, C.M., Tanabe, Y., Jessell, T.M., and Costantini, F. (2001). Cre reporter strains produced by targeted insertion of EYFP and ECFP into the ROSA26 locus. BMC Dev Biol *1*, 4.

Tummala, K.S., Gomes, A.L., Yilmaz, M., Grana, O., Bakiri, L., Ruppen, I., Ximenez-Embun, P., Sheshappanavar, V., Rodriguez-Justo, M., Pisano, D.G., *et al.* (2014). Inhibition of de novo NAD(+) synthesis by oncogenic URI causes liver tumorigenesis through DNA damage. Cancer Cell *26*, 826-839.

Vesselinovitch, S.D., and Mihailovich, N. (1983). Kinetics of diethylnitrosamine hepatocarcinogenesis in the infant mouse. Cancer research *43*, 4253-4259.
