# Supplementary material for: Hepatocellular Carcinomas Originate Predominantly from Hepatocytes and Benign Lesions from Hepatic Progenitor Cells
Source: Cell Rep. 2017 Apr 19;19(3):584–600. doi: 10.1016/j.celrep.2017.03.059 (PMC5409928; doi:10.1016/j.celrep.2017.03.059)
Supplement: Document S2. Article plus Supplemental Information [file mmc2.pdf]

Cell Reports

Article

Hepatocellular Carcinomas Originate Predominantly from Hepatocytes and Benign Lesions from Hepatic Progenitor Cells

Graphical Abstract

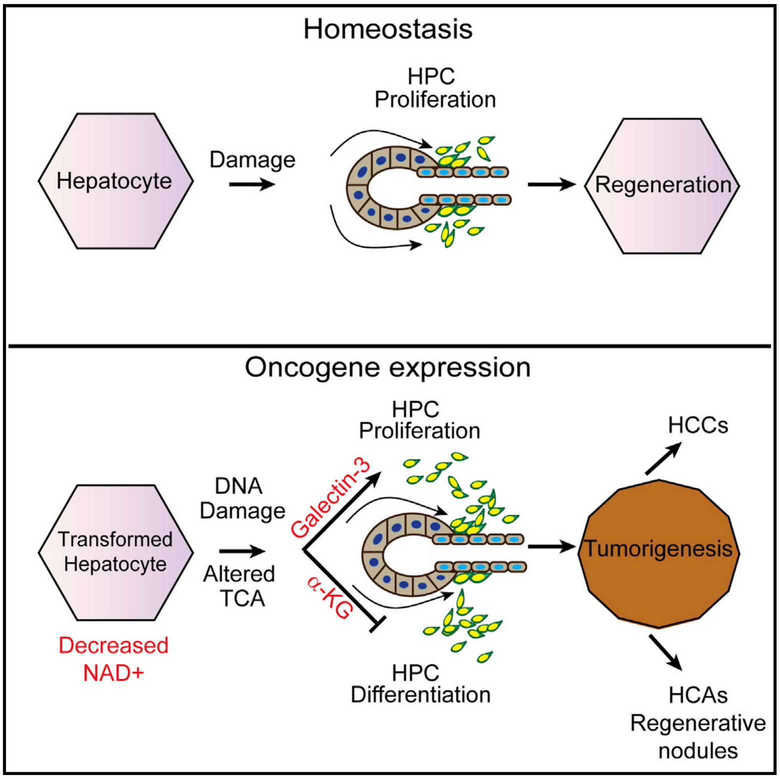

Authors

Krishna S. Tummala, Marta Brandt, Ana Teijeiro, Osvaldo Graña, Robert F. Schwabe, Cristian Perna, Nabil Djouder

Correspondence

ndjouder@cnio.es

In Brief

Tummala et al. demonstrate that hepatocytes and hepatic progenitor cells participate in liver tumor heterogeneity. Malignant hepatocytes crosstalk and activate the neighboring progenitor cells through releases of galectin-3 and α-ketoglutarate. Blocking galectin-3 reduces hepatocarcinogenesis and may have important clinical implications.

Highlights

- HPCs expand in the early stages of hepatocarcinogenesis
- HCC predominantly originates from hepatocytes
- Oncogene-induced hepatocyte transformation activates HPCs via galectin-3 and αKG
- Activated HPCs contribute to liver tumor heterogeneity

Tummala et al., 2017, Cell Reports *19*, 584–600
April 18, 2017 © 2017 The Author(s).
http://dx.doi.org/10.1016/j.celrep.2017.03.059

OPEN ACCESS
CellPress

Cell Reports
Article

Hepatocellular Carcinomas Originate Predominantly from Hepatocytes and Benign Lesions from Hepatic Progenitor Cells

Krishna S. Tummala,[1,5] Marta Brandt,[1,5] Ana Teijeiro,[1] Osvaldo Graña,[2] Robert F. Schwabe,[3] Cristian Perna,[4] and Nabil Djouder[1,6,*]
[1]Cancer Cell Biology Programme, Growth Factors, Nutrients and Cancer Group
[2]Structural Biology and Biocomputing Programme, Bioinformatics Unit
Centro Nacional de Investigaciones Oncológicas (CNIO), Madrid 28029, Spain
[3]Department of Medicine, Columbia University, New York, NY 10032, USA
[4]Department of Pathology, Hospital Universitario Ramón y Cajal, IRYCIS, Madrid 28034, Spain
[5]These authors contributed equally
[6]Lead Contact
*Correspondence: ndjouder@cnio.es
http://dx.doi.org/10.1016/j.celrep.2017.03.059

SUMMARY

Hepatocellular carcinoma (HCC) is an aggressive primary liver cancer. However, its origin remains a debated question. Using human data and various hepatocarcinogenesis mouse models, we show that, in early stages, transformed hepatocytes, independent of their proliferation status, activate hepatic progenitor cell (HPC) expansion. Genetic lineage tracing of HPCs and hepatocytes reveals that, in all models, HCC originates from hepatocytes. However, whereas in various models tumors do not emanate from HPCs, tracking of progenitors in a model mimicking human hepatocarcinogenesis indicates that HPCs can generate benign lesions (regenerative nodules and adenomas) and aggressive HCCs. Mechanistically, galectin-3 and α-ketoglutarate paracrine signals emanating from oncogene-expressing hepatocytes instruct HPCs toward HCCs. α-Ketoglutarate preserves an HPC undifferentiated state, and galectin-3 maintains HPC stemness, expansion, and aggressiveness. Pharmacological or genetic blockage of galectin-3 reduces HCC, and its expression in human HCC correlates with poor survival. Our findings may have clinical implications for liver regeneration and HCC therapy.

INTRODUCTION

Hepatocellular carcinoma (HCC) and intrahepatic cholangiocarcinoma (ICC) are the most lethal primary forms of liver cancers. Rarer benign hepatic tumors include focal nodular hyperplasias and hepatocellular adenomas (HCAs), which may progress to HCC (Pilati et al., 2014). Regenerative nodules (RN), common in cirrhotic liver, are also likely HCC precursors (Lin et al., 2010).

Hepatocytes represent the most functionally active parenchymal liver cells (>80%). Others are the bile duct epithelial cells or ductular cells (DCs). DCs originate from the canal of Hering and are considered to be the bipotential hepatic progenitor cells (HPCs) also known as transit-amplifying cells or oval cells in rodent models or liver progenitor cells in humans. The canal of Hering is therefore considered the putative hepatic stem cell niche in the adult liver. HPCs have a transcriptomic profile similar to DCs (He et al., 2013) as well as the ability to replenish damaged hepatocytes and cholangiocytes (Carpentier et al., 2011; Espanol-Suner et al., 2012; Lu et al., 2015; Wang et al., 2003).

The liver has remarkable plasticity following injury and is the only adult organ with complete regenerative potential. Depending on the injury (acute or chronic), liver regeneration may involve activation of either fully differentiated hepatocytes or HPCs. Fate-tracing studies indicate that hepatocytes are mainly responsible for liver maintenance under normal conditions and responses to acute liver injury (Malato et al., 2011). Acute liver damage, partial hepatectomy or chemical damage by carbon tetrachloride (CCl_4), may induce replication of mature hepatocytes, which replenishes the liver and maintains its homeostasis.

Impairment of hepatocyte replicative potential can induce the emergence of HPCs, which are required to continuously supply new hepatocytes and maintain the organ's functional integrity during chronic liver damage. HPC expansion is mainly due to various pathologies, environmental or genetic etiologies of human HCC, primarily affecting hepatocytes and leading to chronic liver damage in the early stages of tumorigenesis (e.g., fibrosis, cirrhosis, steatosis, inflammation, viral infection, aflatoxin, α-1 antitrypsin deficiency, Wilson's disease, and hemochromatosis). HCC progression is thus often accompanied by HPC activation, but mechanisms initiated by injured hepatocytes remain still elusive.

Cancer stem cell markers (EpCam, CD133, and CD44), specific cytokeratins (CKs; 7 and 19), and the sex-determining

 © 2017 The Author(s).
This is an open access article under the CC BY license (http://creativecommons.org/licenses/by/4.0/).

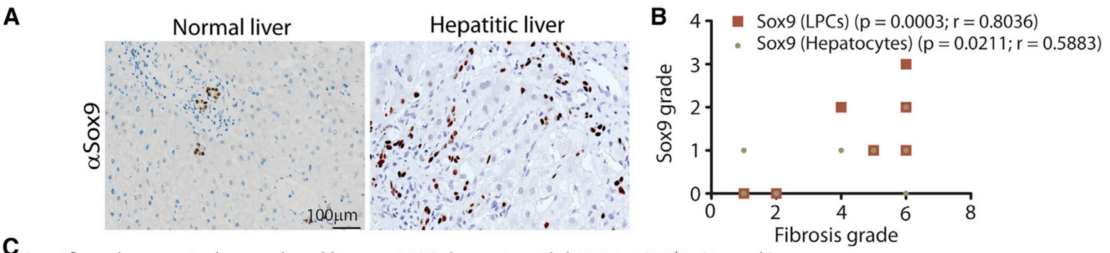

C Significantly negatively correlated human HCC datasets with hURI-tetOFF^{hep} (1 week)

S.No	MsigDB human HCC data set	NES	FDR q-value	Summary
1	BOYAULT_LIVER_CANCER_SUBCLASS_G5_DN	-2.102	0.0044	Active WNT pathway
2	YAMASHITA_LIVER_CANCER_STEM_CELL_DN	-1.767	0.0971	HCC with stem cell signature

Significantly negatively correlated human HCC datasets with hURI-tetOFF^{hep} (8 weeks)

S.No	MsigDB human HCC data set	NES	FDR q-value	Summary
1	LEE_LIVER_CANCER_SURVIVAL_UP	-2.115	0.0026	Poor patient survival
2	IIZUKA_LIVER_CANCER_PROGRESSION_G2_G3_UP	-2.024	0.0030	Poorly differentiated HCC
3	BOYAULT_LIVER_CANCER_SUBCLASS_G3_DN	-1.772	0.0401	Activated TP53
4	YAMASHITA_LIVER_CANCER_STEM_CELL_DN	-1.721	0.0413	HCC with stem cell signature
5	WOO_LIVER_CANCER_RECURRENCE_DN	-1.678	0.0473	HCC recurrence
6	BOYAULT_LIVER_CANCER_SUBCLASS_G1_DN	-1.559	0.0828	Fetal liver gene signature
7	IIZUKA_LIVER_CANCER_PROGRESSION_L1_G1_DN	-1.518	0.0917	Poorly differentiated HCC

Significantly positively correlated human HCC datasets with hURI-tetOFF^{hep} (8 weeks)

S.No	MsigDB human HCC data set	NES	FDR q-value	Summary
1	WOO_LIVER_CANCER_RECURRENCE_UP	2.005	<0.0001	HCC recurrence
2	HOSHIDA_LIVER_CANCER_SUBCLASS_S1	1.874	0.0005	WNT pathway activation
3	LIAO_METASTASIS	1.855	0.0006	Metastasis signature
4	YAMASHITA_LIVER_CANCER_STEM_CELL_UP	1.7389	0.0075	HCC with stem cell signature
5	BOYAULT_LIVER_CANCER_SUBCLASS_G5_DN	1.7244	0.0086	Active WNT pathway
6	BOYAULT_LIVER_CANCER_SUBCLASS_G1_UP	1.7154	0.0085	Fetal liver genes signature
7	COULOUARN_TEMPORAL_TGFB1_SIGNATURE_UP	1.6709	0.0141	Late TGFb signature
8	ROESSLER_LIVER_CANCER_METASTASIS_UP	1.6529	0.0169	Metastasis signature
9	SAKAI_TUMOR_INFILTRATING_MONOCYTES_UP	1.6317	0.0202	Monocyte infiltration
10	KUROKAWA_LIVER_CANCER_CHEMOTHERAPHY_DN	1.6168	0.0224	Downregulated in 5-FU and anti-INF1 chemotherapy

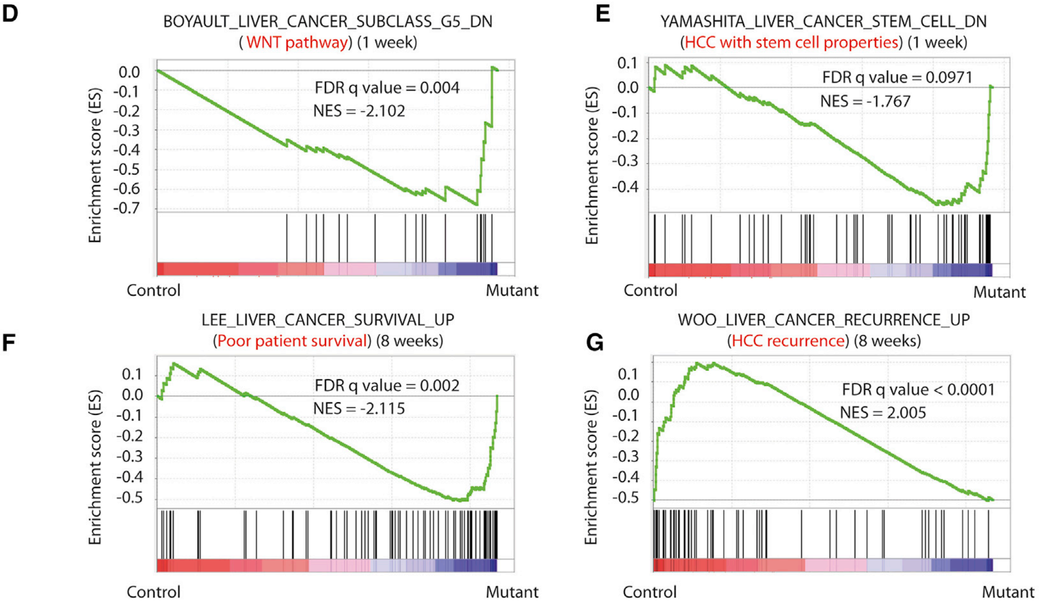

(legend on next page)

region Y-box (SRY-box) containing the transcription factor Sox9 (Furuyama et al., 2011; Rountree et al., 2012; Yamashita et al., 2009) aid recognition of DCs and HPCs. DCs and HPCs remain negative for hepatocyte nuclear factor 4 alpha (HNF4α), a marker of mature hepatocytes and hybrid periportal hepatocytes (HybHPs) (Font-Burgada et al., 2015). A recent report indicated that HybHPs expressing both Sox9 and HNF4α may participate in liver regeneration, but not HCC (Font-Burgada et al., 2015). Long-debated uncertainties are thus whether DCs and HPCs are hepatic cancer stem cells (CSCs), and whether they participate in hepatocarcinogenesis. According to recent proposals, cells with similar transcriptomic profiles to oval cells may be HCC progenitors (He et al., 2013; Holczbauer et al., 2013). Furthermore, liver carcinomas derived from genetically modified bipotential liver progenitors resemble features of the human disease (Zender et al., 2005). Recently, hepatocytes were suggested to be the sole cell of origin for HCC (Mu et al., 2015), and HPCs or DCs do not produce tumors in genetic and chemical HCC mouse models (Jörs et al., 2015; Mu et al., 2015). Certainly, due to scarcities of animal models recapitulating human liver tumorigenesis, the cells of origin of liver cancer as well as the origin of its heterogeneity (benign lesions, RN-induced cirrhosis, HCAs, and HCCs) are not clear yet and may be context dependent.

A mouse model (hURI-tetOFF^{hep}) we recently generated expresses hepatocyte-specific human unconventional prefoldin RPB5 interactor (hURI). In this model, NAD^{+}-deficit-induced DNA damage causes multistep liver tumor development somehow mimicking human hepatocarcinogenesis, with development of focal nodular hyperplasia, RN, non-alcoholic steatohepatitis (NASH), HCAs, and HCCs (Gomes et al., 2016; Tummala et al., 2014). Using this model and several other models, combined with genetic lineage tracing and in vitro co-culture cell experiments, we evaluate here the contribution of hepatocytes and HPCs in hepatocarcinogenesis. We also report mechanisms emerging from hepatocytes and regulating HPC expansion in the early stages of tumorigenesis, through metabolic paracrine signals, implicating galectin-3 and α-ketoglutarate.

RESULTS

hURI-tetOFF^{hep} Mice Display HPC Signatures in the Early Stages of Hepatocarcinogenesis Correlating with Aggressive Human HCC

Human HCC develops via pre-neoplastic lesions that frequently arise within a background of chronic inflammatory liver damage or hepatitis progressing to fibrosis, cirrhosis, and eventually HCC. Premalignant lesions contain Sox9-positive progenitors (considered to be HPCs) (He et al., 2013). Sox9 expression correlates with degrees of liver-damage-associated fibrosis, according to immunohistochemistry (IHC) of human hepatitic samples (Figures 1A and 1B). Thus, Sox9-positive cells (SPCs) expand in early human hepatocarcinogenesis.

Using public expression data applied to classify human HCC subtypes (Figures 1 and S1), we identified gene sets that are up- or downregulated in hURI-tetOFF^{hep} livers in early tumorigenesis stages (Tummala et al., 2014) and correlate with therapy resistance and poor prognosis according to gene set enrichment analysis (GSEA). Normalized enrichment scores and false discovery rates indicated that 1-week-old hURI-tetOFF^{hep} mice have a signature very similar to G5 subtype HCC, which is strongly related to β-catenin mutations and thus WNT activation (Boyault et al., 2007), which is essential for HPC expansion (Figures 1C and 1D). Further evaluation showed that an HPC signature associated with WNT/β-catenin signaling is significantly enriched in 1- and 8-week-old hURI-tetOFF^{hep} mice (Figures 1C and 1E) (Yamashita et al., 2009).

GSEA also detected: significant overlaps between transcriptomic signatures of 8-week-old hURI-tetOFF^{hep} mouse and the S1 HCC subclass, confirming that in early stages the model exhibits an aberrant WNT signaling pathway signature (Hoshida et al., 2009) (Figures 1C and S1A); fetal liver and p53 pathway activation signatures, respectively present in G1 and G3 subtypes (Boyault et al., 2007) (Figures 1C and S1B); and a signature associated with transforming growth factor β (TGF-β) signaling pathways that reportedly activate HPCs (Coulouarn et al., 2008) (Figure 1C).

Significant enrichment of genes associated with poor patient survival, including stem cell genes, was detected in data from 8-week-old hURI-tetOFF^{hep} mice (Figures 1C and 1F) (Lee et al., 2004) and a metastasis gene signature predicting tumor relapse in early-stage HCC patients (Figures 1C and S1C) (Liao et al., 2008; Roessler et al., 2010). Further analysis revealed that these stem-like liver cancer subtypes represent a class of poorly differentiated HCC (Figures 1C and S1D) (Iizuka et al., 2005). Moreover, stem-like liver cancers from relapsing patients showed a signature enriched in 8-week-old hURI-tetOFF^{hep} mice (Figures 1C and 1G) (Woo et al., 2008). Thus, in early stages, hURI-tetOFF^{hep} livers display a stem cell-like signature found in aggressive human HCC.

HPCs Expand in the Early Stages of Hepatocarcinogenesis

Upregulated proteins identified in published proteomic (isobaric tags for relative and absolute quantification [iTRAQ]) profiles of

Figure 1. hURI-tetOFF^{hep} Mice Display HPC Signatures in Early Hepatocarcinogenesis Stages Correlating with Aggressive Human HCC

(A) Representative images of Sox9 immunohistochemistry (IHC) in human hepatitis samples. Sox9 expression was evaluated in the liver progenitor cells (LPCs) and hepatocytes (n = 15).

(B) Graph representing the correlation between fibrosis grade and Sox9 expression in human hepatitis samples (n = 15).

(C) Table summarizing the most significantly enriched human HCC gene sets in 1- or 8-week-old hURI-tetOFF^{hep} mice.

(D) GSEA of human HCC WNT pathway and 1-week-old hURI-tetOFF^{hep} mice protein signature.

(E) GSEA of human HCC stem cell properties and 1-week-old hURI-tetOFF^{hep} mice protein signature.

(F) GSEA of human HCC poor patient survival and 8-week-old hURI-tetOFF^{hep} mice protein signature.

(G) GSEA of human HCC recurrence and 8-week-old hURI-tetOFF^{hep} mice protein signature. All protein signature datasets obtained for hURI-tetOFF^{hep} mice were previously described and achieved by iTRAQ (Tummala et al., 2014).

Normalized enrichment score (NES) and false discovery rate (FDR) q-values are indicated in each graph. Scale bar represents 100 μm.

8-week-old hURI-tetOFF^{hep} mouse livers (Tummala et al., 2014) included several DC markers and proteins of the extracellular matrix (ECM) (e.g., laminin, vimentin, and transgelin) essential for oval cell expansion (Figure S2A). Notably, hURI was specifically expressed in hepatocytes, but not in DCs (Figure 2A). Thus, hURI-tetOFF^{hep} mice were crossed with Sox9$^{IRES\text{-}EGFP}$ mice expressing EGFP from the *Sox9* promoter primarily in DCs (Supplemental Experimental Procedures). EGFP/SPCs were isolated by fluorescence-activated cell sorting (FACS). qRT-PCR of isolated EGFP-positive cells and whole mutant livers (hereafter, "mutants" refers to mice ectopically expressing hURI) confirmed that hURI is specifically expressed in hepatocytes (Figure S2B). Interestingly, IHC and western blot (WB) of Sox9 and CK19 markers confirmed the presence of a ductular reaction in mutant livers (Figures 2B, 2C, and S2C). We detected DC expansion in mutant livers when preneoplastic lesions were apparent, in 8- to 24-week-old mutant livers, but not in non-pathological 3-week-old livers expressing hURI (Figure 2B). Importantly, increased laminin was confirmed by IHC (Figures S2D and S2E). SPCs also expanded in 7-week-old C57BL/6 mice treated with the diethylnitrosamine (DEN) carcinogen known to induce HCC (Figures S2F and S2G) (Tummala et al., 2014). Thus, SPCs expand during liver tumorigenesis.

Co-immunofluorescence (co-IF) using Sox9 and CK19 antibodies in hURI-tetOFF^{hep} liver sections revealed that out of the total number of cells expressing either Sox9 or CK19, ~15% were positive for only Sox9, 60% were CK19 positive, and 30% were positive for both (Figures S2H and S2I). Thus, SPCs comprise a small subset of the highly heterogeneous DC population.

We subsequently checked other DC/HPC markers by FACS-sorting EGFP$^+$ SPCs from liver cells of 12-week-old mice generated from an hURI-tetOFF^{hep} and Sox9$^{IRES\text{-}EGFP}$ cross (Supplemental Experimental Procedures). The expanded EGFP$^+$ SPCs in mutant mice represented 5.76% ± 2.7% of the liver fraction excluding hepatocytes but only 0.9% ± 1% in their littermates (Figure 2D). EGFP cells were positive for the CSC markers EpCAM, CD133, and CD44 (95.5% ± 1.79%; 94.0% ± 1.51%, and 21.2% ± 3.81%, respectively). However, a small proportion of EGFP$^+$ SPCs was positive for LGR5 (8.23% ± 1.79%) (Huch et al., 2013b) and DLK1 (3.23% ± 1.20%) (Xu et al., 2012) markers (Figure 2D). SPCs thus represent a heterogeneous DC population with stem cell characteristics and may be considered as hepatic CSCs or HPCs.

HPCs Contribute to Liver Tumorigenesis

Next, we tracked SPCs during liver tumorigenesis by crossing Sox9$^{IRES\text{-}CreERT2}$ and reporter R26-stop-EYFP. In this context, SPCs express an inducible Cre recombinase, which specifically deletes the *loxP*-flanked stop cassette in the Rosa26R reporter locus. This occurs in genomic DNA, so all descendants of labeled cells inherit enhanced yellow fluorescent protein (EYFP) expression. For confirmation, 5-week-old mice were fed a tamoxifen diet for 2 weeks and then transferred to chow (Figure 3A). Tamoxifen-activated Cre recombinase permanently EYFP-labeled the emerging SPCs (Figure 3B). Importantly, the tamoxifen dose (400 mg/kg supplied in food) and exposure time (2 weeks) did not induce the hepatocyte toxicity known to increase Sox9 levels in hepatocytes as previously reported (Carpentier et al., 2011) (Figure 3B). Co-IF of Sox9 and EYFP revealed a 44.2% ± 7.7% recombination efficiency (Figure 3C). Therefore, only about 40% of the SPCs are genetically tracked in this model. Importantly, after 2 weeks of tamoxifen treatment, we did not detect any cells positive for Sox9 and HNF4α (a marker of mature hepatocytes) in Sox9$^{IRES\text{-}creERT2}$; R26-stop-EYFP mice (Figures 3D and 3E). Confocal microscopy and quantification confirmed that EYFP and HNF4α did not co-localize (Figures 3F and 3G). Additionally, Sox9 expression was solely restricted to SPCs in the hURI-tetOFF^{hep} model after 2 weeks of tamoxifen treatment (Figures 3H and 3I). Importantly, we did not detect the presence of hybrid periportal hepatocytes (HybHPs) positive for both Sox9 and HNF4α, which have been reported to participate in liver regeneration, but not HCC (Font-Burgada et al., 2015). Therefore, only cells positive for Sox9 but negative for HNF4α were tracked in this study. Moreover, Sox9 induction due to hepatocyte toxicity was definitely excluded.

Crosses of hURI-tetOFF^{hep} and Sox9$^{IRES\text{-}CreERT2}$; R26-stop-EYFP lines showed that at 12 weeks of age, SPCs gave rise to periportal hepatocytes expressing HNF4α and EYFP in control mice (Figures 3J and S3A), suggesting that SPCs supply new periportal hepatocytes (Carpentier et al., 2011). In 12-week-old mutants, newly formed hepatocytes (HNF4α and EYFP positive) invaded the parenchyma (Figure 3J). At 35 weeks of age, we observed EYFP-positive anisokaryotic hepatocyte clusters in the liver periportal region (Figure S3B). Notably, in mutant mice, EYFP-positive hepatocytes derived from SPCs expanded to the parenchyma, and those reaching the central vein expressed cytochrome P450 2E1, indicating that SPCs produce fully differentiated and metabolically functional hepatocytes (Figure S3C). In 65-week-old mice, some tumors were completely EYFP positive (8%), suggesting SPC origin, but in some, EYFP signals were heterogeneous (46%) or non-detectable (46%) (Figures 3K and 3L, tumors 1–3). Thus, SPCs are not the only cells at the origin of liver cancer.

Hepatocytes Contribute to Liver Tumorigenesis

The above observations, together with previous findings showing that genotoxic stress in hepatocytes initiates hepatocarcinogenesis in hURI-tetOFF^{hep} and DEN-treated mice (Tummala et al., 2014), suggest that some tumors in these models may derive from hepatocytes. Thus, we followed hepatocyte fate during hepatocarcinogenesis using mice obtained by crossing serum albumin (SA)^{CreERT2} and R26-stop-EYFP reporter lines (Tummala et al., 2014). In this context, Cre recombinase activation enables expression of EYFP in all hepatocyte progeny. Recombination efficiency was verified after tamoxifen treatment (Figure 4A). IHC revealed that the EYFP signal was restricted to all hepatcoytes (Figure 4B). No signal was observed in DCs or HPCs (Figure 4B). Co-IF of EYFP and the hepatocytic marker HNF4α revealed a recombination efficiency of 99.95% ± 0.05% (Figures 4C and 4D), suggesting almost complete hepatocyte labeling.

Next, we crossed hURI-tetOFF^{hep} and SA^{CreERT2}; R26-stop-EYFP lines (hereafter "crosses") (Figure 4E). At 65 weeks of age, immunofluorescence (IF) of liver sections from crosses revealed that the whole tissue (and tumors) was EYFP-positive

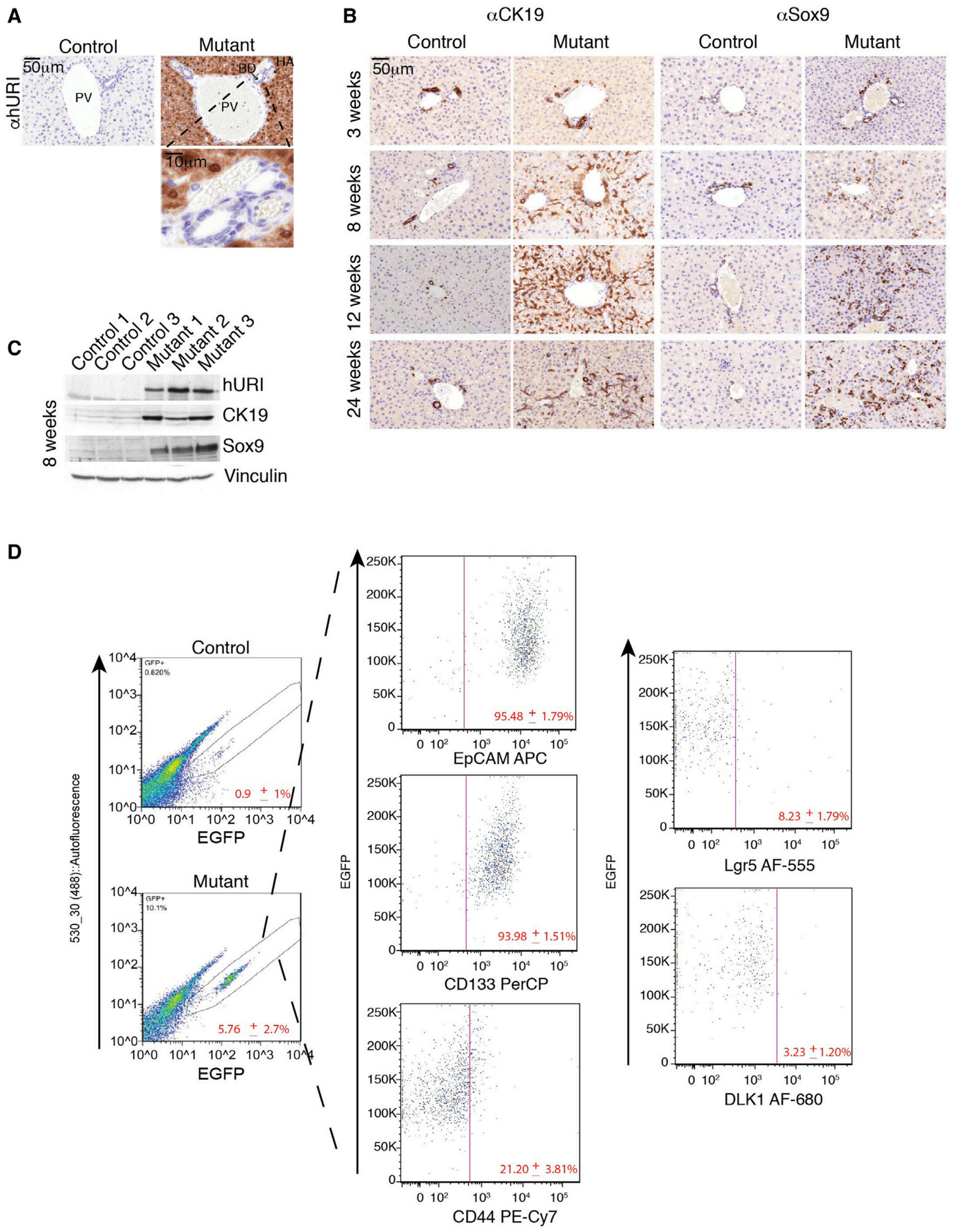

Figure 2. HPCs Expand in the Early Stages of Hepatocarcinogenesis

(A) IHC of 1-week-old hURI-tetOFF^{hep} mouse livers using an antibody recognizing specifically hURI. HA, hepatic artery; BD, bile duct; PV, portal vein.

(B) Sox9 and CK19 IHC in liver sections derived from 3-, 8-, 12-, and 24-week-old hURI-tetOFF^{hep} mice.

(C) Western blot (WB) of liver lysates from 8-week-old hURI-tetOFF^{hep} mice. Membranes were blotted with the indicated antibodies.

(D) FACS of EGFP-positive cells isolated from hURI-tetOFF^{hep} mouse crossed with Sox9$^{IRES-EGFP}$ line. SPCs (EGFP positive) were then analyzed for expression of the indicated markers (EpCAM, CD133, CD44, Lgr5, and DLK1) (n = 6).

Scale bars represent 50 μm and 10 μm.

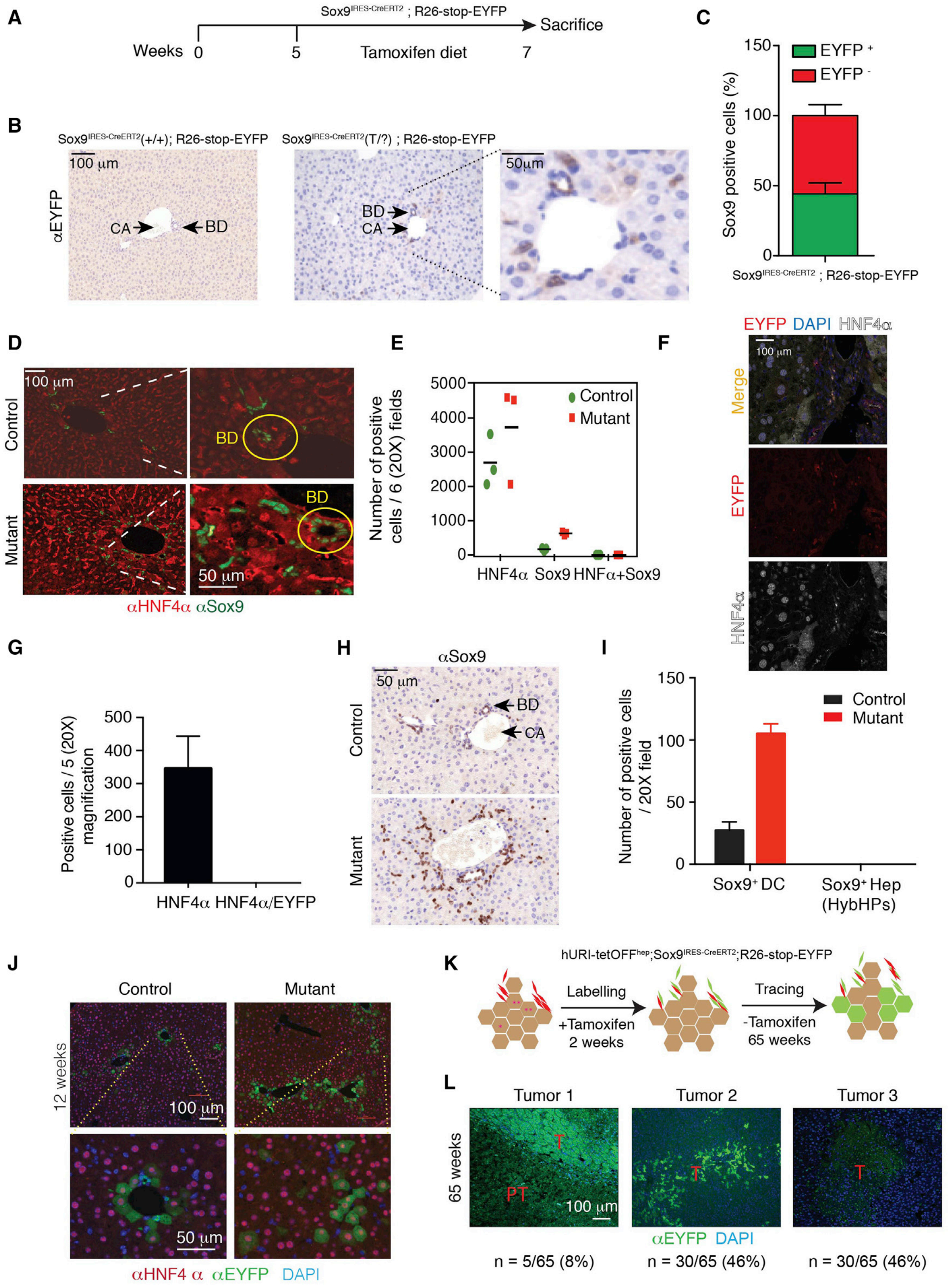

Figure 3. HPCs Contribute to Liver Tumorigenesis

(A) Schematic representation of tamoxifen-treated Sox9$^{\text{IRES-creERT2}}$; R26-stop-EYFP. 5-week-old mice were fed with tamoxifen diet for 2 weeks and then sacrificed at 7 weeks of age.

(B) EYFP IHC in liver sections derived from 7-week-old Sox9$^{\text{IRES-creERT2}}$; R26-stop-EYFP mice treated as described in (A) (n = 3).

(C) Quantification of co-IF of Sox9 and EYFP in mice treated as described in (A) (n = 3).

(legend continued on next page)

(Figure 4F, tumor 1; 17% of all cases), suggesting that hepatocytes contribute to hepatocarcinogenesis. However, in some cases, while peritumoral counterparts were EYFP positive, tumors were highly heterogeneous mixtures of EYFP-positive and EYFP-negative hepatocytes (Figure 4F, tumor 2; 49% of all cases), some were even completely EYFP-negative (Figure 4F, tumor 3; 34% of all cases). We detected similar trends in DEN-treated 35-week-old $SA^{CreERT2}$; R26-stop-EYFP mice (Figures S4A and S4B). Notably, recombination efficiency in DEN-treated $SA^{CreERT2}$; R26-stop-EYFP mice was about 100% (98.1% ± 1.6%) (Figures S4C and S4D). Therefore, hepatocytes participate in liver tumorigenesis but are not the exclusive origin of liver tumors.

We also checked whether EYFP and Sox9 costained in 12-week-old $hURI\text{-}tetOFF^{hep}$ and $SA^{CreERT2}$; R26-stop-EYFP mice. No co-IF was detected in liver sections from crosses, indicating that hepatocyte dedifferentiation may not give rise to SPCs (Figures S4E and S4F).

HCC Predominantly Originates from Hepatocytes, and HPCs Contribute to Liver Tumor Heterogeneity

To determine the contributions of hepatocytes and HPCs to HCC and/or HCA, we evaluated the proportions and types of tumors they predominantly formed. Tumors derived from $SA^{CreERT2}$; R26-stop-EYFP or $Sox9^{IRES\text{-}CreERT2}$; R26-stop-EYFP crosses were first histopathologically classified as either HCC or HCA according to World Health Organization criteria (WHO, 2008). Interestingly, hepatocytes and HPCs participate in liver tumor heterogeneity, giving rise to RNs, HCAs, and HCCs (Figures 5A and S5A–S5C). Normalization of number of tumors to the percentage of recombination indicated that 66% of EYFP-positive tumors originated from labeled hepatocytes. Interestingly, 69% and 31% of EYFP-positive tumors (66% of total) derived from hepatocytes were HCC and HCA, respectively (Figure 5B). Half of the EYFP-negative tumors were HCC and half were HCA, suggesting that they have distinct cell origins, possibly some from SPCs. Notably, 73% and 27% of all HCCs analyzed from $SA^{CreERT2}$; R26-stop-EYFP crosses originated from hepatocytes and non-hepatocytic cells, respectively (Figure 5C). Almost similar percentages of the HCAs originated from hepatocytic and non-hepatocytic cells (Figure 5C). Thus, hepatocytes principally contribute to HCCs.

Most EYFP-positive lesions (54% of total) derived from HPCs were benign (Figure 5D), including dysplasia (9%), RNs (63%), and HCA (14%). Surprisingly, only 14% HCCs were detected, possibly originating directly from HPCs or transiting from the RNs or adenomas known to be HCC precursors (Pilati et al., 2014). Notably, 56% of examined EYFP-negative tumors halved into HCC and benign lesions, including dysplasia (7%), RNs (17%) and HCA (23%) (Figure 5D), suggesting that these EYFP-negative tumors may derive from hepatocytes and/or Sox9-negative cells. Thus, HPCs contribute to liver tumorigeneity (Figure S5C).

In order to evaluate the aggressiveness of the lesions across different tumor types and to further classify HCC variants obtained from hepatocyte and HPC tracing, resected tumors were further analyzed by IHC, a "HAGCKS" (HSP70, AFP, GS, CK19, Ki67, and Sox9) score with arbitrary units was created to classify tumors aggressiveness, as previously reported (Gomes et al., 2016) (Supplemental Experimental Procedures; Figures S5D–S5V). Fundamentally, aggressive tumors irrespective of their origin have high HAGCKS scores. Expectedly, HCCs had the highest score, whereas HCAs, dysplasia, and RNs scored lower (Figures 5E and 5F). Notably, dysplasia and RNs had apparently a higher HAGCKS score than HCAs, suggesting that these lesions in URI mice are HCC precursors (Figure 5F). Histopathology indicated that HCCs emerging from hepatocytes were heterogeneous and resembled the spectrum of primary human HCC subtypes. Trabecular (41%), mixed (33%), glandular (19%), and solid HCCs (7%) emanated from hepatocyte tracing (Figures 5G). Only trabecular HCCs originated from HPCs (Figure 5G). Interestingly, solid HCCs had a higher HAGCKS score than other HCC variants (Figure 5H), suggesting that HCCs originating from hepatocytes may be slightly more aggressive than HCCs derived from HPCs. Therefore, in the URI model, which recapitulates several stages of human hepatocarcinogenesis, hepatocytes and HPCs contribute to liver tumor heterogeneity.

Furthermore, hepatocytes and HPCs were tracked in other hepatocarcinogenesis models described previously (Figure S5C) (Mu et al., 2015). Hepatocytes tracking in R26-mTOM-stop-mGFP mice infected with $AAV8\text{-}Tgb^{Cre}$ and treated with DEN and CCl_4 showed that hepatocytes were the HCC cell of origin (Figures 5E and S5C). This was confirmed in Mdr2 knockout ($Mdr2^{KO}$) mice crossed with R26-stop-ZsGreen line and infected with $AAV8\text{-}Tgb^{Cre}$ (Figures 5E and S5C). All tumors obtained in these mice were HCCs GFP positive from trabecular and mixed types (Figure S5W). However, HCAs or other lesions were not detected (Figures 5E and S5C).

Surprisingly, tracking of HPCs in $Sox9^{IRES\text{-}CreERT2}$; R26-stop-EYFP mice treated with DEN or in $CK19^{IRES\text{-}CreERT2}$; R26-mTOM-stop-mGFP mice treated with DEN and CCl_4 revealed

(D) Co-IF of Sox9 and HNF4α in $Sox9^{IRES\text{-}creERT2}$; R26-stop-EYFP mice. n = 3. BD, bile duct.
(E) Quantification of (D) (n = 3).
(F) Representative images of confocal microscopy of EYFP and HNF4α co-IF in 4-week old $Sox9^{IRES\text{-}creERT2}$; R26-stop-EYFP mice.
(G) Quantification of (F) (n = 5 mice).
(H) Sox9 IHC in liver sections derived from 12-week-old $hURI\text{-}tetOFF^{hep}$ mice (n = 5).
(I) Quantification of (G) (n = 5).
(J) Co-IF of HNF4α and EYFP in liver sections derived from 12-week-old $hURI\text{-}tetOFF^{hep}$; $Sox9^{IRES\text{-}creERT2}$; R26-stop-EYFP mice (n = 5).
(K) Schematic representation of tamoxifen-treated $hURI\text{-}tetOFF^{hep}$; $Sox9^{IRES\text{-}creERT2}$; R26-stop-EYFP mice. 5-week-old mice were fed with tamoxifen diet for 2 weeks and sacrificed at 65 weeks of age.
(L) Representative immunofluorescence images of three different EYFP staining patterns observed in tumors from $hURI\text{-}tetOFF^{hep}$; $Sox9^{IRES\text{-}creERT2}$; R26-stop-EYFP mice treated as described in (K). T, tumor; PT, peritumor; n = 65 tumors.
Data are presented as mean ± SEM. Scale bars represent 100 μm and 50 μm.

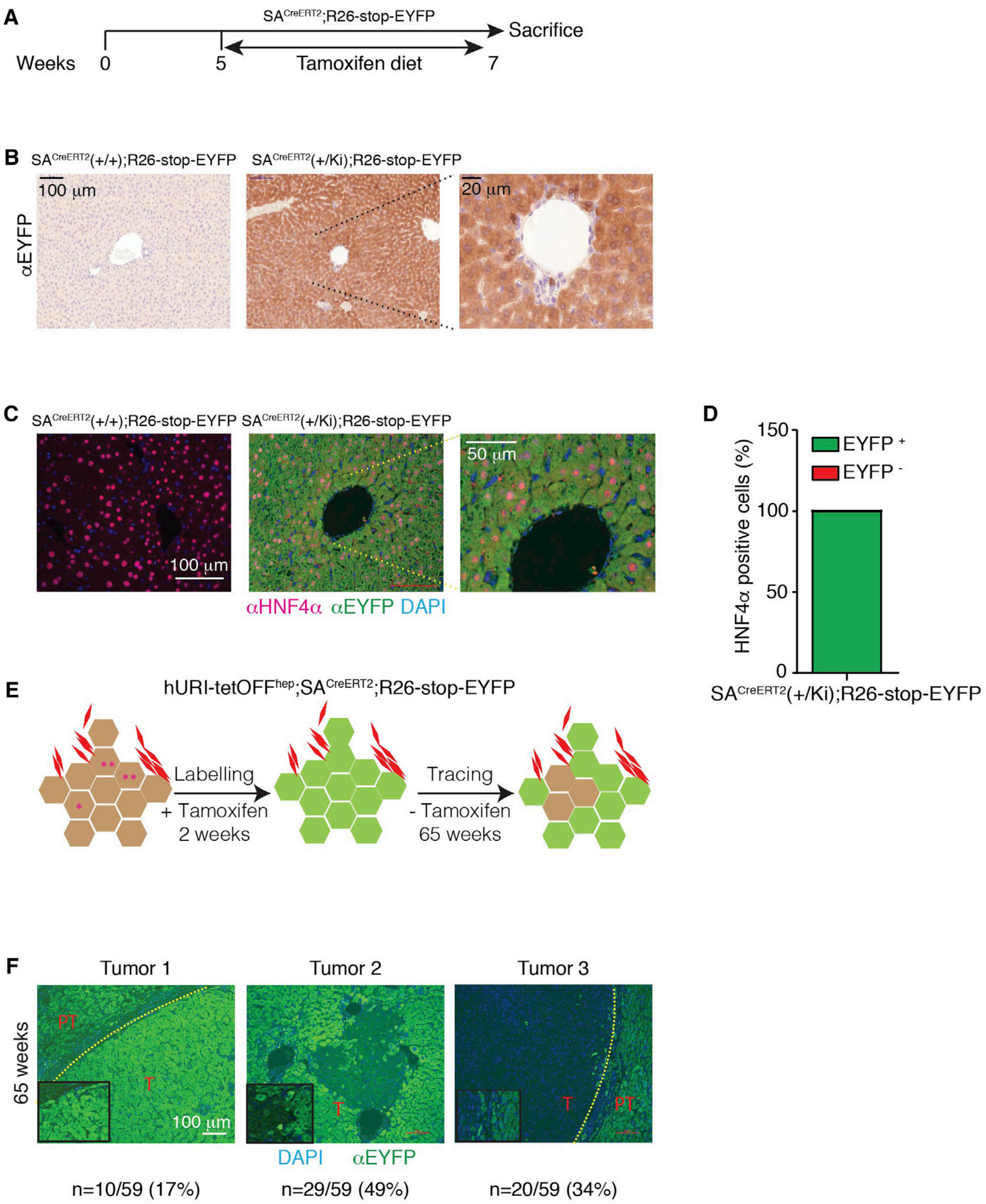

Figure 4. Hepatocytes Contribute to Liver Tumorigenesis

(A) Schematic representation of tamoxifen-treated $SA^{creERT2}$; R26-stop-EYFP mice. 5-week-old mice were fed with tamoxifen diet for 2 weeks and sacrificed at 7 weeks of age.

(B) Representative images of EYFP IHC performed in liver sections derived from $SA^{creERT2}$; R26-stop-EYFP mice treated as described in (A) (n = 3).

(C) Co-IF images of HNF4α and EYFP in liver sections derived from mice treated as described in (A) (n = 3).

(D) Quantification of percentage of hepatocytes positive for both HNF4α and EYFP, as described in (C) (n = 3).

(E) Schematic representation of tamoxifen-treated hURI-$tetOFF^{hep}$; $SA^{creERT2}$; R26-stop-EYFP mice. 5-week-old mice were treated for 2 weeks with tamoxifen and sacrificed at 65 weeks of age.

(F) Representative IF images of three different EYFP staining pattern observed in tumors from hURI-$tetOFF^{hep}$; $SA^{creERT2}$; R26-stop-EYFP mice treated as described in (E); n = 16 mice and 59 tumors.

Scale bars represent 100 μm, 50 μm, and 20 μm.

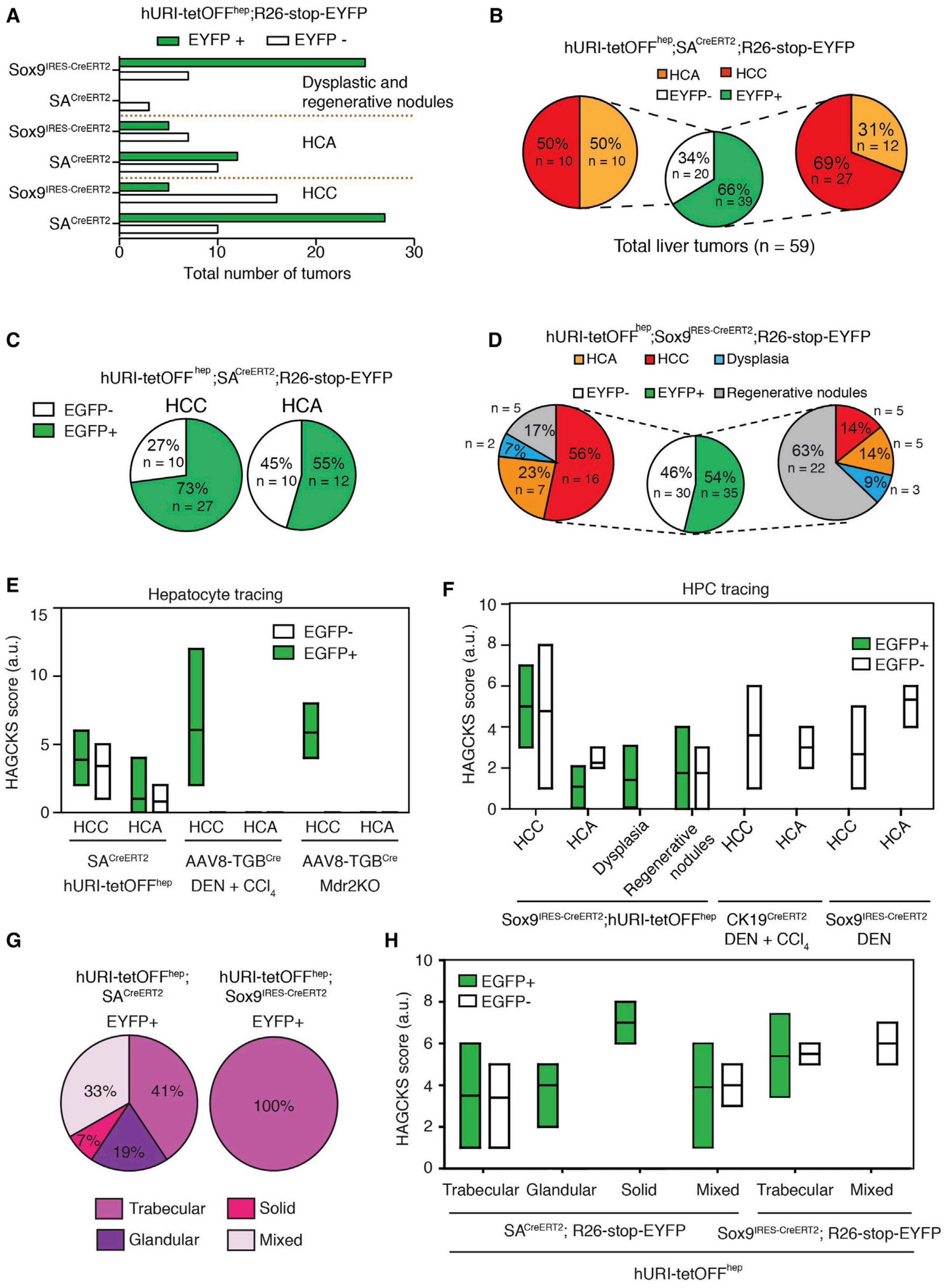

Figure 5. HCC Predominantly Originates from Hepatocytes, and HPCs Contribute to Liver Tumor Heterogeneity

(A) Graph representing normalized number of EGFP-positive and EGFP-negative dysplastic and RNs, HCAs, and HCCs derived from hURI-tetOFF^{hep} crossed with SA^{CreERT2}; R26-stop-EYFP and hURI-tetOFF^{hep} crossed with Sox9^{CreERT2}; R26-stop-EYFP.

(B) Central pie chart representing total liver tumors derived from hURI-tetOFF^{hep} crossed with SA^{CreERT2}; R26-stop-EYFP. Right pie chart represents the percentage of HCCs and HCAs derived from hepatocytes. Left pie chart represents the percentage of HCCs and HCAs derived from the non-hepatocytic population.

(legend continued on next page)

that all HCCs and HCAs obtained were EYFP or GFP negative (Figures 5F and S5C), suggesting that tumors do not originate from HPCs in these models and confirming that tamoxifen treatment of Sox9$^{IRES-CreERT2}$; R26-stop-EYFP mice does not lead to Sox9 expression in hepatocytes (Carpentier et al., 2011). Thus, different liver cancer mouse models, possibly due to the type of damage (oncogene expression, location, and duration) and their capacity to mimic the human disease may have different impact on liver tumorigenesis.

Hepatocytes Expressing Oncogenic URI Produce Galectin-3 to Instruct and Activate HPCs

The above findings suggest that transformed hepatocytes (e.g., oncogenic URI expression) may non-cell-autonomously instruct and determine the fate of HPCs toward oncogenesis. HPCs expand during tumorigenesis and when liver damage impairs hepatocyte turnover. hURI expression leads to NAD$^+$-depletion-induced DNA damage, triggering hepatocyte damage and apoptosis via the p53-DNA damage response pathway (Tummala et al., 2014). Thus, relieving the p53-dependent proliferative break in hepatocytes would stop HPC expansion. In order to verify this, we inactivated p53 in hURI-tetOFF^{hep} mice by crossing them with p53ER^{TAM} mice, in which p53 activation requires ectopic 4-hydroxytamoxifen (Tummala et al., 2014). Surprisingly, while p53 was inactivated, IHC and WB showed that HPC expansion and increased Ki67 was independent of p53 (Figures S6A–S6E). p19ARF was also markedly expressed in hURI-tetOFF^{hep} liver sections (Tummala et al., 2014). However, p19ARF (*Cdkn2a*) deletion did not affect HPC expansion and increased Ki67 in hURI-tetOFF^{hep} mice (Figures S6A–S6D and S6F), suggesting that HPCs expand independently of hepatocytes' proliferative status.

We next checked several inflammation-regulated factors described to induce oval cell expansion. mRNA levels of cytokines were not altered (Figure S6G). Additionally, levels of TWEAK (tumor necrosis factor-like weak inducer of apoptosis) did not change in early tumorigenesis stages (Figure S6H) (Jakubowski et al., 2005), and WNT/β catenin target genes were not upregulated in HPCs from hURI-tetOFF^{hep} and Sox9$^{IRES-EYFP}$ crosses (Figure S6I). Finally, treating 3-week-old hURI-tetOFF^{hep} mice with anti-inflammatory sulindac did not abolish oval cell expansion (Figures S6J–S6L). Thus, inhibition of HPC expansion is independent of inflammatory cues in our model.

Switching off hURI expression with doxycycline significantly reduced HPC proliferation (Figures 6A and 6B), suggesting that continuous hURI expression is essential for oval cell expansion. Abrogation of DNA damage by supplying the NAD$^+$ booster nicotinamide riboside (NR) (Tummala et al., 2014) reduced HPC proliferation in 8-week-old mice (Figures 6C–6E), suggesting that the NAD$^+$-deficit-induced DNA damage axis is essential for oval cell expansion.

Previous iTRAQ analysis (Tummala et al., 2014) revealed that galectin-1 and galectin-3 were highly upregulated in 8-week-old hURI-expressing livers (Figure S6M). Galectins are extracellular β-galactoside-binding lectin, which bind to glycoproteins such as laminin and integrins (also expressed in mutant livers; Figures S2A, S2D, and S2E), to regulate and remodel the ECM and promote integrin signaling and fibrillogenesis, allowing HPC expansion during chronic liver injury (Hsieh et al., 2015). WB confirmed that galectin-3 was strikingly enhanced in 12-week-old mutant livers, but galectin-1 was only modestly increased (Figure S6N). WB and IF of 8-week-old hURI-tetOFF^{hep} livers confirmed that galectin-3 was upregulated in hepatocytes (Figures 6E and S6O). Abrogation of DNA damage by NR reduced galectin-3 levels (Figure 6E), suggesting that hepatocytic NAD$^+$-deficit-induced DNA damage may be involved in galectin-3 secretion. Suppression of glycosylation-dependent surface glycoprotein by 4-fluoro-*N*-acetylgalactosamine (4-F-GalNAc), which inhibits the actions of galectin, significantly reduced HPC expansion in hURI-tetOFF^{hep} livers (Figures S6P–S6R). Thus, transformed hepatocytes are the source of galectin-3, which is essential for HPC expansion and tumorigenesis, highlighting a crosstalk between hepatocytes and HPCs.

To further test galectin-3-mediated activation of HPCs by transformed hepatocytes, mouse bipotential murine oval liver (BMOL) cells were cultured with conditioned media from AML-12 (alpha mouse liver 12) cells (nontumorigenic murine hepatocytes) stably expressing either EGFP or hemagglutinin (HA) URI. A concomitant knockdown of galectin-3 in AML-12 cells overexpressing HA-URI significantly reduced BMOL cell numbers (Figures 6F–6H). Similar results were obtained when hepatocytes were transformed by oncogenic c-MYC (data not

(C) Left pie chart represents the total number of HCCs positive or negative for EYFP and derived from hURI-tetOFF^{hep} crossed with SA^{CreERT2}; R26-stop-EYFP mice. Right pie chart represents the total number of liver HCAs positive or negative for EYFP and derived from hURI-tetOFF^{hep} crossed with SA^{CreERT2}; R26-stop-EYFP mice.

(D) Central pie chart representing normalized number of liver tumors derived from hURI-tetOFF^{hep} crossed with Sox9$^{IRES-CreERT2}$; R26-stop-EYFP mice. Right pie chart represents the normalized percentage of liver lesions derived from HPCs. Left pie chart represents the percentage of liver lesions derived from a distinct population than HPCs.

(E) HAGCKS score for EGFP-positive and EGFP-negative HCCs or HCAs obtained after hepatocyte tracing in hURI-tetOFF^{hep} crossed with SA^{CreERT2}; R26-stop-EYFP mice (n = 59), R26-mTOM-stop-mGFP mice infected with AAV8-Tgb^{Cre} and treated with DEN and CCl_4 (n = 42), and Mdr2^{KO} mice crossed with R26-stop-ZsGreen mice and infected with AAV8-Tgb^{Cre} (n = 7).

(F) HAGCKS score for EGFP-positive and EGFP-negative HCC, HCA, dysplasia or RNs obtained after HPC tracing in hURI-tetOFF^{hep} crossed with Sox9^{CreERT2}; R26-stop-EYFP mice (n = 65), CK19^{CreERT2}; R26-mTOM-stop-mGFP mice treated with DEN and CCl_4 (n = 8), and Sox9^{CreERT2}; R26-stop-EYFP mice treated with DEN (n = 6).

(G) Pie charts representing type of HCC derived from hepatocytes in SA^{CreERT2}; R26-stop-EYFP mice (left chart), or HPCs in Sox9$^{IRES-CreERT2}$; R26-stop-EYFP mice (right chart).

(H) HAGCKS score for EGFP-positive and EGFP-negative HCCs derived from hURI-tetOFF^{hep} crossed with SA^{CreERT2}; R26-stop-EYFP mice (n = 59), and hURI-tetOFF^{hep} crossed with Sox9^{CreERT2}; R26-stop-EYFP mice (n = 65) and plotted according to tumor type.

Data are presented as mean ± SEM.

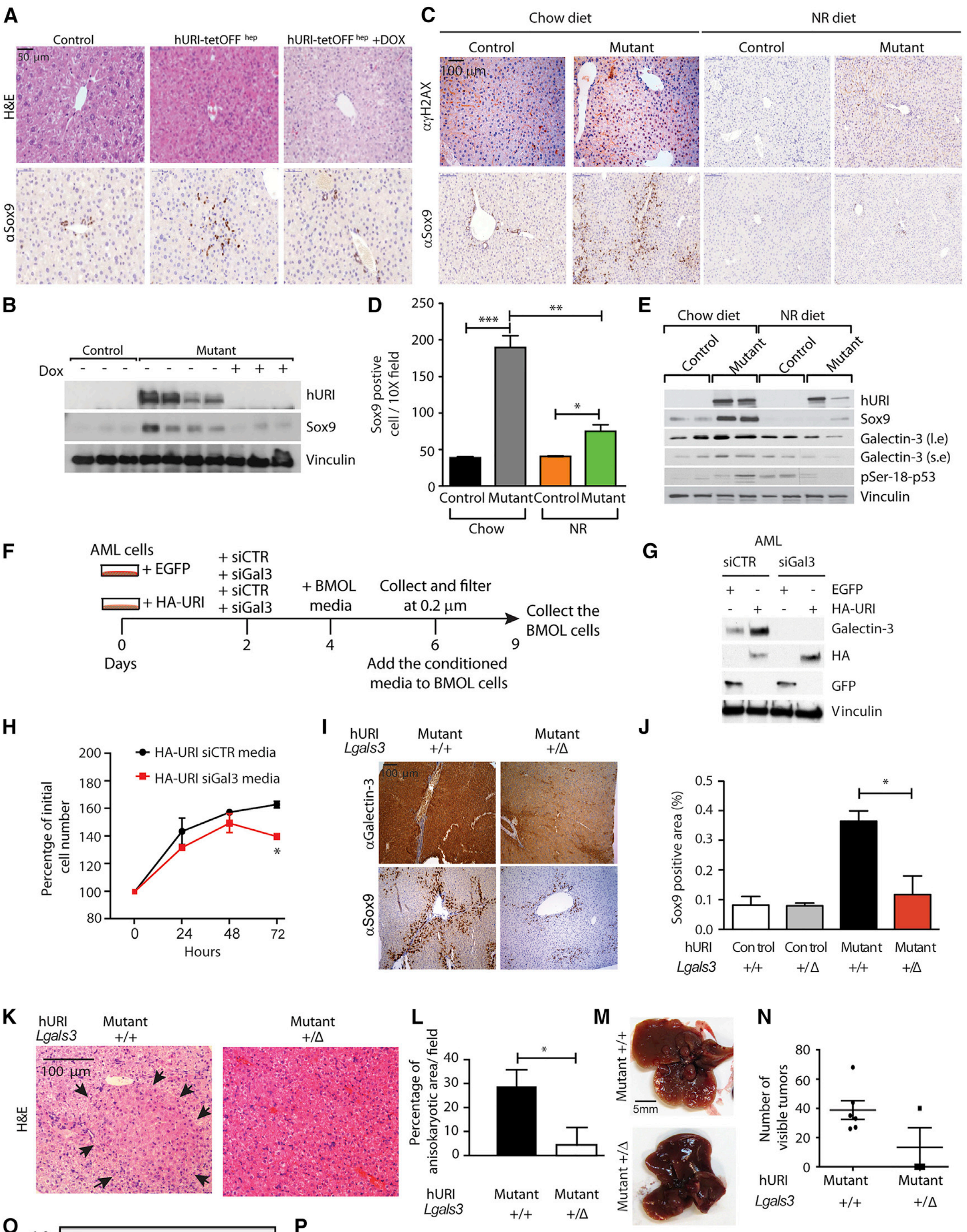

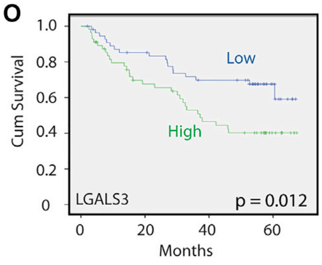

P

-2 log Likelihood	Overall (score)			Change from previous step			Change from previous Block		
	Chi-square	df	Sig.	Chi-square	df	Sig.	Chi-square	df	Sig.
853.935	7.169	2	0.028	7.204	2	0.027	7.204	2	0.027

(legend on next page)

shown), which is known to be implicated in hepatocarcinogenesis (Burén et al., 2016), suggesting that transformed hepatocytes, through DNA damage, talk to HPCs via galectin-3 secretion.

Next, hURI-tetOFF^{hep} mice crossed with heterozygous galectin-3 knockout mice had significantly reduced HPCs and dysplastic lesions and number of hepatic tumors (Figures 6I–6N). Notably, levels of galectin-3 were significantly much higher in the URI model than in DEN-treated mice (Figures S6S and S6T). Galectin-3 is thus determinant for HPC expansion and their tumorigenic fate in the hURI-tetOFF^{hep}, but not in DEN-treated mice.

To estimate the clinical relevance of our findings, we interrogated the galectin family gene signature using gene expression dataset from a cohort of 221 HCC patients (GEO: GSE14520) (Roessler et al., 2010). Multivariate Cox regression indicated that the overall survival of HCC patients was significantly associated with *LGALS3*, *LGALS4*, and *LGALS7* gene signatures (p = 0.025) (Figure S6U), but not with all combined *LGALS* genes (data not shown). Importantly, *LGALS3* gene expression positively correlated with patient poor prognosis (Figure 6O), whereas *LGALS4* and *LGALS7* gene expression did not show significant differences (Figures S6V and S6W). Furthermore, the multivariate Cox regression suggested a significant association between *URI* (*C19orf2*) or *c-MYC* and *LGALS3* expression and predicted poor patient survival (p = 0.027 for *URI* and p = 0.023 for *c-MYC*) (Figures 6P and S6X). Thus, galectin-3 activates HPC, negatively affecting overall patient survival.

Hepatocytes Expressing Oncogenic URI Produce α-Ketoglutarate and Galectin-3 to Preserve an HPC Undifferentiated State

To probe signaling changes that may explain why HPCs do not immediately differentiate into hepatocytes while proliferating, we further analyzed our previously reported iTRAQ data (Tummala et al., 2014). Several glycolysis enzymes, including hexokinase (HK1), glyceraldehyde-3-phosphate dehydrogenase, and pyruvate kinase, were upregulated in early tumorigenesis in hURI-tetOFF^{hep} hepatocytes (Figures 7A and S7A), suggesting a pseudo-Warburg-like effect. Accordingly, pyruvate dehydrogenase kinase, which inactivates pyruvate dehydrogenase (catalyzing pyruvate to acetyl-coenzyme A conversion), was downregulated (Figures 7A and S7A). Many other enzymes involved in the tricarboxylic acid (TCA) cycle acting downstream of α-ketoglutarate were also downregulated (Figures 7A and S7A). Thus, glycolytic flux may result in α-ketoglutarate accumulation, maintaining the undifferentiated state of HPCs in the liver (Carey et al., 2015; Saha et al., 2014).

In accordance with our iTRAQ data, α-ketoglutarate was significantly elevated in mutant livers (Figure 7B) but decreased following NR treatment, suggesting that hepatocytic NAD$^+$-depletion-induced DNA damage is essential for α-ketoglutarate elevation (Figure 7B). Further, 4-F-GalNAc treatment did not affect the α-ketoglutarate levels (Figure 7C), suggesting that α-ketoglutarate accumulation is independent of changes in glycan structures. Treatment of BMOL cells with dimethyl 2-oxoglutarate (or dimethyl-α-ketoglutarate), a precursor for the glutamine synthesis, significantly increased expression of stem cell markers such as *Cd44, Sox9*, or *Epcam*, but expression of *Hnf4a* was reduced, as shown by qRT-PCR (Figure S7B). This suggests that increased α-ketoglutarate maintains HPC undifferentiated state. Addition of dimethyl succinate and dimethyl 2-oxoglutarate further enhanced the expression of stem cells markers (Figure S7B), while dimethyl succinate favored an intermediate state, confirming previous work and showing that the α-ketoglutarate/succinate ratio suppresses stem cell differentiation (Carey et al., 2015). Additionally, media from AML-12 cells stably expressing HA-URI and treated

Figure 6. Hepatocytes Expressing Oncogenic URI Produce Galectin-3 to Instruct and Activate HPCs

(A) Representative images of H&E and Sox9 staining in liver sections derived from 8-week-old hURI-tetOFF^{hep} mice fed with either chow or doxycycline (Dox) diet as previously described (Tummala et al., 2014).

(B) WB of liver extracts from 8-week-old hURI-tetOFF^{hep} mice fed with either a chow or Dox diet as previously described (Tummala et al., 2014). Membranes were blotted with the indicated antibodies.

(C) Representative images of γH2AX and Sox9 IHC in liver sections derived from 8-week-old hURI-tetOFF^{hep} mice fed with either chow or nicotinamide riboside (NR) diet (Tummala et al., 2014).

(D) Quantification of Sox9 staining from (C) (n = 4).

(E) WB of liver extracts from 8-week-old hURI-tetOFF^{hep} mice fed with either chow or NR diet. Membranes were blotted with the indicated antibodies. l.e., long exposure; s.e., short exposure.

(F) Schematic representation of AML-12 and BMOL cell treatment. Media from EGFP- or HA-URI-overexpressing AML-12 cells transfected either with siCTR or siGal3 was used to culture BMOL cells for 3 days.

(G) WB of AML-12 cells from (F). Membranes were blotted with the indicated antibodies.

(H) Number of BMOL cells after 0, 24, 48, and 72 hr of incubation with media from AML-12 cells from (F).

(I) Representative images of Sox9 and galectin-3 IHC in liver sections derived from 8-week-old mutant; Lgals3$^{+/+}$ and mutant; Lgals3$^{+/\Delta}$ mice.

(J) Quantification of Sox9 IHC from (I). n = 4 for control; Lgals3$^{+/+}$ and mutant; Lgals3$^{+/+}$ and n = 3 for control; Lgals3$^{+/\Delta}$ and for control; Lgals3$^{+/\Delta}$.

(K) Representative images of H&E in liver sections derived from 10-week-old mutant(KI/KI); Lgals3$^{+/+}$ and mutant(KI/KI); Lgals3$^{+/\Delta}$ mice. Black arrows denote anisokaryotic area.

(L) Quantification of anisokaryotic area from (K) (n = 3).

(M) Representative images of livers derived from 10-week-old mutant(KI/KI); Lgals3$^{+/+}$ and mutant(KI/KI); Lgals3$^{+/\Delta}$ mice.

(N) Quantification of number of visible tumors in livers from (M). n = 6 for mutant(KI/KI); Lgals3$^{+/+}$ and n = 3 for mutant(KI/KI); Lgals3$^{+/\Delta}$.

(O) Kaplan-Meier curve of human HCC patient cumulative survival based on the expression of *LGALS3.* Degrees of freedom = 1; chi-square = 6.243; p = 0.012.

(P) Multivariate Cox regression survival for *LGALS3* and *URI1* in 221 patient human HCC gene expression analyses. (p = 0.027). "df" and "Sig." represents degrees of freedom and significance, respectively.

Data are presented as mean ± SEM. *p ≤ 0.05; **p ≤ 0.01; ***p ≤ 0.001. Scale bars represent 5 mm, 100 μm, and 50 μm.

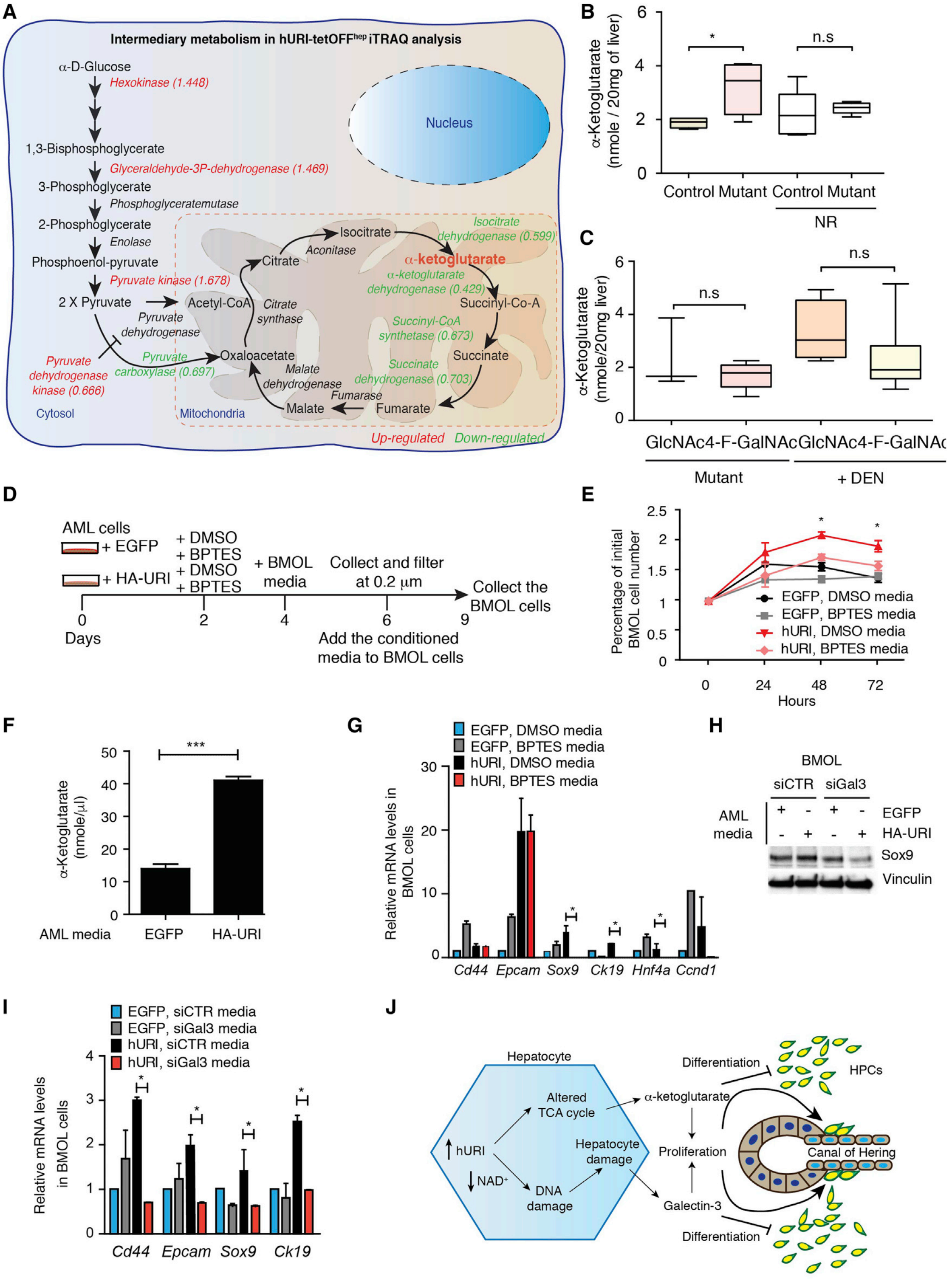

Figure 7. Hepatocytes Expressing Oncogenic URI Produce α-Ketoglutarate and Galectin-3 to Preserve an HPC Undifferentiated State

(A) Schematic representation of intermediary metabolism induced in hURI-tetOFF^{hep} mice. Data were obtained and analyzed from previous iTRAQ data obtained from livers from 1- or 8-week-old hURI-tetOFF^{hep} mice (Tummala et al., 2014). Fold changes are represented within the brackets. Upregulated enzymes are represented in red, and downregulated enzymes are represented in green.

(legend continued on next page)

with bis-2-(5-phenylacetamido-1,3,4-thiadiazol-2-yl)ethyl sulfide (BPTES), a selective inhibitor of glutaminase GLS1 reducing α-ketoglutarate levels, decreased BMOL cell numbers (Figures 7D and 7E) (Xiang et al., 2015). Notably, media from HA-URI-expressing AML-12 cells had higher levels of α-ketoglutarate (Figure 7F). However, in vitro, only few stem cell markers were reduced (Figure 7G), suggesting that other paracrine signals (e.g., galectin-3) emerging from transformed hepatocytes contribute to preserve the HPC stemness.

Multivariate Cox regression of a gene set of intermediate metabolism downregulated in URI model revealed a nonsignificant association between expression of *IDH1*, *IDH2*, *IDH3A*, *IDH3B*, *IDH3G*, *OGDH*, *SCS*, *SDH*, or *PC3* and overall patient survival (Figure S7C). Expression analysis of a single gene from intermediate metabolism also failed to classify the patients with poor prognosis (data not shown). We therefore checked whether galectin-3 was sufficient to sustain the undifferentiated state of HPCs. BMOL cells cultured with media from AML-12 cells expressing HA-URI or His-c-MYC and depleted for galectin-3 showed a significant reduction of all stem cell markers (Figures 7H, 7I, and S7D–S7G). Galectin-3 secreted by transformed hepatocytes may thus play a prominent role in maintaining HPC stemness, expansion, and aggressiveness (Figure 7J).

DISCUSSION

Different murine models, possibly due to their capacity of mimicking the human disease or the type of damage (oncogene expression, location, and duration) impact differently on tumorigenesis. In the hURI-tetOFF^{hep} mouse model, which recapitulates some features and stages of the human hepatocarcinogenesis (Gomes et al., 2016; Tummala et al., 2014), hepatocytes and HPCs contribute to liver tumor heterogeneity. Supporting this, transplanted HPCs from normal mice regenerate hepatocytes and restore liver parenchyma in mice with hepatocytic deletion of the E3 ubiquitin ligase Mdm2-induced apoptosis, necrosis, and foremost senescence (Lu et al., 2015). Consistently, in the hURI-tetOFF^{hep} model, HPCs clearly supply new mature hepatocytes, even though we do not completely exclude that hepatocytes can also derive from pre-existing hepatocytes (Schaub et al., 2014; Yanger et al., 2014). Interestingly, hepatocytes expressing URI show high levels of replicative stress and undergo into senescence (Tummala et al., 2014), arguing that the type of hepatocyte damage may determine HPC fate during tumorigenesis. HPCs are therefore a potential future alternative to hepatocyte or liver transplantation for liver disease, depending on the damage, and can participate in hepatocarcinogenesis.

Proliferation block or apoptosis of hepatocytes triggers growth of HPCs (Jung et al., 2010). In our study, HPC proliferation seems to depend on the hepatocyte's oncogenic status rather than its proliferative capacity. Transformed hepatocytes instruct the neighboring HPCs through paracrine signal mediated by NAD^{+}-deficit-induced DNA damage, leading to galectin-3 and α-ketoglutarate secretion, to maintain an HPC undifferentiated state and activation. In this regard, galectins are implicated in fibrosis of multiple organs. Specifically, galectin-3 is reportedly activated in cirrhotic liver and HCC (Hsu et al., 1999), and high circulating levels are associated with poor prognosis in patients with primary HCC, corroborating our results (Jiang et al., 2014).

Activated HPCs contribute to the formation of RNs, HCAs, and HCCs. Notably, shared mutations by HPCs and RNs putatively indicate common origins (Lin et al., 2010). Moreover, RNs mainly occur in cirrhotic livers and define histological development of cirrhosis, a potent risk factor for HCC (Lin et al., 2010; Schuppan and Afdhal, 2008). RNs are thus likely HCC precursors. Thus, HPC activation may engender cirrhosis and subsequently, through selective pressure, HCC formation.

HCA is a rare benign liver tumor primarily found in women using hormonal contraceptives. The etiology is uncertain, but direct linkage between estrogen exposure and HCA growth is suspected, and estrogens reportedly contribute to the proliferative responses of biliary epithelial cells (Fouassier et al., 2009). Interestingly, HCC may result from malignant transformation of HCA (Pilati et al., 2014), even though this transformation does not occur in non-fibrotic livers (Schulze et al., 2015). Finally, patients with trabecular HCCs often have NASH (Salomao et al., 2010). Accordingly, hURI-tetOFF^{hep} mice develop NASH and trabecular HCCs (Gomes et al., 2016; Tummala et al., 2014).

Transplantation of DC-induced organoids has been recently proposed to promote liver regeneration, especially for patients with resected HCC (Huch et al., 2013a). Since HPCs give rise to RN-induced cirrhosis, the re-implantation strategy of HPCs from HCC patients should be approached cautiously. Liver transplantation remains the only curative option for some

(B) Histogram representing α-ketoglutarate levels from livers from either NR-treated or untreated 12-week-old hURI-tetOFF^{hep} mice as indicated. n = 6 for controls and mutants; n = 5 for controls and mutants treated with NR.

(C) Histogram representing α-ketoglutarate levels from livers from 4-week-old hURI-tetOFF^{hep} mice (mutant) and DEN-treated C57BL/6 mice. Mice were treated with either GlcNAc or 4-F-GalNAc for 3 weeks. n = 3 for mutants treated with GlcNAc.

(D) Schematic representation of AML-12 and BMOL cell treatment. Media from HA-URI-overexpressing AML-12 cells treated either with DMSO or BPTES (10 μM) was used to culture BMOL cells for 3 days.

(E) Cell number of BMOL cells after 0, 24, 48, and 72 hr of incubation with media from AML-12 cells from (D).

(F) Histogram representing α-ketoglutarate levels in media from AML-12 cells stably overexpressing EGFP or HA-URI.

(G) qRT-PCR of BMOL cells cultured with media from AML-12 cells as described in (D).

(H) WB of BMOL cells after 72 hr incubation with media from AML-12 cells stably overexpressing EGFP or HA-URI and transfected with either siCTR or siGal3. Membrane was blotted with the indicated antibodies.

(I) qRT-PCR of BMOL cells cultured with media from AML-12 cells as described in (H).

(J) Scheme representing hepatocytes expressing oncogenic URI-secreted galectin-3 to induce HPC proliferation, whereas α-ketoglutarate preserves an HPC undifferentiated state during expansion.

Data are presented as mean ± SEM. ns, non significant; $^{*}p \leq 0.05$; $^{***}p \leq 0.001$.

patients, but pharmacological blockage of galectin-3 may offer elegant approaches for inhibiting cirrhosis progression and HCC, thereby reducing needs for transplantation. Finally, HCC is an intractable primary liver cancer, certainly due to the recurrence of multiple secondary liver tumors after surgical interventions or first therapies. HPCs predict recurrence in HCC patients after curative resection (Ye et al., 2014). Our findings may thus provide a disease management strategy after primary surgical resection to circumvent tumor recurrence, facilitating the design of efficient therapies against liver cancer and recurrences.

EXPERIMENTAL PROCEDURES

Antibodies

Materials and antibodies were purchased from different manufacturers as detailed in Supplemental Experimental Procedures.

Generation, Handling, and Treatment of Mice

Mice were generated, handled, and treated as previously described (Gomes et al., 2016; Tummala et al., 2014) and detailed in Supplemental Experimental Procedures. All mice have been housed in pathogen-free conditions. All experiments were approved by the CNIO-ISCIII Ethics Committee and performed in accordance with the guidelines for ethical conduct in the care and use of animals as stated in the international guiding principles for biomedical research involving animals, developed by the Council for International Organizations of Medical Sciences. Littermates were always used as controls. No gender differences were observed and age/developmental stage of mice was included appropriately in the text and the figure legends. Food (Harlan Laboratories and Research Diets Inc.) and water were provided ad libitum.

Cell Culture, Transfection, and Cell Number Counts

Cell culture, transfection, and cell number counts were performed as detailed in Supplemental Experimental Procedures.

Image Analysis

Six images per slide were taken at 10× and 20× magnifications and quantified using color de-convolution, a colocalization finder, and image analysis tools available in Fiji/ImageJ software (http://fiji.sc/).

HAGCKS Score

Consecutive liver sections were stained by immunohistochemistry for HSP70, AFP, GS, CK19, Ki67, and Sox9 (HAGCKS). Differential expression levels (0–3) of GS and HSP70 as well as Sox9 were evaluated using previously reported classifications (Gomes et al., 2016). CK19 and AFP were only classified in positive (1) or negative (0) cases. Individual cases were considered immunoreactive (IR) for individual antigens when more than 5% of cells were IR. IR cases were further sub-classified as follows: 5%–10% IR cells (1), 11%–50% IR cells (2), and more than 50% IR cells (3). HAGCKS score was determined by adding the grading of immunohistochemistry signal intensity for each individual marker and scored in Figure 5.

α-Ketoglutarate Measurement

Total α-ketoglutarate levels were measured according to the manufacturer's instruction provided by a kit purchased from Sigma-Aldrich (MAK054A). For liver tissue, the protocol was scaled up to two times.

Serum TWEAK Measurement

Serum TWEAK levels were measured according to the manufacturer's instructions using a Mouse TWEAK/TNFSF12 ELISA kit purchased from R&D Systems (DY1237).

Flow Cytometry of HPCs

Flow cytometry of HPCs was performed as detailed in Supplemental Experimental Procedures.

Immunoblotting

Immunoblotting was performed as previously described elsewhere (Tummala et al., 2014).

Immunohistochemistry and Immunofluorescence

Immunohistochemistry and immunofluorescence were performed as previously described (Tummala et al., 2014).

Genotyping and qRT-PCR

Genotyping and qRT-PCR (including primer sequences) were performed as detailed in Supplemental Experimental Procedures.

Statistical Analysis

Statistical analyses were performed using GraphPad Prism V5.0 software. Statistical significance (*$p \leq 0.05$, **$p \leq 0.01$, and ***$p \leq 0.001$) between the means of a minimum of three groups was determined using unpaired two-tailed Student's t test or linear regression analysis. Results are expressed as the mean value ± SEM or SD, as indicated. All results, including WB, are representative of at least three independent experiments. Statistical parameter false discovery rate estimates the probability of a gene set with false-positive finding. Normalized enrichment score allows to compare enrichment analysis results across gene sets. Tumor numbers normalization in the $Sox9^{IRES\text{-}CreERT2}$ model was done according to Cre recombination efficiency, and contingency analysis was performed using two-sided Fischer's exact tests (results are displayed in Figures S5A and S5B). Survival and Kaplan-Meier analyses for individual genes were performed using publically available human HCC patient gene expression datasets (GEO: GSE14520) (Roessler et al., 2010). Multivariate Cox regression analyses were performed using SPSS software (version 19).

ACCESSION NUMBERS

The accession number for the FACS data reported in this paper is Flow Repository: FR-FCM-ZY3T.

SUPPLEMENTAL INFORMATION

Supplemental Information includes Supplemental Experimental Procedures and seven figures and can be found with this article online at http://dx.doi.org/10.1016/j.celrep.2017.03.059.

AUTHOR CONTRIBUTIONS

K.S.T. designed performed and analyzed most of the in vivo experiments. M.B. designed performed and analyzed in vitro experiments and experiments with the Gal3KO mice. K.S.T., M.B., A.T., and C.P. performed and analyzed HAGCKS scores. C.P. analyzed all tissues histology. O.G. performed the bioinformatics analyses. R.F.S. provided some paraffin-embedded blocks used in HAGCKS scoring and previously reported (Mu et al., 2015). K.S.T., M.B., and N.D. analyzed all the data. N.D. designed the experiments and conceived, developed, and wrote the project and the manuscript. Funding was secured by N.D.

ACKNOWLEDGMENTS

We thank G. Yeoh and N. Tirnitz-Parker for sharing BMOL cells and X. Sole for helping with statistics. We thank E. Wagner for critical reading of this manuscript. M.B. is a recipient of a La Caixa PhD fellowship. N.D. is a recipient of the Spanish Ramón y Cajal fellowship. This work was supported by grants from the Spanish Ministry of Economy and Competitiveness (SAF2013-46089-R) and AICR-UK (11-0242) (all to N.D.).

Received: November 11, 2015
Revised: February 8, 2017
Accepted: March 21, 2017
Published: April 18, 2017

REFERENCES

Boyault, S., Rickman, D.S., de Reyniès, A., Balabaud, C., Rebouissou, S., Jeannot, E., Hérault, A., Saric, J., Belghiti, J., Franco, D., et al. (2007). Transcriptome classification of HCC is related to gene alterations and to new therapeutic targets. Hepatology *45*, 42–52.

Burén, S., Gomes, A.L., Teijeiro, A., Fawal, M.A., Yilmaz, M., Tummala, K.S., Perez, M., Rodriguez-Justo, M., Campos-Olivas, R., Megías, D., and Djouder, N. (2016). Regulation of OGT by URI in Response to Glucose Confers c-MYC-Dependent Survival Mechanisms. Cancer Cell *30*, 290–307.

Carey, B.W., Finley, L.W., Cross, J.R., Allis, C.D., and Thompson, C.B. (2015). Intracellular α-ketoglutarate maintains the pluripotency of embryonic stem cells. Nature *518*, 413–416.

Carpentier, R., Suner, R.E., van Hul, N., Kopp, J.L., Beaudry, J.B., Cordi, S., Antoniou, A., Raynaud, P., Lepreux, S., Jacquemin, P., et al. (2011). Embryonic ductal plate cells give rise to cholangiocytes, periportal hepatocytes, and adult liver progenitor cells. Gastroenterology *141*, 1432–1438.

Coulouarn, C., Factor, V.M., and Thorgeirsson, S.S. (2008). Transforming growth factor-beta gene expression signature in mouse hepatocytes predicts clinical outcome in human cancer. Hepatology *47*, 2059–2067.

Espanol-Suner, R., Carpentier, R., Van Hul, N., Legry, V., Achouri, Y., Cordi, S., Jacquemin, P., Lemaigre, F., and Leclercq, I.A. (2012). Liver progenitor cells yield functional hepatocytes in response to chronic liver injury in mice. Gastroenterology *143*, 1564–1575.

Font-Burgada, J., Shalapour, S., Ramaswamy, S., Hsueh, B., Rossell, D., Umemura, A., Taniguchi, K., Nakagawa, H., Valasek, M.A., Ye, L., et al. (2015). Hybrid periportal hepatocytes regenerate the injured liver without giving rise to cancer. Cell *162*, 766–779.

Fouassier, L., Rosenberg, P., Mergey, M., Saubaméa, B., Clapéron, A., Kinnman, N., Chignard, N., Jacobsson-Ekman, G., Strandvik, B., Rey, C., et al. (2009). Ezrin-radixin-moesin-binding phosphoprotein (EBP50), an estrogen-inducible scaffold protein, contributes to biliary epithelial cell proliferation. Am. J. Pathol. *174*, 869–880.

Furuyama, K., Kawaguchi, Y., Akiyama, H., Horiguchi, M., Kodama, S., Kuhara, T., Hosokawa, S., Elbahrawy, A., Soeda, T., Koizumi, M., et al. (2011). Continuous cell supply from a Sox9-expressing progenitor zone in adult liver, exocrine pancreas and intestine. Nat. Genet. *43*, 34–41.

Gomes, A.L., Teijeiro, A., Burén, S., Tummala, K.S., Yilmaz, M., Waisman, A., Theurillat, J.P., Perna, C., and Djouder, N. (2016). Metabolic inflammation-associated IL-17A causes non-alcoholic steatohepatitis and hepatocellular carcinoma. Cancer Cell *30*, 161–175.

He, G., Dhar, D., Nakagawa, H., Font-Burgada, J., Ogata, H., Jiang, Y., Shalapour, S., Seki, E., Yost, S.E., Jepsen, K., et al. (2013). Identification of liver cancer progenitors whose malignant progression depends on autocrine IL-6 signaling. Cell *155*, 384–396.

Holczbauer, A., Factor, V.M., Andersen, J.B., Marquardt, J.U., Kleiner, D.E., Raggi, C., Kitade, M., Seo, D., Akita, H., Durkin, M.E., and Thorgeirsson, S.S. (2013). Modeling pathogenesis of primary liver cancer in lineage-specific mouse cell types. Gastroenterology *145*, 221–231.

Hoshida, Y., Nijman, S.M., Kobayashi, M., Chan, J.A., Brunet, J.P., Chiang, D.Y., Villanueva, A., Newell, P., Ikeda, K., Hashimoto, M., et al. (2009). Integrative transcriptome analysis reveals common molecular subclasses of human hepatocellular carcinoma. Cancer Res. *69*, 7385–7392.

Hsieh, W.C., Mackinnon, A.C., Lu, W.Y., Jung, J., Boulter, L., Henderson, N.C., Simpson, K.J., Schotanus, B., Wojtacha, D., Bird, T.G., et al. (2015). Galectin-3 regulates hepatic progenitor cell expansion during liver injury. Gut *64*, 312–321.

Hsu, D.K., Dowling, C.A., Jeng, K.C., Chen, J.T., Yang, R.Y., and Liu, F.T. (1999). Galectin-3 expression is induced in cirrhotic liver and hepatocellular carcinoma. Int. J. Cancer *81*, 519–526.

Huch, M., Boj, S.F., and Clevers, H. (2013a). Lgr5(+) liver stem cells, hepatic organoids and regenerative medicine. Regen. Med. *8*, 385–387.

Huch, M., Dorrell, C., Boj, S.F., van Es, J.H., Li, V.S., van de Wetering, M., Sato, T., Hamer, K., Sasaki, N., Finegold, M.J., et al. (2013b). In vitro expansion of single Lgr5+ liver stem cells induced by Wnt-driven regeneration. Nature *494*, 247–250.

Iizuka, N., Oka, M., Yamada-Okabe, H., Mori, N., Tamesa, T., Okada, T., Takemoto, N., Sakamoto, K., Hamada, K., Ishitsuka, H., et al. (2005). Self-organizing-map-based molecular signature representing the development of hepatocellular carcinoma. FEBS Lett. *579*, 1089–1100.

Jakubowski, A., Ambrose, C., Parr, M., Lincecum, J.M., Wang, M.Z., Zheng, T.S., Browning, B., Michaelson, J.S., Baetscher, M., Wang, B., et al. (2005). TWEAK induces liver progenitor cell proliferation. J. Clin. Invest. *115*, 2330–2340.

Jiang, S.S., Weng, D.S., Wang, Q.J., Pan, K., Zhang, Y.J., Li, Y.Q., Li, J.J., Zhao, J.J., He, J., Lv, L., et al. (2014). Galectin-3 is associated with a poor prognosis in primary hepatocellular carcinoma. J. Transl. Med. *12*, 273.

Jörs, S., Jeliazkova, P., Ringelhan, M., Thalhammer, J., Dürl, S., Ferrer, J., Sander, M., Heikenwalder, M., Schmid, R.M., Siveke, J.T., and Geisler, F. (2015). Lineage fate of ductular reactions in liver injury and carcinogenesis. J. Clin. Invest. *125*, 2445–2457.

Jung, Y., Witek, R.P., Syn, W.K., Choi, S.S., Omenetti, A., Premont, R., Guy, C.D., and Diehl, A.M. (2010). Signals from dying hepatocytes trigger growth of liver progenitors. Gut *59*, 655–665.

Lee, J.S., Chu, I.S., Heo, J., Calvisi, D.F., Sun, Z., Roskams, T., Durnez, A., Demetris, A.J., and Thorgeirsson, S.S. (2004). Classification and prediction of survival in hepatocellular carcinoma by gene expression profiling. Hepatology *40*, 667–676.

Liao, Y.L., Sun, Y.M., Chau, G.Y., Chau, Y.P., Lai, T.C., Wang, J.L., Horng, J.T., Hsiao, M., and Tsou, A.P. (2008). Identification of SOX4 target genes using phylogenetic footprinting-based prediction from expression microarrays suggests that overexpression of SOX4 potentiates metastasis in hepatocellular carcinoma. Oncogene *27*, 5578–5589.

Lin, W.R., Lim, S.N., McDonald, S.A., Graham, T., Wright, V.L., Peplow, C.L., Humphries, A., Kocher, H.M., Wright, N.A., Dhillon, A.P., and Alison, M.R. (2010). The histogenesis of regenerative nodules in human liver cirrhosis. Hepatology *51*, 1017–1026.

Lu, W.Y., Bird, T.G., Boulter, L., Tsuchiya, A., Cole, A.M., Hay, T., Guest, R.V., Wojtacha, D., Man, T.Y., Mackinnon, A., et al. (2015). Hepatic progenitor cells of biliary origin with liver repopulation capacity. Nat. Cell Biol. *17*, 971–983.

Malato, Y., Naqvi, S., Schürmann, N., Ng, R., Wang, B., Zape, J., Kay, M.A., Grimm, D., and Willenbring, H. (2011). Fate tracing of mature hepatocytes in mouse liver homeostasis and regeneration. J. Clin. Invest. *121*, 4850–4860.

Mu, X., Español-Suñer, R., Mederacke, I., Affò, S., Manco, R., Sempoux, C., Lemaigre, F.P., Adili, A., Yuan, D., Weber, A., et al. (2015). Hepatocellular carcinoma originates from hepatocytes and not from the progenitor/biliary compartment. J. Clin. Invest. *125*, 3891–3903.

Pilati, C., Letouzé, E., Nault, J.C., Imbeaud, S., Boulai, A., Calderaro, J., Poussin, K., Franconi, A., Couchy, G., Morcrette, G., et al. (2014). Genomic profiling of hepatocellular adenomas reveals recurrent FRK-activating mutations and the mechanisms of malignant transformation. Cancer Cell *25*, 428–441.

Roessler, S., Jia, H.L., Budhu, A., Forgues, M., Ye, Q.H., Lee, J.S., Thorgeirsson, S.S., Sun, Z., Tang, Z.Y., Qin, L.X., and Wang, X.W. (2010). A unique metastasis gene signature enables prediction of tumor relapse in early-stage hepatocellular carcinoma patients. Cancer Res. *70*, 10202–10212.

Rountree, C.B., Mishra, L., and Willenbring, H. (2012). Stem cells in liver diseases and cancer: recent advances on the path to new therapies. Hepatology *55*, 298–306.

Saha, S.K., Parachoniak, C.A., Ghanta, K.S., Fitamant, J., Ross, K.N., Najem, M.S., Gurumurthy, S., Akbay, E.A., Sia, D., Cornella, H., et al. (2014). Mutant IDH inhibits HNF-4α to block hepatocyte differentiation and promote biliary cancer. Nature *513*, 110–114.

Salomao, M., Yu, W.M., Brown, R.S., Jr., Emond, J.C., and Lefkowitch, J.H. (2010). Steatohepatitic hepatocellular carcinoma (SH-HCC): a distinctive

histological variant of HCC in hepatitis C virus-related cirrhosis with associated NAFLD/NASH. Am. J. Surg. Pathol. *34*, 1630–1636.

Schaub, J.R., Malato, Y., Gormond, C., and Willenbring, H. (2014). Evidence against a stem cell origin of new hepatocytes in a common mouse model of chronic liver injury. Cell Rep. *8*, 933–939.

Schulze, K., Imbeaud, S., Letouzé, E., Alexandrov, L.B., Calderaro, J., Rebouissou, S., Couchy, G., Meiller, C., Shinde, J., Soysouvanh, F., et al. (2015). Exome sequencing of hepatocellular carcinomas identifies new mutational signatures and potential therapeutic targets. Nat. Genet. *47*, 505–511.

Schuppan, D., and Afdhal, N.H. (2008). Liver cirrhosis. Lancet *371*, 838–851.

Tummala, K.S., Gomes, A.L., Yilmaz, M., Graña, O., Bakiri, L., Ruppen, I., Ximénez-Embún, P., Sheshappanavar, V., Rodriguez-Justo, M., Pisano, D.G., et al. (2014). Inhibition of de novo NAD(+) synthesis by oncogenic URI causes liver tumorigenesis through DNA damage. Cancer Cell *26*, 826–839.

Wang, X., Foster, M., Al-Dhalimy, M., Lagasse, E., Finegold, M., and Grompe, M. (2003). The origin and liver repopulating capacity of murine oval cells. Proc. Natl. Acad. Sci. USA *100* (*Suppl 1*), 11881–11888.

WHO (World Health Organization) (2008). Pathology and genetics of tumors of the digestive system. S.R. Hamilton and L.A. Aaltonen, eds. (IARC Press).

Woo, H.G., Park, E.S., Cheon, J.H., Kim, J.H., Lee, J.S., Park, B.J., Kim, W., Park, S.C., Chung, Y.J., Kim, B.G., et al. (2008). Gene expression-based recurrence prediction of hepatitis B virus-related human hepatocellular carcinoma. Clin. Cancer Res. *14*, 2056–2064.

Xiang, Y., Stine, Z.E., Xia, J., Lu, Y., O'Connor, R.S., Altman, B.J., Hsieh, A.L., Gouw, A.M., Thomas, A.G., Gao, P., et al. (2015). Targeted inhibition of tumor-specific glutaminase diminishes cell-autonomous tumorigenesis. J. Clin. Invest. *125*, 2293–2306.

Xu, X., Liu, R.F., Zhang, X., Huang, L.Y., Chen, F., Fei, Q.L., and Han, Z.G. (2012). DLK1 as a potential target against cancer stem/progenitor cells of hepatocellular carcinoma. Mol. Cancer Ther. *11*, 629–638.

Yamashita, T., Ji, J., Budhu, A., Forgues, M., Yang, W., Wang, H.Y., Jia, H., Ye, Q., Qin, L.X., Wauthier, E., et al. (2009). EpCAM-positive hepatocellular carcinoma cells are tumor-initiating cells with stem/progenitor cell features. Gastroenterology *136*, 1012–1024.

Yanger, K., Knigin, D., Zong, Y., Maggs, L., Gu, G., Akiyama, H., Pikarsky, E., and Stanger, B.Z. (2014). Adult hepatocytes are generated by self-duplication rather than stem cell differentiation. Cell Stem Cell *15*, 340–349.

Ye, F., Jing, Y.Y., Guo, S.W., Yu, G.F., Fan, Q.M., Qu, F.F., Gao, L., Yang, Y., Wu, D., Meng, Y., et al. (2014). Proliferative ductular reactions correlate with hepatic progenitor cell and predict recurrence in HCC patients after curative resection. Cell Biosci. *4*, 50.

Zender, L., Xue, W., Cordón-Cardo, C., Hannon, G.J., Lucito, R., Powers, S., Flemming, P., Spector, M.S., and Lowe, S.W. (2005). Generation and analysis of genetically defined liver carcinomas derived from bipotential liver progenitors. Cold Spring Harb. Symp. Quant. Biol. *70*, 251–261.

Cell Reports, Volume *19*

Supplemental Information

Hepatocellular Carcinomas Originate Predominantly from Hepatocytes and Benign Lesions from Hepatic Progenitor Cells

Krishna S. Tummala, Marta Brandt, Ana Teijeiro, Osvaldo Graña, Robert F. Schwabe, Cristian Perna, and Nabil Djouder

SUPPLEMENTAL INFORMATION

Supplemental Data

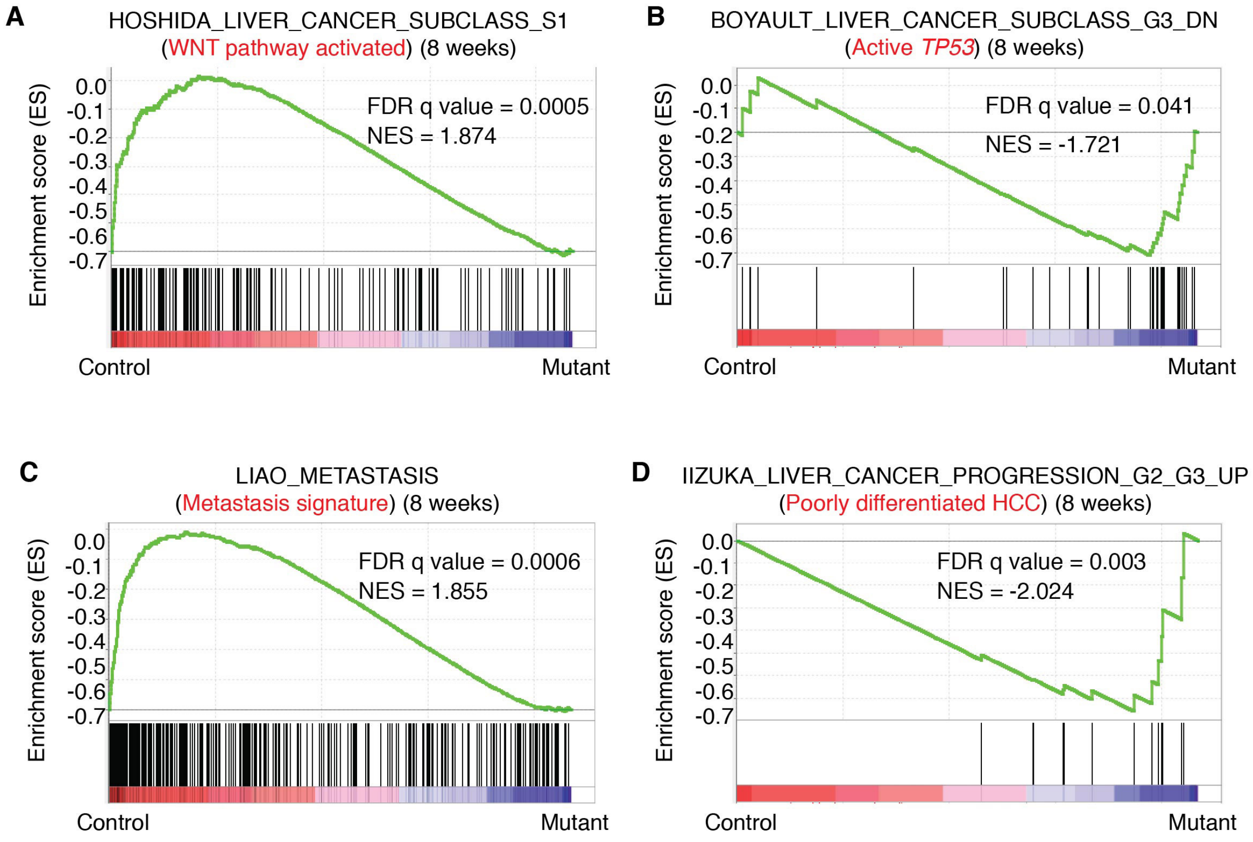

Figure S1. hURI-tetOFF^{hep} Mice Display HPC Signatures in Early Hepatocarcinogenesis Stages Correlating with Aggressive Human HCC, Related to Figure 1

(A-D) GSEA of human HCC gene sets and 8-week-old hURI-tetOFF^{hep} mice data sets previously analyzed (Tummala et al., 2014).

Normalized enrichment scores (NES) and false discovery rates (FDR) q-value are indicated in each graph.

A

S.No	Protein name	Symbol	Fold change		Rank	
			1 week	8 weeks	1 week	8 weeks
1	Keratin, type1 cytoskeletal 19	Krt19	1.058	6.567	647	1
2	Transgelin-2	Tagln2	0.810	2.599	2262	9
3	Creatine kinase B-type	Ckb	1.007	2.377	1179	14
4	Transgelin	Tagln	0.818	2.107	2262	29
5	Vimentin	Vim	1.016	2.022	1078	38
6	Translationally-controlled tumor protein	Tpt	1.306	1.873	38	54
7	Laminin subunit gamma-1	Lamc1	1.133	1.767	249	67
8	Annexin A3	Anxa3	1.060	1.650	629	87
9	Keratin, type II cytoskeletal 7 OS	Krt7	1.218	1.608	87	96
10	Heterogeneous nuclear ribonucleoprotein A1	Hnmpa1	0.985	1.474	1048	137
11	Heterogeneous nuclear ribonucleoprotein A/B	Hnmpab	1.029	1.353	932	208

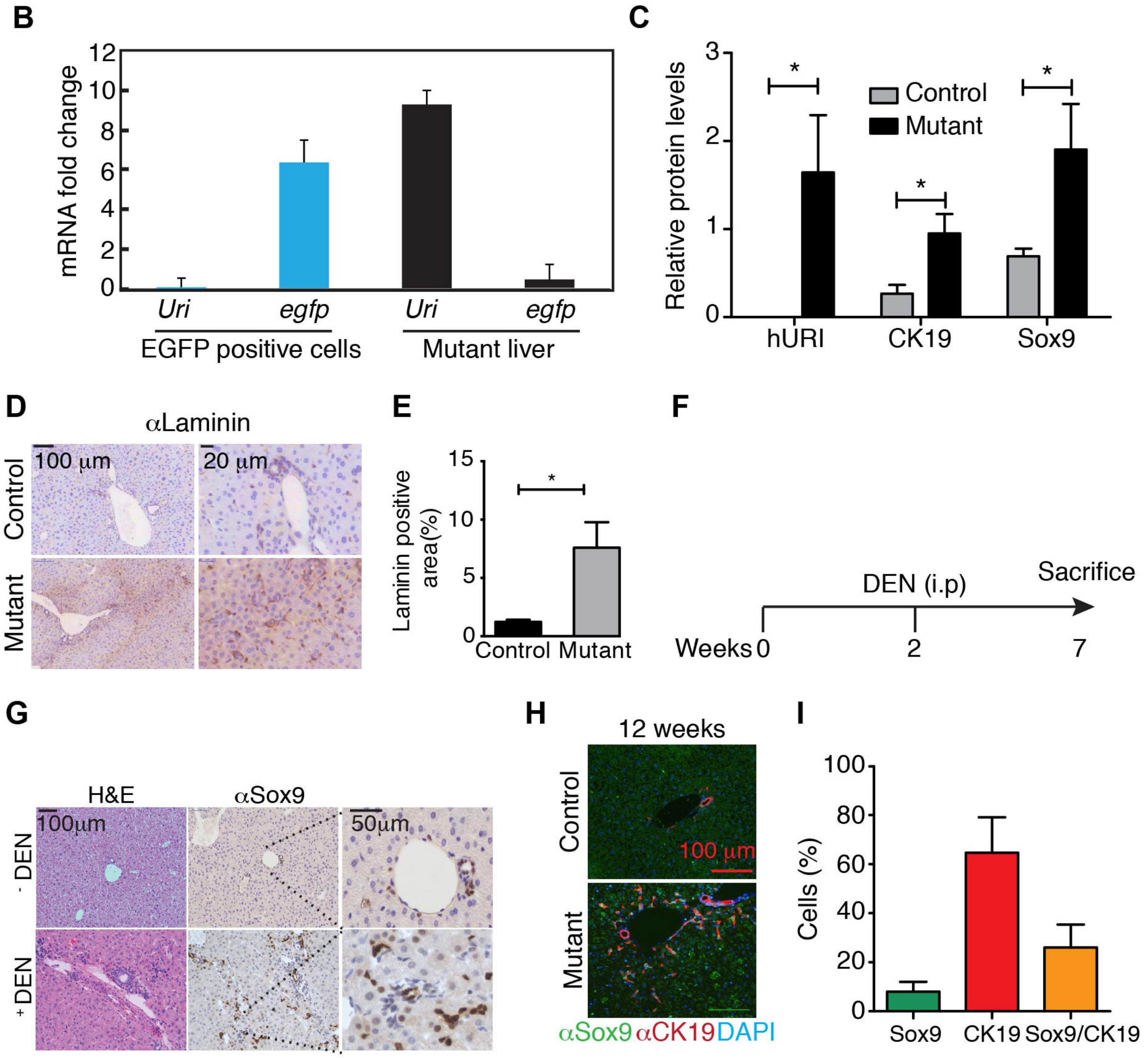

Figure S2. HPCs Expand in the Early Stages of Hepatocarcinogenesis, Related to Figure 2

(A) iTRAQ data from 1- and 8-week old hURI-tetOFF^{hep} previously described (Tummala et al., 2014). Fold change represents the ratio of protein levels between control and mutant livers. Rank represents the ranking position of differentially expressed proteins.

(B) qRT-PCR analysis of FACS-sorted Sox9/EGFP-positive cells from 12-week-old hURI-tetOFF^{hep} mice crossed with Sox9$^{IRES\text{-}EGFP}$ line. Error bars represent ± SD.

(C) Quantification of WB represented in Figure 2C.

(D) IHC analysis of laminin in liver sections from 12-week-old hURI-tetOFF^{hep} mice.

(E) Quantification of (D).

(F) Schematic representation of diethylnitrosamine (DEN)-treated C57BL/6 mice. Mice were injected intraperitoneally with one single dose of 25 mg/kg of DEN at 2 weeks of age and were sacrificed at 7 weeks of age.

(G) Representative pictures of H&E staining and Sox9 IHC of liver sections derived from 7-week-old C57BL/6 mice treated as described in (F).

(H) Co-immunofluorescence of Sox9 and CK19 in liver sections from 12-week-old hURI-tetOFF^{hep} mice.

(I) Quantification of (H).

Unless otherwise indicated data represented as mean ± SEM.

Scale bar represents 20 μm, 50 μm and 100 μm. $p \leq 0.05$ = *

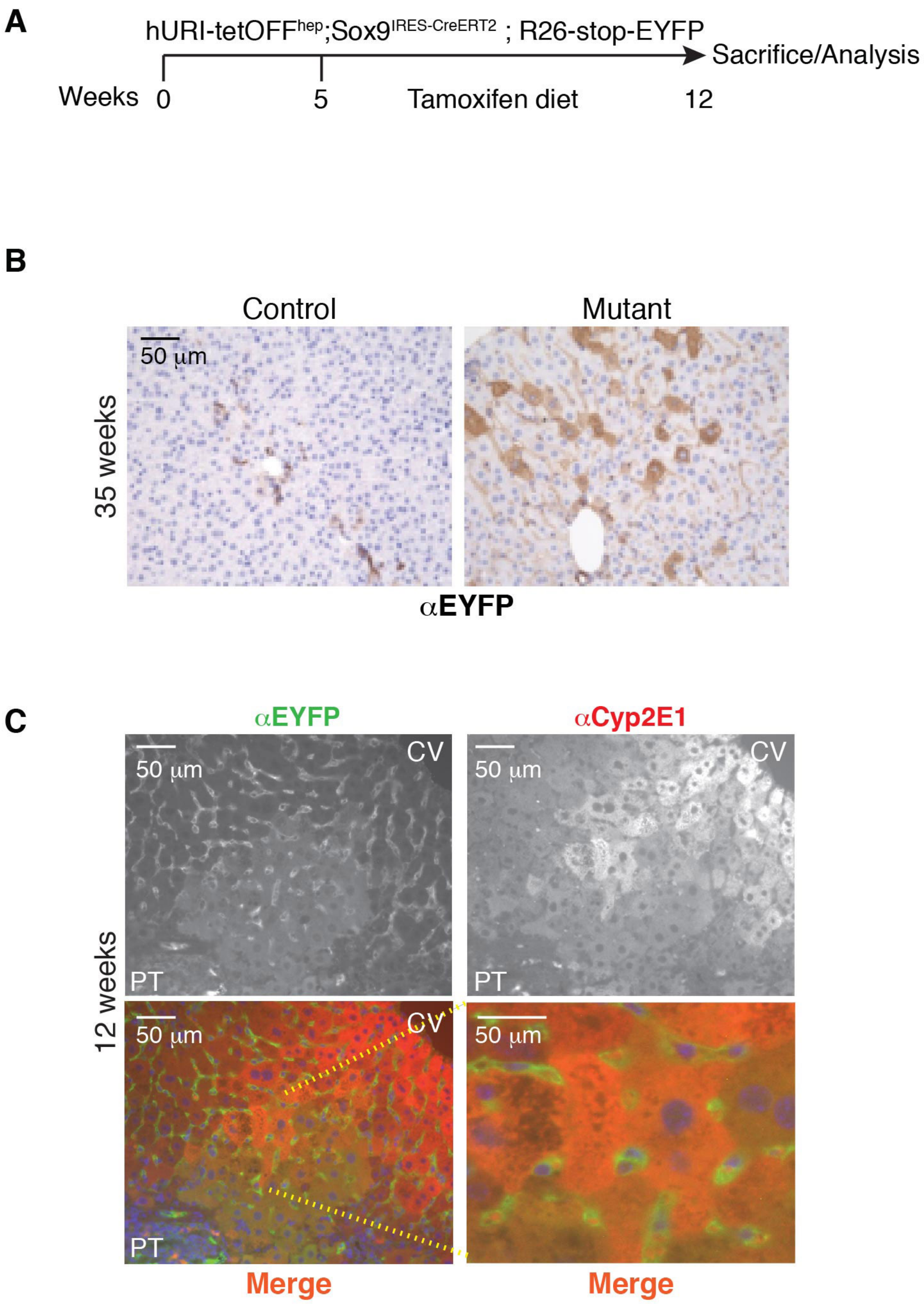

Figure S3. HPCs Contribute to Liver tumorigenesis, Related to Figure 3

(A) Scheme representing tamoxifen-treated hURI-tetOFF^{hep} ; Sox9$^{IRES\text{-}creERT2}$; R26-stop-EYFP mice.

(B) Representative images of EYFP stained by IHC in liver sections from 35-week-old hURI-tetOFF^{hep} ; Sox9$^{IRES\text{-}creERT2}$; R26-stop-EYFP mice.

(C) Co-immunofluorescence of EYFP and Cyp2E1 from mice described in (B).

Scale bars represent 50 μm.

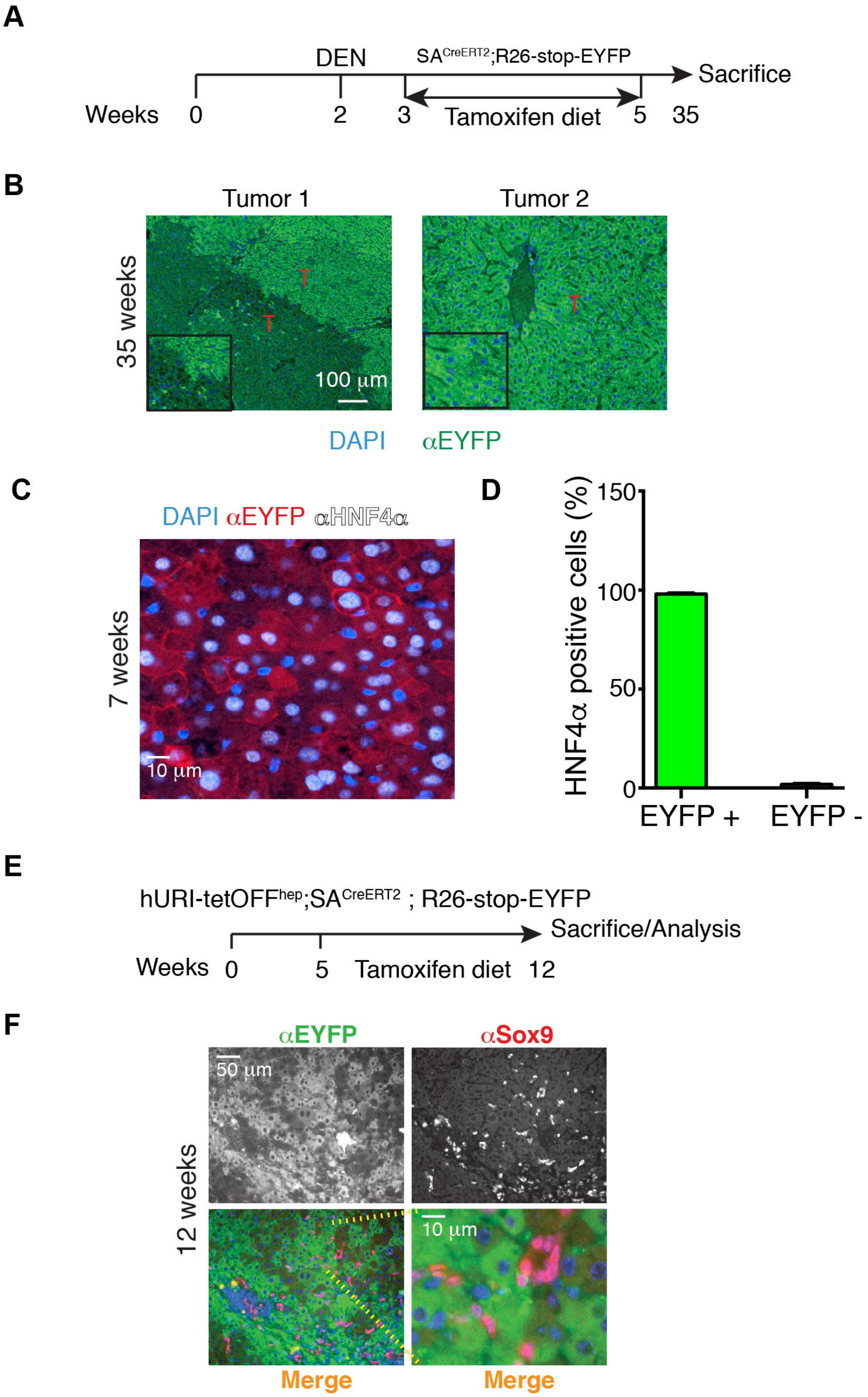
A
DEN
SA^CreERT2;R26-stop-EYFP
Sacrifice
Weeks 0 2 3 Tamoxifen diet 5 35
B
Tumor 1
Tumor 2
35 weeks
T
100 μm
DAPI αEYFP
C
DAPI αEYFP αHNF4α
7 weeks
10 μm
D
HNF4α positive cells (%)
150
100
50
0
EYFP +
EYFP -
E
hURI-tetOFF^hep;SA^CreERT2 ; R26-stop-EYFP
Sacrifice/Analysis
Weeks 0 5 Tamoxifen diet 12
F
αEYFP
αSox9
50 μm
12 weeks
10 μm
Merge
Merge

Figure S4. Hepatocytes Contribute to Liver Tumorigenesis, Related to Figure 4

(A) Schematic representation of DEN- and tamoxifen-treated 35-week-old $SA^{CreERT2}$; R26-stop-EYFP mice.

(B) Representative immunofluoresence images of EYFP staining in tumors obtained from DEN-treated $SA^{CreERT2}$; R26-stop-EYFP mice as described in (A). n = 10 tumors.

(C) Co-immunofluoresence analysis of HNF4α and EYFP in liver sections from DEN- and tamoxifen-treated 7-week old $SA^{CreERT2}$; R26-stop-EYFP mice. n = 3.

(D) Quantification of HNF4α and EYFP co-localisation from (C). n = 3.

(E) Schematic representation of tamoxifen treatment in hURI-$tetOFF^{hep}$; $SA^{CreERT2}$; R26-stop-EYFP mice.

(F) Co-immunofluoresence analysis of Sox9 and EYFP in liver sections from 12-week-old hURI-$tetOFF^{hep}$ mice. n = 5.

Scale bars represent 10 μm, 50 μm and 100 μm

A

Liver pathology		HCC		HCA		Dysplasia + Regeneration	
Normalized	EYFP	Negative	Positive	Negative	Positive	Negative	Positive
	$SA^{CreERT2}$ (to 99.95%)	10	27	10	12	3	0
	$Sox9^{IRESCreERT2}$ (to 44.2%)	16	5	7	5	7	25

B

Normalized	P value (Fisher's test)	P value (Summary)	Relative Risk	Reciprocal Relative Risk	Odds ratio	Reciprocal Odds ratio
HCC	0.0004	***	0.3547	2.819	0.1157	8.64
HCA	0.7207	ns	0.7792	1.283	0.5952	1.68
Dysplasia + Regeneration	0.0183	*	4.571	0.2188	~	0

C

Cell type tracked	Model of tumorigenesis	Tracking system	Number of tumors
HPC	hURI-tetOFF^{hep}	$Sox9^{IRES-creERT2}$; R26-stop-EYFP	65
	DEN	$Sox9^{IRES-creERT2}$; R26-stop-EYFP	6
	DEN and CCl_4	$CK19^{IRES-CreERT2}$; R26-mTOM-stop-mGFP	8
Hepatocyte	hURI-tetOFF^{hep}	$SA^{CreERT2}$; R26-stop-EYFP	59
	DEN	$SA^{CreERT2}$; R26-stop-EYFP	10
	DEN and CCl4	AAV8-Tgb^{Cre} ; R26-mTOM-stop-mGFP	42
	$Mdr2^{KO}$	AAV8-Tgb^{Cre} ; R26-stop-ZsGreen	7

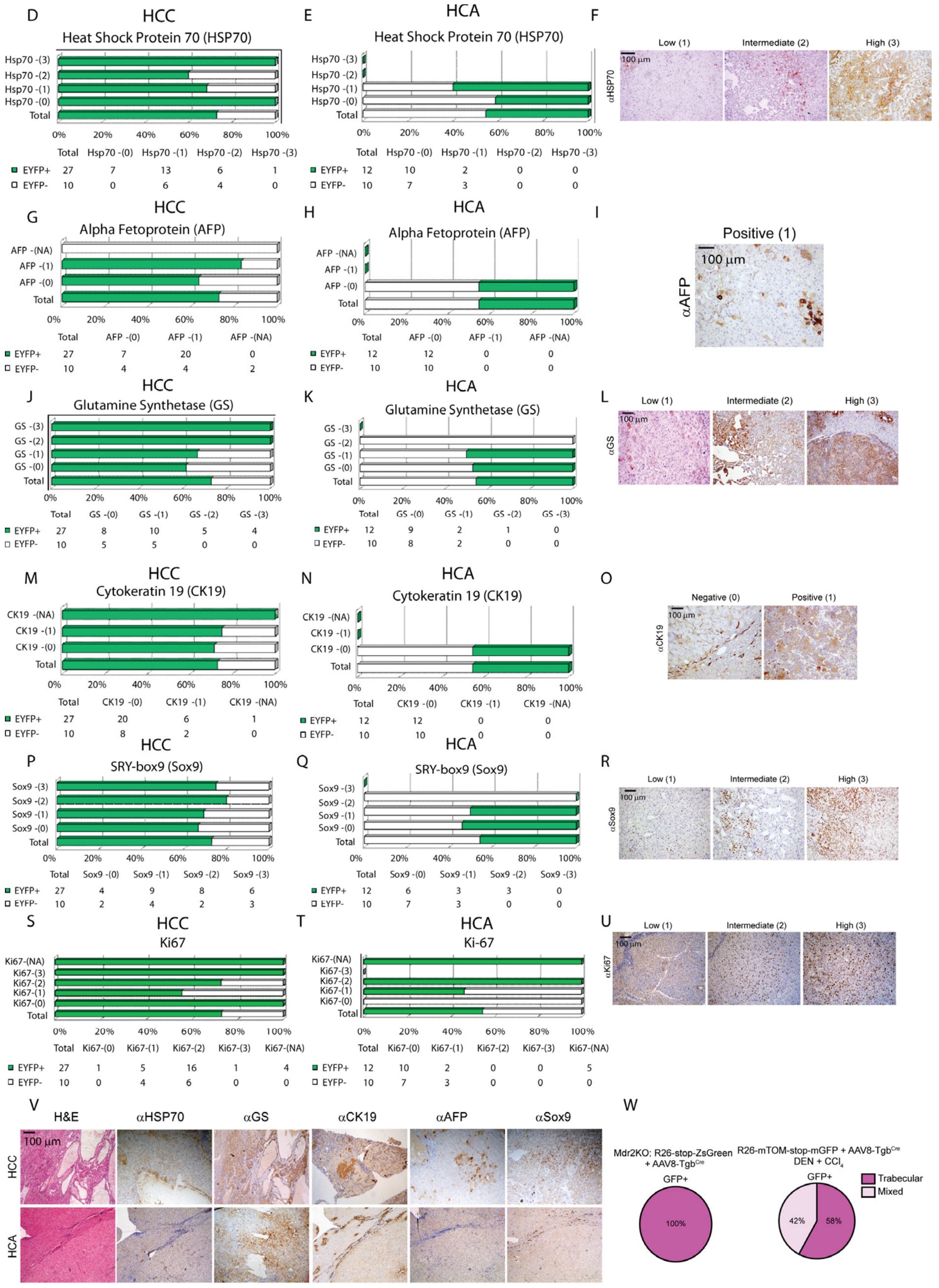

Figure S5. HCC Predominantly Originates From Hepatocytes and HPCs Contribute to Liver Tumor Heterogeneity, Related to Figure 5

(A) Normalized number of EGFP positive and negative dysplastic and regenerative nodules, HCA and HCC derived from hURI-tetOFF^{hep} crossed with SA^{CreERT2} ; R26-stop-EYFP and hURI-tetOFF^{hep} crossed with Sox9^{CreERT2}; R26-stop-EYFP.

(B) Statistical analysis of normalized number of tumors from (A).

(C) Table summarizing all tumors obtained from different carcinogenesis models.

(D) Histogram representing HSP70 classification used to determine HAGCKS score in HCC derived from hURI-tetOFF^{hep}; SA^{CreERT2}; R26-stop-EYFP mice.

(E) Histogram representing HSP70 classification used to determine HAGCKS score in HCA derived from hURI-tetOFF^{hep}; SA^{CreERT2}; R26-stop-EYFP mice.

(F) Representative images of HSP70 IHC showing staining classified as low (1), intermediate (2) and high (3).

(G) Histogram representing AFP classification used to determine HAGCKS score in HCC derived from hURI-tetOFF^{hep}; SA^{CreERT2}; R26-stop-EYFP mice.

(H) Histogram representing AFP classification used to determine HAGCKS score in HCA derived from hURI-tetOFF^{hep}; SA^{CreERT2}; R26-stop-EYFP mice.

(I) Representative images of AFP IHC showing staining classified as positive (1).

(J) Histogram representing GS classification used to determine HAGCKS score in HCC derived from hURI-tetOFF^{hep}; SA^{CreERT2}; R26-stop-EYFP mice.

(K) Histogram representing GS classification used to determine HAGCKS score in HCA derived from hURI-tetOFF^{hep}; SA^{CreERT2}; R26-stop-EYFP mice.

(L) Representative images of GS IHC showing staining classified as low (1), intermediate (2) and high (3).

(M) Histogram representing CK19 classification used to determine HAGCKS score in HCC derived from hURI-tetOFF^{hep}; SA^{CreERT2}; R26-stop-EYFP mice.

(N) Histogram representing CK19 classification used to determine HAGCKS score in HCA derived from hURI-tetOFF^{hep}; SA^{CreERT2}; R26-stop-EYFP mice.

(O) Representative images of CK19 IHC showing negative (0) and positive (1) staining.

(P) Histogram representing Sox9 classification used to determine HAGCKS score in HCC derived from hURI-tetOFF^{hep}; SA^{CreERT2}; R26-stop-EYFP mice.

(Q) Histogram representing Sox9 classification used to determine HAGCKS score in HCA derived from hURI-tetOFF^{hep}; SA^{CreERT2}; R26-stop-EYFP mice.

(R) Representative images of Sox9 IHC showing staining classified as low (1), intermediate (2) and high (3).

(S) Histogram representing Ki67 classification used to determine HAGCKS score in HCC derived from hURI-tetOFF^{hep}; SA^{CreERT2}; R26-stop-EYFP mice.

(T) Histogram representing Ki67 classification used to determine HAGCKS score in HCA derived from hURI-tetOFF^{hep}; SA^{CreERT2}; R26-stop-EYFP mice.

(U) Representative images of Ki67 IHC showing staining classified as low (1), intermediate (2) and high (3).

(V) Representative images of H&E and IHC with different markers used in HAGCKS score in HCC or HCA derived from hURI-tetOFF^{hep}; SA^{CreERT2}; R26-stop-EYFP mice.

(W) Pie charts representing type of HCC derived from hepatocytes in Mdr2^{KO} mice crossed with R26-stop-ZsGreen mice and infected with AAV8-Tbg^{Cre} (n = 7) (left chart) or in R26-mTOM-

stop-mGFP mice infected with AAV8-Tgb^{Cre} and treated with DEN and CCl_4 (n = 42) mice (right chart).

Scale bars represent 100 μm.

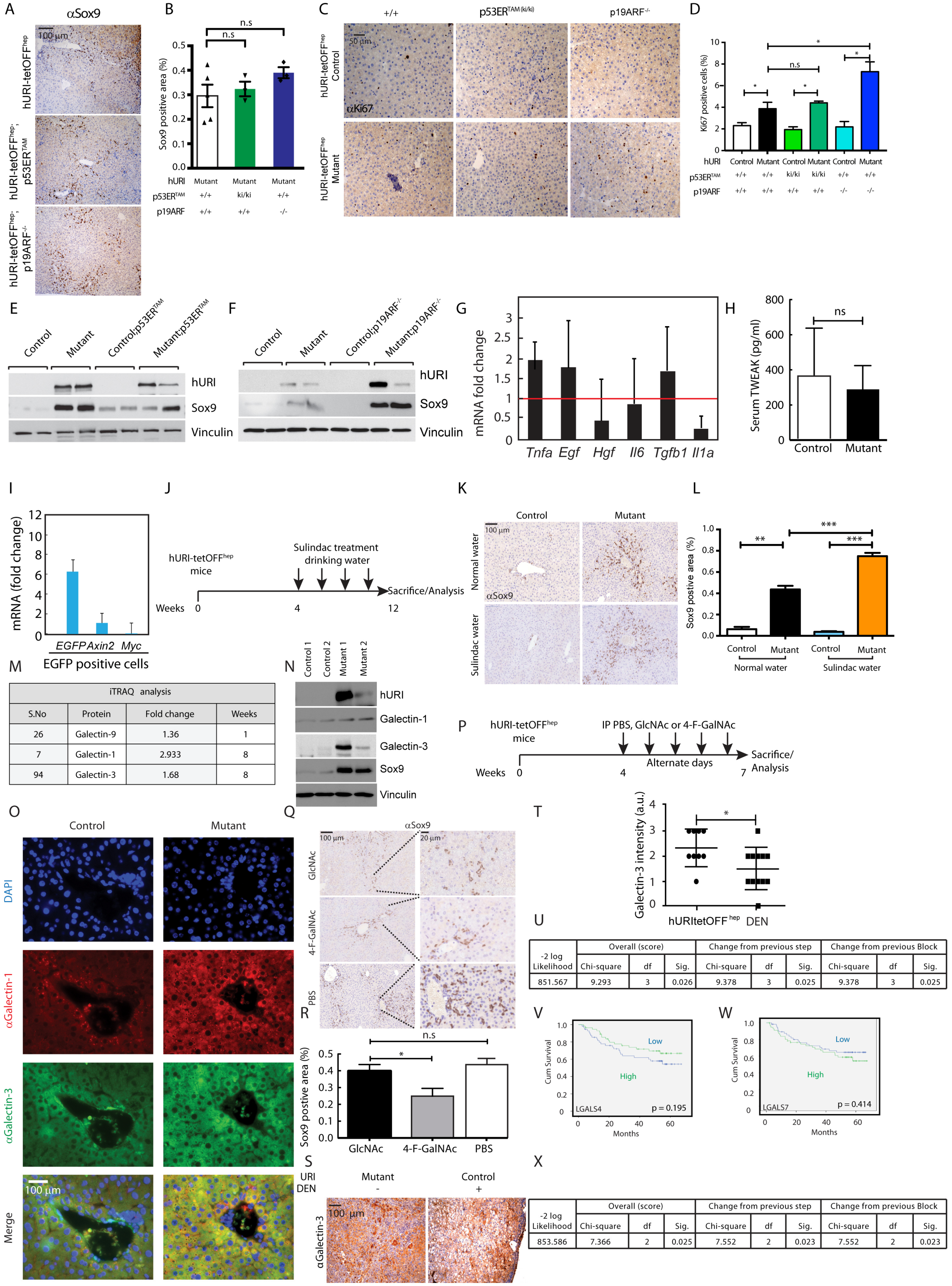

M

iTRAQ analysis			
S.No	Protein	Fold change	Weeks
26	Galectin-9	1.36	1
7	Galectin-1	2.933	8
94	Galectin-3	1.68	8

U

-2 log Likelihood	Overall (score)			Change from previous step			Change from previous Block		
	Chi-square	df	Sig.	Chi-square	df	Sig.	Chi-square	df	Sig.
851.567	9.293	3	0.026	9.378	3	0.025	9.378	3	0.025

X

-2 log Likelihood	Overall (score)			Change from previous step			Change from previous Block		
	Chi-square	df	Sig.	Chi-square	df	Sig.	Chi-square	df	Sig.
853.586	7.366	2	0.025	7.552	2	0.023	7.552	2	0.023

Figure S6. Hepatocytes Expressing Oncogenic URI Produce Galectin-3 to Instruct and Activate HPCs, Related to Figure 6

(A) Representative images of Sox9 staining in liver sections derived from 12-week-old hURI-tetOFF^{hep} , hURI-tetOFF^{hep} ; p53ER^{TAM} and hURI-tetOFF^{hep} ; p19ARF$^{-/-}$ mice.

(B) Quantification of Sox9 IHC from (A). $n \geq 4$.

(C) Representative images of Ki67 staining in liver sections derived from 12-week-old hURI-tetOFF^{hep} , hURI-tetOFF^{hep} ; p53ER^{TAM} and hURI-tetOFF^{hep} ; p19ARF$^{-/-}$ mice.

(D) Quantification of Ki67 IHC from (C). $n \geq 4$.

(E) WB analysis of Sox9 expression in 12-week-old hURI-tetOFF^{hep} and hURI-tetOFF^{hep} ; p53ER^{TAM} mice.

(F) WB analysis of Sox9 expression in 12-week-old hURI-tetOFF^{hep} and hURI-tetOFF^{hep} ; p19ARF$^{-/-}$ mice.

(G) qRT-PCR analysis of *Tnfa*, *Egf*, *Hgf*, *Il6*, *Tgfb1* and *Il1a* mRNA in livers derived from 8-week-old hURI-tetOFF^{hep} mice. n = 4. Error bars represent ± SD.

(H) Serum levels of TWEAK cytokine in 8-week-old hURI-tetOFF^{hep} mice. n = 3.

(I) qRT-PCR analysis of *Egfp*, *Axin2* and *Myc* mRNA from FACS-sorted SPCs from livers derived from 12-week-old hURI-tetOFF^{hep} ; Sox9$^{IRES\text{-}EGFP}$ mice. n = 3. Error bars represent ± SD.

(J) Scheme representing sulindac-treated hURI-tetOFF^{hep} mice. Mice were treated for 8 weeks since 4 weeks of age with 0.18 g/L sulindac in drinking water.

(K) Representative images of Sox9 staining in liver sections derived from 12-week-old hURI-tetOFF^{hep} mice treated either with normal drinking water or with drinking water containing 0.18 g/L of sulindac.

(L) Percentage of Sox9-positive area from liver sections obtained from mice described in (J). n = 3 for controls and n = 4 for mutants under water, respectively. n = 4 and n = 6 for controls and mutants treated with sulindac, respectively.

(M) Table showing the fold change and rank (S. No) of galectin-9, galectin-1 and galectin-3 detected among differentially expressed proteins analyzed by iTRAQ performed in livers from 1- and 8-week old hURI-tetOFF^{hep} mice as previously described (Tummala et al., 2014).

(N) Western blot analysis in 12-week-old hURI-tetOFF^{hep}. Immunoblots are performed using the indicated antibodies.

(O) Co-immunofluorescence of galectin-1 and galectin-3 in liver sections derived from 8-week-old hURI-tetOFF^{hep} mice. n = 5.

(P) Scheme representing GlcNAc- and 4-F-GalNAc-treated hURI-tetOFF^{hep} mice. At 4 weeks of age mice received I.P injection of either GlcNAc or 4-F-GalNAc every second day for 2 weeks.

(Q) Representative images of Sox9 staining in livers sections obtained from mice treated as described in (P). n = 7.

(R) Quantification of Sox9-positive area in livers described in (P). n = 7.

(S) Representative images of galectin-3 IHC in the tumors derived from hURI-tetOFF^{hep} or DEN treated mice.

(T) Quantification of IHC from (S). n = 9 for hURI-tetOFF^{hep}-derived tumors. n = 12 for DEN-derived tumors.

(U) Multivariate Cox regression survival analysis for all the human galectin isoforms (*LGALS3*, *LGALS4* and *LGALS7*) in 221 patient human HCC gene expression analyses. (p=0.025). "df" and "Sig." represents degrees of freedom and significance respectively.

(V) Kaplan Meier curve analysis of human HCC patient cumulative survival based on the expression of *LGALS4.* Degrees of freedom: 1, Chi-square: 1.680, $p = 0.195$.

(W) Kaplan Meier curve analysis of human HCC patient cumulative survival based on the expression of *LGALS7.* Degrees of freedom: 1, Chi-square: 0.667 $p = 0.414$.

(X) Multivariate Cox regression survival analysis for *LGALS3* and *MYC* in 221 patient human HCC gene expression analyses. ($p = 0.023$). “df” and “Sig.” represents degrees of freedom and significance respectively.

Data are represented as mean ± SEM.

Scale bars represents 20 μm, 50 μm and 100 μm.

ns = non significant, $p \leq 0.05$ = *, $p \leq 0.01$ = ** and $p \leq 0.001$ = ***.

A

S.No	Protein name	Symbol	Fold change 1 week	Fold change 8 weeks
1	Glycerol-3-phosphate dehydrogenase	Gpd1	1.469	0.855
2	Pyruvate kinase isozymes R/L	Pk	1.463	0.657
3	Ketohexokinase	Khk	1.448	0.692
4	Glucose-6-phosphate 1-dehydrogenase	G6pd	0.708	1.416
5	Glutaminase liver isoform, mitochondrial	Gls2	0.664	0.330
6	L-lactate dehydrogenase B chain	Ldh	0.502	1.653
7	Phosphoacetylglucosamine mutase	Pgm3	1.266	1.609
8	L-Lactic dehydrogenase/lactate dehydrogenase	Ldh	0.502	1.653
9	Pyruvate kinase isozymes M1/M2	Pkm	0.843	1.677
10	Pyruvate dehydrogenase [lipoamide]] kinase isozyme 2, mitochondrial	Pdhb	0.989	0.797
11	2-oxoglutarate dehydrogenase E1 component, mitochondrial	Dhktd1	0.983	0.429
12	Succinyl-CoA ligase [GDP-forming] subunit beta, mitochondria	Suclg	1.053	0.673
13	Succinate dehydrogenase cytochrome b560 subunit, mitochondrial	Sdhc	1.020	0.703
14	Pyruvate carboxylase, mitochondrial	Pcx	1.102	0.697

B

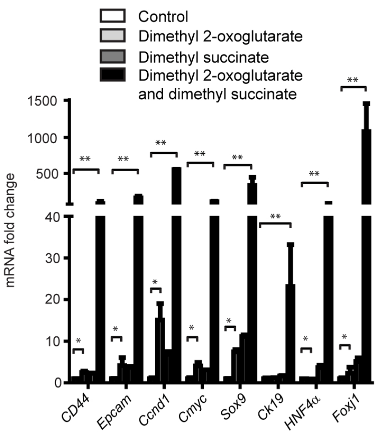

C

-2 log Likelihood	Overall (score) Chi-square	df	Sig.	Change from previous step Chi-square	df	Sig.	Change from previous Block Chi-square	df	Sig.
846.388	14.175	9	0.116	14.750	9	0.098	14.750	9	0.098

D

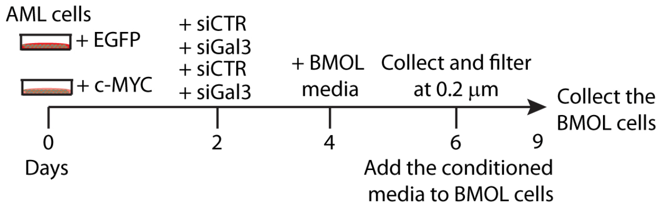

E

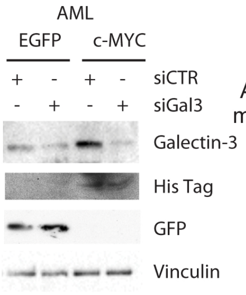

F

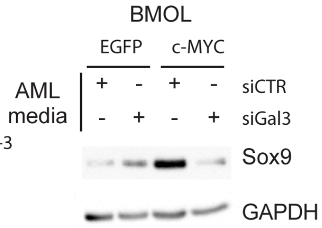

G

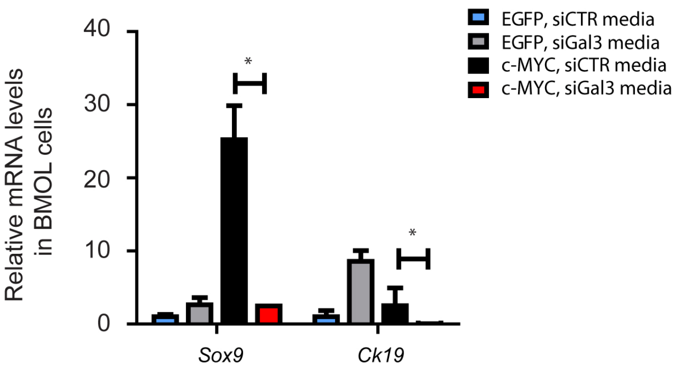

Figure S7. Hepatocytes Expressing Oncogenic URI Produce α-ketoglutarate and Galectin-3 to Preserve HPC Undifferentiated State, Related to Figure 7

(A) Table representing the differentially regulated proteins of intermediary metabolism obtained by iTRAQ analysis 1- and 8-week old mice previously described (Tummala et al., 2014).

(B) qRT-PCR analysis of BMOL cells treated with either 1 mM dimethyl 2-oxoglutarate (dimethyl-α-ketoglutarate), 4 mM dimethyl succinate or both for 24 h.

(C) Multivariate Cox regression analysis for the enzymes of TCA cycle (*IDH1*, *IDH2*, *IDH3A*, *IDH3B*, *IDH3G*, *OGDH*, *SUCLA2*, *SDHB* and *PC*) down regulated in hURItetOFF^{hep} mouse model. (p=0.098). "df" and "Sig." represents degrees of freedom and significance respectively.

(D) Schematic representation of AML-12 and BMOL cell treatment. Media from EGFP or His-c-MYC overexpressing AML-12 cells transfected either with siCTR or siGal3 was used to culture BMOL cells for 3 days.

(E) WB analysis of AML-12 cells from (D). Membranes were blotted with the indicated antibodies.

(F) WB of BMOL cells after 72 hr incubation with media from AML-12 cells stably overexpressing EGFP or His-c-MYC and transfected with either siCTR or siGal3. Membrane was blotted with the indicated antibodies.

(G) qRT-PCR analysis of BMOL cells cultured with media from AML-12 cells as described in (D).

Data are represented as mean ± SEM. $p \leq 0.05$ = *; $p \leq 0.01$ = **.

Supplemental Experimental Procedures

Antibodies

Antibodies used for immunoblotting, immunofluorescence and immunohistochemistry were as follows: phospho-H2AX (Ser-139) (1:1000) (05-636), Cyp2E1 (1:200) (AB1252) and Sox9 (1:1000) (AB5535) were purchased from Millipore. CK19 (1:50) Troma-III was from CNIO Monoclonal Antibodies Core Unit. Glutamine synthetase (1:200) (G2781), His (1:100) (11922416001) and vinculin (1:1000) (V9131) were from Sigma Aldrich. Heat shock protein 70 (1:200) (sc-24) was from Santa Cruz Biotechnology. CD44 PE-Cy7 (1:100) (552849) was purchased from BD Pharmingen. CD326 (EpCAM)-APC (1:100) (17-5791) and CD133 (Prominin I) PerCP-eFluor 710 (1:100) (46-1331) were from eBioscience. GPR49 (Lgr5) (1:50) (ab75732), GFP (1:1000) (ab290), Laminin (1:200) (ab11575) and HNF4α (ab199431) were Abcam. DLK1 (1:100) (AF8277) and AFP (1:200) (AF5369) were from R&D systems. Galectin-1 (1:1000) (11858-1-AP) and Galectin-3 (1:1000) (60207-1-Ig) were purchased from Proteintech. Ki67 (1:1) (# MAD-000310QD) was purchased from Master Diagnostica. URI antibodies have been previously described (Buren et al., 2016; Tummala et al., 2014).

Generation and Handling of Mice

hURI conditional knock-in (hURI-tetOFF^{hep}) was generated as recently reported (Tummala et al., 2014). Serum albumin (SA)-CreERT2 (Schuler et al., 2004), Sox9$^{IRES\text{-}CreERT2}$ (Furuyama et al., 2010) and Sox9$^{IRES\text{-}EGFP}$ (Nel-Themaat et al., 2009; Soeda et al., 2010) mice were kindly donated by Pierre Chambon, Yoshiya Kawaguchi and Haruhiko Akiyama, respectively. R26-stop-EYFP reporter line was previously reported and present in house (Srinivas et al., 2001). p19ARF$^{-/-}$

mouse was a gift from Manual Serrano. p53 inactivation in the hURI-tetOFF^{hep} mouse was performed by crossing hURI-tetOFF^{hep} mice with p53ER^{TAM} mice, in which p53 activation requires ectopic 4-hydroxytamoxifen provision (Christophorou et al., 2005). Lgals3$^{\Delta/\Delta}$ mice were kindly obtained from Alison Mackinnon. R26-mTOM-stop-mGFP mice were previously reported (Muzumdar et al., 2007) and infected with AAV8-Tgb^{Cre} and treated with DEN and CCl_4 as reported (Mu et al., 2015). Mdr2^{KO} mice crossed with R26-stop-ZsGreen mice and infected with AAV8-Tgb^{Cre} and CK19^{CreERT2} ; R26-mTOM-stop-mGFP mice treated with DEN and CCl_4 were previously described (Mu et al., 2015). All mice have been housed in pathogen-free conditions. All experiments were approved by the CNIO-ISCIII Ethics Committee and performed in accordance with the guidelines for ethical conduct in the care and use of animals as stated in the international guiding principles for biomedical research involving animals, developed by the Council for International Organizations of Medical Sciences. Littermates were always used as controls. No gender differences were observed and age/developmental stage of mice was included appropriately in text and Figure legends. Food (Harlan Laboratories and Research Diets Inc.) and water were provided *ad libitum.*

Mouse Treatment

For diethylnitrosamine (DEN)-induced carcinogenesis, 14-day-old mice were injected intraperitoneally with 25 mg/kg of DEN (Sigma) according to the previously described protocol (Vesselinovitch and Mihailovich, 1983). After DEN injection, for tracking EYFP positive cells, Cre-mediated recombinase was activated by feeding mice with tamoxifen diet at the concentration of 400 mg/kg (supplemented in food) since weaning and for the next 2 weeks. Then after, mice were transferred to chow diet. Mice were sacrificed at the indicated time points

and the relative contribution of different cell types was assessed by histological methods and quantified.

N-Acetyl-D-glucosamine (GlcNAc) (Sigma-Aldrich (A8625)) and 4-Fluoro-N-acetylgalactosamine (4-F-GalNAc) (Sussex research laboratories (C8H14FNO6)) were dissolved in PBS. 3- or 4-week-old hURI-tetOFF^{hep} mice were injected with 100 mg/kg of GlcNAc or 4-F-GalNAc intraperitoneally every second day for 3 weeks.

Nicotinamide riboside (NR) (97% purity, Waterstonetech Pharma) was mixed with ice-cold powdered food as previously reported (Tummala et al., 2014)

Sulindac (99% purity, Shanghai Moda Chemicals Co., Ltd) was dissolved in drinking water at the concentration of 0.18 g/L, and given to 4-week-old hURI-tetOFF^{hep} mice for 8 weeks. Sulindac was changed every second day and freshly prepared.

Doxycyline was supplied in the diet ad libitum as previously described (Gomes et al., 2016; Tummala et al., 2014).

Cell Culture, Transfection, Cell Number Counts

Non tumorigenic alpha mouse liver (AML)-12 cells were grown in DMEM/F12 media supplemented with 10% fetal calf serum, 100 units/ml penicillin and 0.1 mg/ml streptomycin, 0.005 mg/ml insulin, 0.005 mg/ml transferrin, 5 ng/ml selenium, and 40 ng/ml dexamethasone purchased from Gibco (Wu etal., 1994). BMOL cells were cultured in Williams E media (Sigma-Aldrich) supplemented with 10% fetal calf serum, 100 units/ml of penicillin and 0.1 units/ml of streptomycin (Gibco), 30 ng/ml insulin-like growth factor (Novo enzymes), 20 ng/ml epidermal growth factor (BD Biosciences), 2 mM L-Glutamine (Sigma-Aldrich) and 0.25 U/ml Humulin R (Eli Lilly). Stable AML-12 cell lines overexpressing EGFP, HA-URI and His-c-Myc were

obtained by plasmid transfections using FUGENE 6 following the manufacturer instructions followed by 2 μg/ml puromycin selection for 3 days.

ON-TARGET plus SMART pool siRNA targeting murine Galectin-3 (siGal3) and control siRNA (siCTR) were purchased from Dharmacon. pcDNA3.1-HA-URI pcDNA-EGFP and pD40-His/V5-c-Myc and pcDNA-EGFP were previously described (Buren et al., 2016; Djouder et al., 2007). siRNA transfections were performed using Lipofectamine 3000 RNAiMAX following the manufacturer's instructions. For co-culture experiments, AML-12 cells were transfected with either siCTR or siGal3 or treated with either 10 μM BPTES or DMSO for 48 hr. Afterwards media was replaced by Williams E media and incubated for 24 hr. Pre-incubated media was then transferred to BMOL cells. Cells were lysed 72 hr later and processed for either protein or RNA isolation. BMOL cells were treated with 1 mM dimethyl 2-oxyglutarate (Sigma-Aldrich) or 4 mM dimethyl succinate (Sigma-Aldrich) or both for 24 hours. Cell number was measured in triplicates at the indicated times using Neubauer chamber.

Flow Cytometry of HPCs

Isolation

Liver isolated from mice was placed in ice cold DMEM/F12 and cut into small pieces with a scalpel. The obtained liver pulp was incubated in 10 ml of the enzyme solution containing 5 mg collagenase, 5 mg pronase and 1 ml DNAse in PBS in 37 °C water bath for 30 min to digest the tissue. Digested fraction was passed through 70 micron mesh filter and suspended in 20 ml of DMEM/F12 media supplemented with 10% FBS. Isolated cells were centrifuged 1 min at 50 g at 4 °C and the supernatant was collected. Step was repeated until no hepatocyte pellet was visible. Final supernatant fraction was centrifuged 8 min at 180 g in 4 °C to obtain fraction excluding

hepatocytes. Pellet was incubated for 5 min in 4 °C with 1 ml of red blood cell lysis solution to remove erythrocytes. Cells were centrifuged for 5 min at 200 g, pellet was used for fluorescence activated cell sorting (FACS).

Fluorescence Activated Cell Sorting (FACS) Procedure

Isolated cells were resuspended in 1ml of MACS buffer (0.1% BSA, 2 mM EDTA in Ca^{2+}/Mg^{2+} free PBS) and incubated for 20 min on ice with Fc block (rat anti-mouse cd16/cd32) at the concentration of 1:200. Afterwards cells were washed with 300 µl of MACS buffer for 5 min and centrifuged for 2 min at 200 g at 4°C. Pellet was resuspended in 200 µl of MACS buffer and incubated for 20 min at 4 °C with primary antibodies (CD44 1:50, EpCAM 1:50, CD133-Percp 1:50, LGR5 1:10, DLK1 1:50). Then after cells were washed 2 times with MACS buffer, resuspended in 300 µl of MACS buffer and incubated for 15 min at 4 °C with secondary antibodies (Alexa-Fluor-680 1:1000, Alexa-Fluor-555 1:1000). Then, cells were washed 2 times with MACS buffer and resuspended in 300 µl of MACS buffer. DAPI (1:100) was added and, cells were passed through a filter and processed for FACS analysis using BD LSRFortessa machine.

Compensation beads were prepared for CD133-PE-Cy7, EpCAM-APC and CD44-Percp antibodies raised in mouse and rat using OneComp ebeads (ebiosciences). Compensation for DLK1 and LGR5 was prepared by using the cells from the control (not containing fluorophore) and primary antibodies (DLK1 1:50, LGR5 1:10)

FMO (Fluorescence minus one) was prepared separately for each primary antibody used. 100 µl of cells isolated from each mutant was mixed together and 100 µl of the mixture was used for each FMO. Cells were resuspended in 100 µl of MACS buffer and incubated for 20 min in 4 °C

with primary antibodies according to the FMO prepared (CD44 1:50, EpCAM 1:50, CD133-Percp 1:50, LGR5 1:10, DLK1 1:50). Cells were washed with 300 μl of MACS buffer, resuspended in 300 μl of MACS buffer and if necessary for the FMO incubated for 15 min in 4°C with secondary antibody (Alexa-Fluor-680 1:1000, Alexa-Fluor-555 1:1000). Cells were washed 2 times with MACS buffer and resuspended in 300 μl of MACS buffer. DAPI (1:100) was added, cells were passed through a filter and processed for FACS analysis.

FACS Analysis

FACS data were analyzed using FlowJo (www.flowjo.com) software using the single color FMO's and sample data acquired without any fluorophore as control.

Genotyping and qRT-PCR

For genotyping toe DNA was extracted by overnight incubation of toes with 500 μl of the cell lysis buffer (1% SDS, 0.1 M NaCl, 0.1 M EDTA, 0.05 M Tris (pH 8) and 400 μg/ml of proteinase K). Extracted DNA after saturated salt precipitation was precipitated using ice-cold isopropanol which was further washed with 80% ethanol. The DNA pellet was further dried and resuspended in 400 μl of distilled water. 1 μl of DNA was used for genotyping. For the *ColhURI* locus the following primers were used: P1-F: GCACAGCATTGCGGACATGC; P2-R: CCCTCCATGTGTGACCAAGG; P3-R: GCAGAAGCGCGGCCGTCTGG. For the *LaptTA* transactivator locus the following primers were used: P1-F: TCTGAGCATGGCCTCTAA; P2-R: GCTGGAGTAAATTTCACAGTG; P3-R: TCTCACTCGGAAGGACAT. For $SA^{CreERT2}$ the following primers were used: P1: ATCATTTCTTTGTTTTCAGG, P2: GGAACCCAAACTGATGACCA and P3: TTAAACAAGCAAAACCAAAT. For $Sox9^{IRES-}$

CreERT2 the following primers were used: P1: TCCAATTTACTGACCGTACAC CAA and P2: CCTGATCCTGGCAATTTCGGCTA. For R26: P1: TGACCCTGAAGTTCATCTGCA and P2: TCACGAACTCCAGCAGGACCA. For EYFP: P1: TGACCCTGAAGTTCATCTGCA and P2: TCACGAACTCCAGCAGGACCA. For *Lgals3Δ/Δ*: P1: GAGGAGGGTCAAAGGGAAAG, P2: GACTGGAATTGCCCATGAAC and P3: TCGCCTTCTTGACGAGTTCT.

For qRT-PCR, total RNA was extracted from 20-50 mg of liver or cells as described in (Tummala et al., 2014) and qRT-PCR was performed with the following primers. Human-*Uri*-F: GAAAGACTCAGCACCTTGCC. Human-*Uri*-R: TTCCGGTGCTCAACTAAACC. Mouse-*Myc*-F: TGACCTAACTCGAGGAGGAGCTGGAATC, Mouse-*Myc*-R: AAGTTTGAGGCAGTTAAAATTATGGCTGAAGC. Mouse-*Axin*-F: GAATCTGCATGGGCAACC . Mouse-*Axin*-R: GAATCCTGGTACATCTGGGAAC. *Gfp*-F: TGACCCTGAAGTTCATCTGCA. *Gfp*-R: TCACGAACTCCAGCAGGACCA. Mouse-*TNFA*–F: AGTTCTATGGCCCAGACCCT. Mouse-*TNFA*-R: CGGACTCCGCAAAGTCTAAG. Mouse-*Egf*–F: ACATAGATGGAATGGGCACAGG. Mouse-*Egf* –R: CCTCTAGAAATGTGGTATGGCTG. Mouse-*Hgf*–F: TTCCCAGCTGGTCTATGGTC Mouse-*Hgf*–R: GGTGCTGACTGCATTTCTCA. Mouse-*Il6*–F: GCTACCAAACTGGATATAATCAGGA Mouse-*Il6*–R: CCAGGTAGCTATGGTACTCCAGAA. Mouse-*Tgfb*–F: CGCCCGGGTTGTGTTGGTTGTAGA Mouse-*Tgfb*–R: CTGACCCCCACTGATCCTGAG. Mouse-*Il1a*–F: AAGATGTCCAACTTCACCTTCAAGGAGAGCCG. Mouse-*Il1a*–R: AGGTCGGTCTCACTAACTGTGATGAGTTTTGG Mouse-*Actb*-F: CACAGCTGAGAGGGAAATCG. Mouse-*Actb*-R: AGTTTCATGGATGCCACAGG. Mouse-

Cd44-F: TGCATTTGGTGAACAAGGAA. Mouse-*Cd44*-R: GGAATGACGTCTCCAATCGT. Mouse-*Epcam*-F: GCGGCTCAGAGAGACTGTG. Mouse-Epcam-R: CCAAGCATTTAGACGCCAGTTT. Mouse-*Ccnd1*-F: TCAAGTGTGACCCGGACTG. Mouse-*Ccnd1*-R: ATGTCCCACATCTCGCACGTC. Mouse-*Sox9*-F: TGCCCATGCCCGTGCGCGTCAA. Mouse-*Sox9*-R: CGCTCCGCCTCCTCCACGAAGGGTCT. Mouse-*Ck19*-F: GGACCCTCCCGAGATTACAACCA. Mouse-*Ck19*-R: GCCAGCTCCTCCTTCAGGCTCT. Mouse-*Hnf4a*-F: CGGGCTGGCATGAAGAAGGAAG. Mouse-*Hnf4a*-R: TGGGAGAGGTGATCTGCTGGGA. Mouse-*Foxj1*-F: CCGCCATGCAGACCCCACCTGGCA. Mouse-*Foxj1*-R: AGGCCCCACTGAGCAGGCGCTCT

Supplemental References

Buren, S., Gomes, A.L., Teijeiro, A., Fawal, M.A., Yilmaz, M., Tummala, K.S., Perez, M., Rodriguez-Justo, M., Campos-Olivas, R., Megias, D*., et al.* (2016). Regulation of OGT by URI in Response to Glucose Confers c-MYC-Dependent Survival Mechanisms. Cancer Cell *30*, 290-307.

Christophorou, M.A., Martin-Zanca, D., Soucek, L., Lawlor, E.R., Brown-Swigart, L., Verschuren, E.W., and Evan, G.I. (2005). Temporal dissection of p53 function in vitro and in vivo. Nat Genet *37*, 718-726.

Djouder, N., Metzler, S.C., Schmidt, A., Wirbelauer, C., Gstaiger, M., Aebersold, R., Hess, D., and Krek, W. (2007). S6K1-mediated disassembly of mitochondrial URI/PP1gamma complexes

activates a negative feedback program that counters S6K1 survival signaling. Mol Cell *28*, 28-40.

Muzumdar, M.D., Tasic, B., Miyamichi, K., Li, L., and Luo, L. (2007). A global double-fluorescent Cre reporter mouse. Genesis *45*, 593-605.

Nel-Themaat, L., Vadakkan, T.J., Wang, Y., Dickinson, M.E., Akiyama, H., and Behringer, R.R. (2009). Morphometric analysis of testis cord formation in Sox9-EGFP mice. Dev Dyn *238*, 1100-1110.

Schuler, M., Dierich, A., Chambon, P., and Metzger, D. (2004). Efficient temporally controlled targeted somatic mutagenesis in hepatocytes of the mouse. Genesis *39*, 167-172.

Soeda, T., Deng, J.M., de Crombrugghe, B., Behringer, R.R., Nakamura, T., and Akiyama, H. (2010). Sox9-expressing precursors are the cellular origin of the cruciate ligament of the knee joint and the limb tendons. Genesis *48*, 635-644.

Srinivas, S., Watanabe, T., Lin, C.S., William, C.M., Tanabe, Y., Jessell, T.M., and Costantini, F. (2001). Cre reporter strains produced by targeted insertion of EYFP and ECFP into the ROSA26 locus. BMC Dev Biol *1*, 4.

Tummala, K.S., Gomes, A.L., Yilmaz, M., Grana, O., Bakiri, L., Ruppen, I., Ximenez-Embun, P., Sheshappanavar, V., Rodriguez-Justo, M., Pisano, D.G*., et al.* (2014). Inhibition of de novo NAD(+) synthesis by oncogenic URI causes liver tumorigenesis through DNA damage. Cancer Cell *26*, 826-839.

Vesselinovitch, S.D., and Mihailovich, N. (1983). Kinetics of diethylnitrosamine hepatocarcinogenesis in the infant mouse. Cancer research *43*, 4253-4259.
